# Phenotypic divergence of traits that mediate antagonistic and mutualistic interactions between island and continental populations of the tropical plant, *Tribulus cistoides* (Zygophyllaceae)

**DOI:** 10.1002/ece3.9766

**Published:** 2023-03-22

**Authors:** Winer Daniel Reyes‐Corral, Sofia Carvajal‐Endara, Molly Hetherington‐Rauth, Jaime A. Chaves, Peter R. Grant, B. Rosemary Grant, Andrew P. Hendry, Marc T. J. Johnson

**Affiliations:** ^1^ Department of Biology and Redpath Museum McGill University Montréal Québec Canada; ^2^ Centro de Investigación en Biodiversidad y Cambio Climático (BioCamb), Ingeniería en Biodiversidad y Recursos Genéticos, Facultad de Ciencias del Medio Ambiente Universidad Tecnológica Indoamérica Quito Ecuador; ^3^ Department of Biology University of Toronto Mississauga Mississauga Ontario Canada; ^4^ Colegio de Ciencias Biológicas y Ambientales Universidad San Francisco de Quito Quito Ecuador; ^5^ Department of Biology Hensill Hall San Francisco California USA; ^6^ Department of Ecology and Evolutionary Biology Princeton University Princeton New Jersey USA

**Keywords:** Darwin's finches, flower morphology, fruit morphology, herbarium collections, mericarp, phenotypic divergence, phenotypic variation, pollinators, seed predation, *Tribulus cistoides*

## Abstract

Island systems have long served as a model for evolutionary processes due to their unique species interactions. Many studies of the evolution of species interactions on islands have focused on endemic taxa. Fewer studies have focused on how antagonistic and mutualistic interactions shape the phenotypic divergence of widespread nonendemic species living on islands. We used the widespread plant *Tribulus cistoides* (Zygophyllaceae) to study phenotypic divergence in traits that mediate antagonistic interactions with vertebrate granivores (birds) and mutualistic interactions with pollinators, including how this is explained by bioclimatic variables. We used both herbarium specimens and field‐collected samples to compare phenotypic divergence between continental and island populations. Fruits from island populations were larger than on continents, but the presence of lower spines on mericarps was less frequent on islands. The presence of spines was largely explained by environmental variation among islands. Petal length was on average 9% smaller on island than continental populations, an effect that was especially accentuated on the Galápagos Islands. Our results show that *Tribulus cistoides* exhibits phenotypic divergence between island and continental habitats for antagonistic traits (seed defense) and mutualistic traits (floral traits). Furthermore, the evolution of phenotypic traits that mediate antagonistic and mutualistic interactions partially depended on the abiotic characteristics of specific islands. This study shows the potential of using a combination of herbarium and field samples for comparative studies on a globally distributed species to study phenotypic divergence on island habitats.

## Graphical abstract

Tribulus cistoides is a plant common and widespread in tropical areas on multiple continents and island systems. Unique island interactions, such as endemic vertebrate granivores (antagonists) and pollinators (mutualists), may shape the phenotypes (fruits and flowers) that mediate these interactions of Tribulus on island populations. Using field‐collected and herbarium samples, we found that fruits and flowers on island populations are diverging phenotypically.

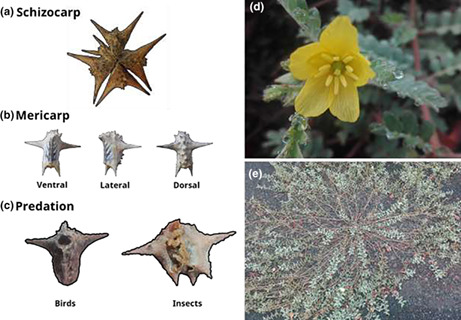

## 1 INTRODUCTION

Islands have long served as models for understanding the processes that shape the evolution of life. Species living on islands provide powerful systems for testing evolutionary hypotheses and theories (Bramwell & Caujapé‐Castells, 2011; Losos & Ricklefs, 2009; Whittaker & Fernandez‐Palacios, 2007). The appeal of island systems comes from their unique species diversity and simplified species interactions, making it easier to identify the drivers of adaptive evolution (Barrett, 1996; Grant, 1998; Traveset & Navarro, 2018). Moreover, large differences in the biotic and abiotic environments between island and continental habitats can lead to divergent selection between conspecific populations, potentially leading to phenotypic differentiation of island populations and speciation (Whittaker & Fernandez‐Palacios, 2007). Here we compared conspecific populations of a globally distributed tropical plant, *Tribulus cistoides* L. (Zygophyllaceae), to understand whether divergent antagonistic and mutualistic communities between islands and continental habitats drive divergent phenotypic plant traits that mediate species interactions.

Island and continental habitats frequently differ in their biotic communities. Islands typically have fewer native mammalian herbivores, favoring birds and reptiles with high dispersal capacity over water (Burns, 2019). This discrepancy can lead to the evolutionary loss of antiherbivore defenses in plants (Baier & Hoekstra, 2019; Cummins et al., 2020). For example, spines largely evolve as protection against vertebrate herbivores, as in the case of the Island Bush Poppy (*Dendromecon rigida harfordii*) on the Island of Santa Cruz, California, where these plants evolved reduced spines due to a historical lack of herbivores (Bowen & Vuren, 1997). However, the loss of antiherbivore defenses on islands is not universal for all species (Meredith et al., 2019; Monroy & García‐Verdugo, 2019; Moreira et al., 2021). The Hawaiian Prickly Poppy (*Argemone glauca*) evolved greater spine density than their continental sister species (*A. mexicana*), putatively because of selection by an extinct herbivorous duck that was common in Hawaii (Hoan et al., 2014). Additionally, a recent meta‐analysis of plant defenses found no significant difference in either plant physical or chemical defenses between insular and continental plant populations, and in fact, there was a trend for physical defenses to be higher on islands (Moreira et al., 2021). This range of results shows how variation in antagonistic interactions between island and continental communities can influence the evolutionary processes of defense traits. However, there is still the need for more studies of insular plant–animal interactions to understand the conditions that lead to the evolution of increased versus decreased defenses on islands compared with continental populations.

Mutualistic interactions also frequently differ between island and continental communities, with the diversity of mutualists (e.g., pollinators and dispersers) typically being lower on islands. It is often predicted that the absence of mutualistic species could lead to the loss of traits that mediate mutualistic species interactions on islands (Janzen, 1973). Specifically, in the case of pollination, pollinators tend to be less diverse and less specialized on islands than on the continent (Barrett, 1996; Burns, 2019; Traveset & Navarro, 2018). Less specialized pollinators can give an advantage to more generalized flowers, and lead to the evolution of selfing and wind‐pollination and thus smaller attractive structures (Bramwell & Caujapé‐Castells, 2011; Burns, 2019; Carlquist, 1965). Various studies support these observations (Inoue & Amano, 1986; Martén‐Rodríguez et al., 2015; Yamada et al., 2010). However, as with antiherbivore defenses, there is a large variation in results, calling into question whether general predictions can be made. A recent comparative analysis between continental insect‐pollinated taxa and their island endemic sister taxa showed that on average there was no overall reduction in flower size on islands, although specific lineages (e.g., Asteraceae, Solanaceae) and island groups (e.g., Galápagos, Revillagigedo Islands) did fit that expectation (Hetherington‐Rauth & Johnson, 2020). These results show that the evolution of reproductive traits such as flower size on islands is species‐specific and context‐dependent, making it difficult to generalize and highlighting the need for further research that investigates divergent evolution of reproductive traits between island and continental populations (Burns, 2019), which our study seeks to address.

*Tribulus cistoides* (L., Zygophyllaceae) is an excellent system to study the phenotypic variation of reproductive traits on islands in response to species interactions. *Tribulus cistoides* is found on many tropical islands and continents throughout the world. The spines of *T. cistoides* fruits lead to them being carried by larger animals and/or seabirds, which facilitate their arrival to islands, making them potentially native to many areas of the world (Hooker, 1847; Porter, 1971). In the same way, humans are also effective dispersers of *T. cistoides* and have helped the plant distribute throughout the world (Johnson et al., 2020). Classic expectations for the evolution of *Tribulus* antiherbivore defenses of their fruits are complex owing to the evolution of endemic granivores on some island archipelagos. With respect to mutualistic interactions in continental populations, *T. cistoides* are typically pollinated by a diversity of insects, including bees and butterflies (Huffaker et al., 1983). On islands, *T. cistoides* is mainly pollinated by an endemic community of pollinators. These attributes make *T. cistoides* well‐suited to study how the unique communities and environment of islands affect the phenotypic divergence of traits associated with antagonistic and mutualistic interactions (Carvajal‐Endara et al., 2020; Morrison & Scott, 1996; Rivkin et al., 2021; Scott & Morrison, 1996).

Here we investigate whether *T. cistoides* exhibits phenotypic divergence in traits associated with antagonistic and mutualistic interactions across continental and island habitats. Our main question was: How does insularity affect phenotypic divergence in plant reproductive traits that mediate species interactions with antagonist vertebrate granivores (i.e., mericarp size and number of spines) and mutualistic pollinators (i.e., flower size)? We expect that plant traits that mediate species interactions will diverge between island and continental populations due to differences in community interactions and/or divergent environmental conditions found on islands. Specifically, for antagonistic interactions, we expect that *T. cistoides* fruits to be larger and have more spines (i.e., better defended) on islands where vertebrate granivores are present, whereas on the continent there are mainly insect predators. For flowers that mediate mutualistic interactions with pollinators, we expect that island *T. cistoides* populations will evolve smaller flowers because islands generally have depauperate and generalized pollinator communities compared with the continent (Burns, 2019). Our study uses a combination of field‐collected samples and multiple herbaria samples to account for both fruit defensive traits and floral mutualistic traits. Fruit samples were collected from the field and from herbarium collections, and floral traits were collected exclusively from herbarium samples (Appendix S1; see Supplemental Data with this article). The inclusion of herbarium samples allowed us to test our expectations more broadly and to compare multiple continental and island populations throughout the world.

## 2 MATERIALS AND METHODS

### 2.1 Study system

**FIGURE 1:**
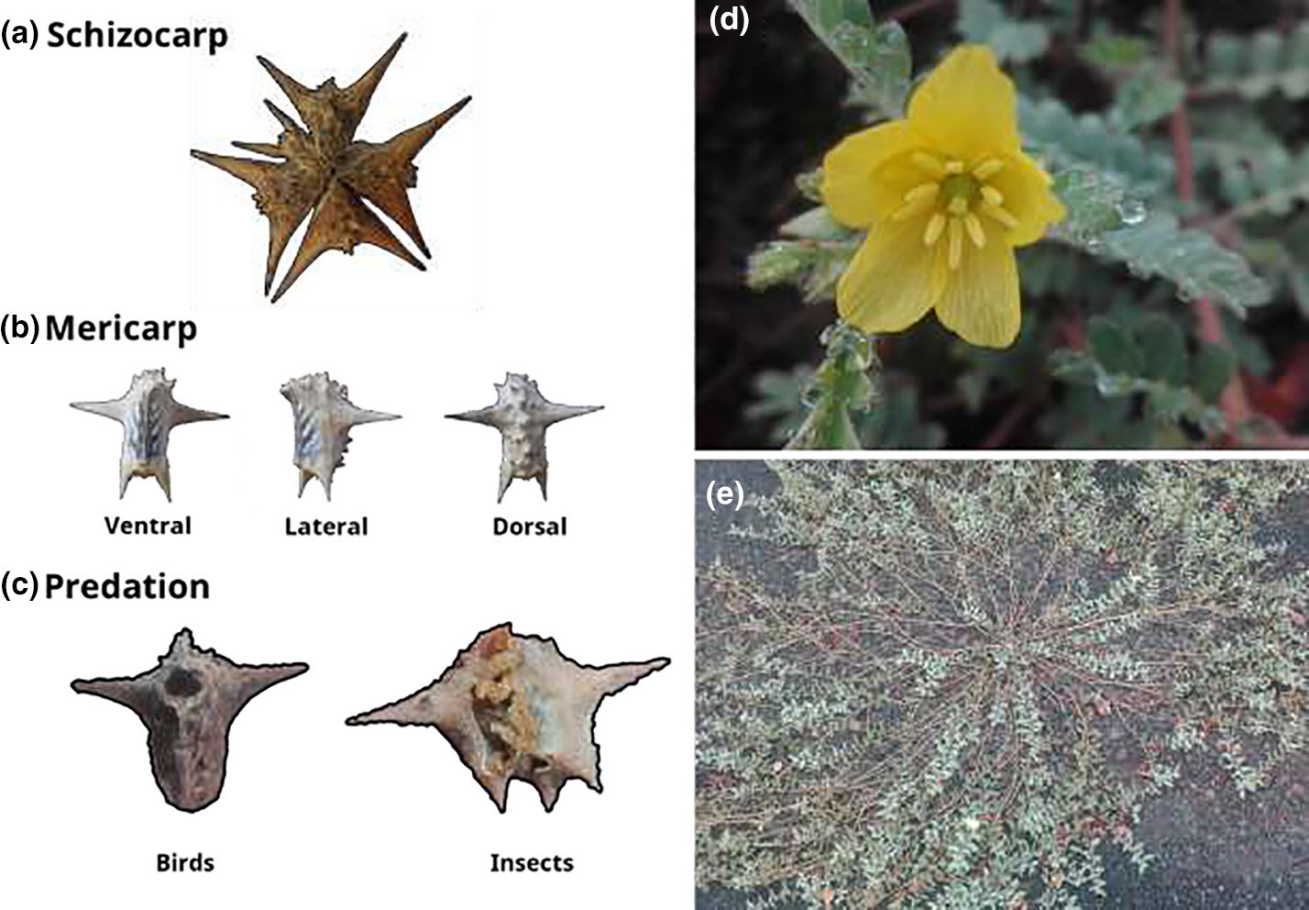
Morphology of *Tribulus cistoides* fruits and flowers. (a) A mature *T. cistoides* schizocarp, containing four developed mericarps plus one underdeveloped mericarp. (b) *T. cistoides* mericarps, showing their upper and lower spines. (c) Mericarp predation. The left mericarp was depredated by birds, showing the open gap that remains after seed removal. At the right is a mericarp being fed on by insect larvae. (d) Flower showing both male (anthers) and female (pistil) parts. (e) An individual *T. cistoides*, showing prostrate growth habit.

*Tribulus cistoides* is a perennial plant that is widely distributed in tropical and subtropical regions across the world (Porter, 1971, Appendix S2). Plants spread on the ground via long prostrate stems that radiate out from a central rootstock (Kearney et al., 2020). *Tribulus cistoides* has perfect flowers with five petals arranged in a radially symmetric pattern, measuring 20–40 mm in diameter (Porter, 1971; Wiggins & Porter, 1971). Petals have nectaries at their base, and although they can self‐pollinate, they are usually outcrossed by insect pollinators (Porter, 1971). Plants typically grow in well‐drained sandy or gravel soil on beaches, loose soil by field margins, roadsides or paths, and arid lowlands (Goeden & Ricker, 1973; Squires, 1979). *Tribulus cistoides* produce hard fibrous fruits called schizocarps, which have five individual segments called mericarps, each containing 1–7 seeds (Figure 1). As mericarps mature, they dry and fall adjacent to the plant. Mature mericarps can hold viable seeds for many years (Goeden & Ricker, 1973; Johnson, 1932). Mericarp changes are minimal once they fall from the plant, although spines tend to wear and break over time due to dispersal (Ernst & Tolsma, 1988; Scott & Morrison, 1996). Mericarps vary substantially in overall size, as well as the number and length of spines. Spine size and number can change due to selection for both dispersal (Johnson et al., 2020) and protection against avian granivores (Carvajal‐Endara et al., 2020; Figure 1).

Antagonistic interactions such as seed predation differ between continental and island populations of *Tribulus*. Insect predation is prominent on continental populations, where weevils (e.g., *Microlarinus lareynii, M. lypriformis* [Coleoptera: Curculonidae]) are used as a control agent to prevent *Tribulus terrestris* from spreading on cropland (Huffaker et al., 1983), and the weevil also attacks *T. cistoides* (Maddox, 1976; Stegmaier, 1973). Other studies report predation by cattle, although this is not intentional and potentially harms the animal (Johnson, 1932; Squires, 1979). Bird predation of *T. cistoides* seeds has been observed on Laysan Island in Hawaii (Conant, 1988) but is best known from the Galápagos islands (Carvajal‐Endara et al., 2020; Grant, 1981). Several species of ground finch (*Geospiza* spp.) feed on the seeds of *T. cistoides*, and their feeding behavior differs among species depending on their beak size. The largest beaked species, *Geospiza magnirostris* and *Geospiza conirostris*, crack mericarps more quickly than the medium ground finch *Geospiza fortis* (Grant, 1981). Being able to crack *T. cistoides* mericarps increases the survival of *G. fortis*, especially during dry years when preferred seeds of other species are depleted (Grant, 1981; Grant & Boag, 1980). Correspondingly, *T. cistoides* imposes selection on *G. fortis* beak size (Boag & Grant, 1981), which drives rapid adaptive evolution (Boag & Grant, 1981). Finch predation, in turn, imposes selection on *T. cistoides* mericarp morphology (Carvajal‐Endara et al., 2020). Mericarp size and spine number affect the probability of seed predation by finches. Specifically, the presence of lower spines (Figure 1) decreases predation in populations where *G. fortis* are present, but it does not affect predation by *G. magnirostris* (Carvajal‐Endara et al., 2020).

Mutualistic interactions, such as plant‐pollinator interactions, also differ for *Tribulus* between island and continental communities. In continental communities, *T. cistoides* interacts with a more diverse array of generalist and specialized insects, such as Hymenoptera (mainly various species of Apidae but also Scolidae), Diptera, Coloeptera, Lepidoptera, and Thysanoptera, to name a few groups (Austin, 1972; Reddi, 1981). On the Galápagos Islands, *T. cistoides* is considered a network hub for endemic and introduced pollinators alike (Traveset et al., 2013). Its most generalized pollinator is the endemic carpenter bee *Xylocopa darwinii (*Hymenoptera). Apart from another endemic, *Leptotes parrhasioides* (Lepidoptera), its other pollinators include introduced insects: a lycaenid, a wasp (Hymenoptera), and a hoverfly (Diptera; Traveset et al., 2013).

### 2.2 Data collection

#### 2.2.1 Mericarps

**FIGURE 2:**
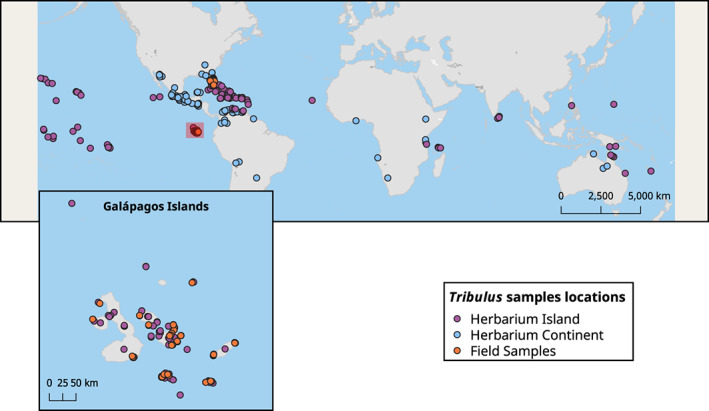
Distribution of samples of *Tribulus cistoides* collected for this study. Most samples around the world were collected from herbarium collections. Field samples collected by the authors are marked as orange circles including samples from Galápagos and Florida. In the large map, the Galápagos archipelago is outlined in red, with a blow‐up of the archipelago shown as an inset. The mericarp dataset was collected mainly from a combination of field‐collected samples and herbarium vouchers. The flower dataset was exclusively collected from herbarium samples. See Appendix S1 for details on sample numbers for each location.

Mericarps (*n* = 5084) were collected from field and herbarium samples. Field samples were collected from Galápagos and Florida. Herbarium samples were collected from the California Academy of Science (CAS), the Missouri Botanical Garden (MOBot), Harvard University Herbarium (HUH), and the Charles Darwin Research Station Herbarium (CDRS). Mericarp samples were collected between 1873 and 2018, and across 12 countries on three continents (Figure 2, see Appendix S1 for details on sample size). Linear mixed models were used to account for the unbalanced design as described below (see Section 2.3).

The morphology of mericarps was characterized by measuring five traits. These traits included mericarp length (mm), width (mm), depth (mm), spine tip distance (mm) (hereafter “spine size”), and the presence/absence of lower spines (see Section 3.1.1). These traits were included because they vary among mericarp populations (Appendix S3), and they have been shown to be subject to selection by Darwin's finches in past studies (Carvajal‐Endara et al., 2020; Grant, 1981; Rivkin et al., 2021). For herbarium mericarps, we only measured mericarps that had complete spines, and we did not measure mericarps that showed damage.

#### 2.2.2 Flowers

We characterized floral morphology from herbarium specimens. We obtained high‐resolution images of specimens (*n* = 772) from the Smithsonian Institute Herbarium, the Harvard University Herbarium (HUH), and the Charles Darwin Research Station Herbarium (CDRS). Collection dates ranged from 1800–2014 and included samples from 42 countries across five continents (Figure 2, see Appendix S1 for details on sample size). We focused on flower size quantified as the length of petals because flower size is a key trait influencing pollinator attraction, and this trait could be reliably measured from most flowering herbarium samples. Petal length (mm) was measured from the base to the tip of the petal, from up to three separate flowers per plant (see Section 3.1.2). All measurements were performed using ImageJ (Schneider et al., 2012).

#### 2.2.3 Bioclimatic data

We downloaded the data from the WorldClim database at a 30 s resolution (~1 km^2^) (Fick & Hijmans, 2017). We used four WorldClim variables: Bio1 (Annual Mean Temperature), Bio4 (Temperature seasonality), Bio12 (Annual precipitation), and Bio15 (Precipitation seasonality). The location coordinates and climate data were matched in QGIS (version 3.18.2‐Zürich; QGIS Development Team, 2022). We used the tool *Fill No Data* by a maximum distance of 10 pixels to project the climate information and reduce NAs from locations that may be too small to have estimated data. Then, we extracted the bioclimate information using the *Sampled Raster Values* tool and included the estimated data in our mericarp and flower datasets. In addition, we used the projected bioclimate estimates of Weigelt et al. (2013) for specific locations that we were unable to extract using the projected maps (Shungu‐Mbili island, Tanzania; Heron Island, Australia, the Kure, Pearl and Hermes Atolls, Hawaii; and the Lucayan Islands, Bahamas). However, for these locations, Weigelt did not estimate Bio4.

### 2.3 Statistical analyses

We used linear mixed‐effects models implemented in R version 4.0.3 (R Core Team, 2020). Our analytical approach involved the use of two models. Model 1 compared differences between populations located on continental versus island habitats. We used the definition of true oceanic islands mentioned by Whittaker and Fernández‐Palacios, as land surrounded by water (Whittaker & Fernandez‐Palacios, 2007). Model 2 focused on islands only and compared populations on the Galápagos versus other island systems. We used the *lmer* package (Bates et al., 2022) for the analysis of most traits, except for the presence of lower spines, which were fitted to binomial and negative binomial type II distributions, respectively, with a log link function implemented in the *glmmTMB* package (Brooks et al., 2017). Trait values typically varied among years, and so, the year of collection was included as a quantitative covariate.

We also included whether samples came from herbarium or field samples, to test any potential effect of shrinkage due to age, and sample ID was treated as a random effect to reflect the nonindependence of multiple measurements made per sample. Our full statistical model (Model 1) for testing the effects of islands versus continents on traits was: *trait ~ continental/island + year + herbarium + (1|ID)*. For flower size, we also contrasted the Galápagos islands versus other islands using the following model (Model 2): *petal length ~ Galápagos/other island + year + (1|ID)*. Model 2 did not include the herbarium covariate because all flower samples came from herbaria. We omitted model 2 in our mericarp dataset because we did not have enough samples from other islands to perform a robust analysis (Appendix S1). Sample ID allowed us to take multiple measurements from a single location, allowing us to accurately estimate the effects of each factor in the model without pseudoreplication, while accommodating the unbalanced sampling design inherent to using a mixture of field and herbarium samples. Sample ID referred to a single herbarium specimen or single field location for field samples. The year of collection was significant for some traits, and it allowed us to partition temporal trends in plant traits that may be associated with phenotypic change or collector bias. There was no significant difference between herbarium and field samples. For lower spines, the model differed, and we removed the effect of year: *lower spines ~ continental/island + herbarium + (1|ID)* because the model would not converge otherwise.

We used the *Anova* function from the *car* package (Fox et al., 2012) and fit the models to Type II sums‐of‐squares to test for the significance of fixed effects in the model, with marginal means estimated using the package *emmeans* (Lenth et al., 2022). We used the *Dharma* package (Hartig & Lohse, 2022) to assess whether residuals met assumptions of homogeneity of variance and normality in *lmer* models. Based on these diagnostics, we assessed whether the raw data or transformed data better‐fit model assumptions. For mericarp traits in Model 1, untransformed values of mericarp length and depth met model assumptions, whereas width was squareroot transformed. For spine size, 3.1% of mericarps (*n* = 158) lacked upper spines (Appendix S3) but only on the Galápagos. Even so, no difference was evident between continental and island populations for the presence/absence of upper spines (*χ* ^2^ = 0.7423, *p* = .3889), and so we removed all mericarps lacking upper spines from subsequent analyses of this trait. We further removed two large outliers (residuals>|9|) for spine size. For flower traits in Model 1, we filtered outliers (residuals >|5|). For Model 2, we squareroot transformed petal length to meet the assumptions of ANOVA.

We reran the models described above to include all bioclimatic variables as covariates in Model 1 and Model 2 to understand whether abiotic environmental variables helped to explain phenotypic divergence: *trait ~ continental/island + year + herbarium + Bio1 + Bio4 + Bio12 + Bio15 + (1|ID* *)* (Model 1); *petal length ~ Galápagos/other island + year + Bio1 + Bio4 + Bio12 + Bio15 + (1|ID* *)* (Model 2); and *lower spines ~ continental/island + herbarium + Bio1 + Bio4 + Bio12 + Bio15 + (1|ID)* for the presence of lower spines. We expected that the first set of analyses without bioclimatic variables would show whether there is an overall effect of island on phenotypic evolution. The second set of models that included bioclimatic variables, tested whether the climate of the island predicted the results instead of insularity per se (i.e., bioclimate variables were significant and continent/island became nonsignificant after being initially significant), or whether there was an effect of island independent of climate, which would indicate that insularity of plant–animal interactions itself influences evolution (island/continent is significant after including bioclimate variables).

Given our unequal replication between sampling locations, we considered three different approaches to further asses the robustness of our results for mericarps. First, we took the mean trait value from each sampling location and reran the analyses to test for divergence between island and continental populations. Second, we removed some individual herbarium vouchers that account for whole island systems to further reduce potential individual bias. We removed samples from two island systems, Cape Verde (*n* = 3) and Shungo‐Mbili Island (*n* = 5), and reran the analysis between island and continental populations. Finally, to assess the unbiased sampling effort from Galápagos, which accounts for most of our field‐collected samples (*n* = 3245). We removed Galápagos from the analysis and reran the models with only samples from other island systems. Then, we reran the analysis using only the Galápagos and continental samples to compare results. All these analyses showed similar effects and results to the original analyses and are presented in the supplements (Appendices S4–S7, respectively).

Finally, we used multivariate analysis to further explore how mericarp morphology differed between continental and island populations because mericarp length, width, depth, and spine size strongly covary (Appendix S8). First, we normally standardized each variable using the *scale* function in R and performed principal component analysis (PCA) using the *prcomp* function. We visualized the PCA using the *FactoExtra* package in R (Kassambara, 2017). Then, we extracted the scores from PC 1 and used the values to fit model 1 used for the univariate analysis above. We used the *Anova* function to test for the significance of the effect of habitat and bioclimate variables. We performed multivariate analysis for the additional analyses mentioned above when applicable (Appendices S4, S6, and S7).

## 3 RESULTS

### 3.1 Phenotypic divergence between island and continental habitats

#### 3.1.1 Mericarp morphology

**FIGURE 3:**
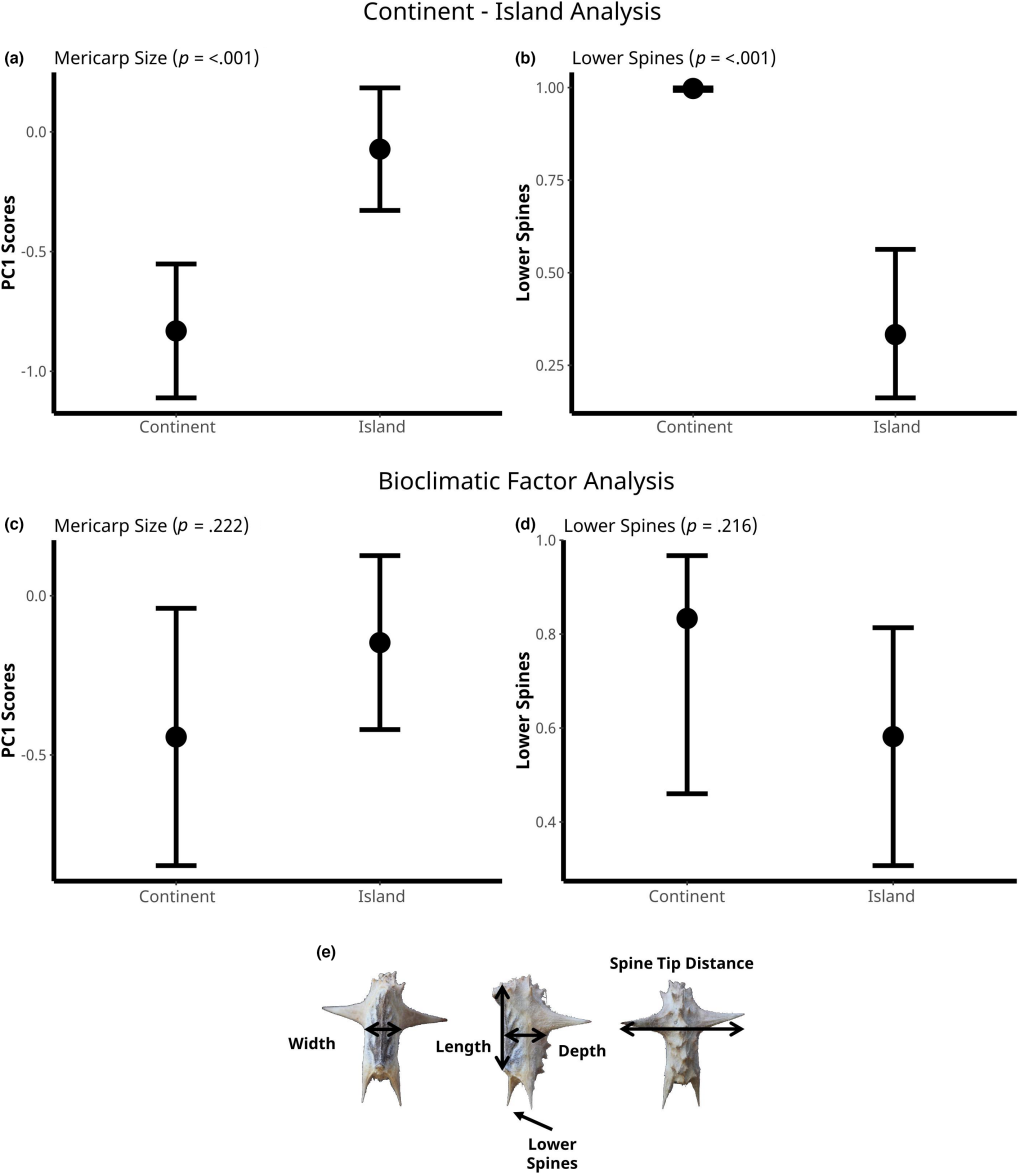
Mericarp traits compared between island and continental locations. Plots show the least‐squares mean estimates (±1 SE) using PC1 as a summary of mericarp size (length, width, depth, and spine size) and the presence or absence of lower spines. *p*‐values correspond to the difference between island and continental plants. (a, b) Estimates of continental and island populations only. (c, d) Estimates of the island effect from the model after accounting for bioclimatic variation. (e) Diagram of mericarp measurements: Length was measured along the ventral border of the mericarp where the seeds are contained within. Width was measured as the distance across the base of the upper spines. Depth was measured as the distance from the ventral and dorsal border in the middle of the mericarp. Spine size was the distance between the upper spine tips. Lower spines were considered present if they were longer than 1 mm and located at the base of the mericarp.

Mericarps phenotypically diverged between island and continental populations. Mericarps were on average 7% longer, 6% wider, and 12% deeper on islands compared with continental populations. Spine size was also 6% longer on islands (Table 1). At the same time, lower spines were 59% more common in continental populations than on islands (Figure 3). When we included bioclimatic variables in analyses, the effect of island/continent was qualitatively similar in the direction of effect but became nonsignificant for length (*p* = .388), width (*p* = .132), spine size (*p* = .393), and lower spines (*p* = .215), while it remained significant (*p* = .01) for mericarp depth. Bioclimatic variables explained variation in multiple traits: Bio4 (Temperature Seasonality) predicted variation in mericarp length and Bio15 (Annual precipitation) predicted mericarp depth (Table 2). All bioclimatic variables (Annual Mean Temperature, Temperature Seasonality, Annual precipitation, and Precipitation Seasonality) predicted variation in the presence/absence of lower spines (Table 2). These changes in the significance of the effect of islands imply that some of the divergence in mericarp traits is explained by variation in bioclimatic differences between islands and continents instead of the insularity of plant–animal interactions itself (Table 2).

**TABLE 1:** Model estimates of the effects of population and year of collection on mericarp and flower traits.

(a) Mericarp – Continental vs. island						
Trait	Continental/Island		Year		Field/Herbarium	
	*χ* ^2^	*p*	*χ* ^2^	*p*	*χ* ^2^	*p*
Length	**14.139**	**<.001**	**6.176**	**.012**	0.489	.484
Width	**12.047**	**<.001**	3.228	.072	0.012	.910
Depth	**51.506**	**<.001**	**11.107**	**<.001**	0.309	.578
Spine size	**5.850**	**.015**	0.731	.392	1.077	.299
Lower spines	**77.921**	**<.001**	—	—	3.254	.071
Mericarp Size (PC1)	**24.992**	**<.001**	**7.298**	**.006**	0.198	.655

(b) Flowers – Continental vs. island						
Trait	Continental/Island		Year		Field/Herbarium	
	*χ* ^2^	*p*	*χ* ^2^	*p*	*χ* ^2^	*p*
Petal length	1.386	.239	**15.623**	**<.001**	—	—

(c) Flowers – Galápagos vs. other islands						
Trait	Galápagos/Other islands		Year		Field/Herbarium	
	*χ* ^2^	*p*	*χ* ^2^	*p*	*χ* ^2^	*p*
Petal length	**157.147**	**<.001**	**9.453**	**.002**	—	—

*Note*: (a, b) Model 1 estimates from continental and island populations. (a) Individual mericarp traits and mericarp size (PC1). (b) Petal length. (c) Model 2 estimates from the effect of Galápagos and other non‐Galápagos island populations on petal length.

Bold values indicate statistically significant results at *p* < 0.05.

**TABLE 2:** Model estimates of the effects of population, year of collection, and bioclimatic variables on mericarp and flower traits.

(a) Mericarp – Continental vs. island														
Trait	Continental/Island		Year		Field/Herbarium		Bio1		Bio4		Bio12		Bio15	
	*χ* ^2^	*p*	*χ* ^2^	*p*	*χ* ^2^	*p*	*χ* ^2^	*p*	*χ* ^2^	*p*	*χ* ^2^	*p*	*χ* ^2^	*p*
Length	0.664	.414	**5.646**	**.017**	0.006	.937	0.067	.794	**4.373**	**.036**	2.252	.133	3.097	.078
Width	2.202	.137	2.829	.092	0.028	.865	0.214	.643	0.845	.357	0.077	.780	2.547	.110
Depth	**5.669**	**.017**	**11.402**	**<.001**	0.586	.443	0.132	.716	0.119	.729	0.466	.494	**6.879**	**.008**
Spine size	0.597	.439	0.597	.408	0.495	.481	0.563	.453	0.553	.456	1.562	.211	0.049	.824
Lower spines	1.530	.216	—	—	0.001	.970	**8.605**	**.003**	**10.701**	**.001**	**19.497**	**<.001**	**18.157**	**<.001**
Mericarp Size (PC1)	1.487	.222	**7.384**	**.006**	0.067	.795	0.042	.836	1.941	.163	2.345	.125	2.934	.086

(b) Flowers – Continental vs. island														
Trait	Continental/Island		Year		Field/Herbarium		Bio1		Bio4		Bio12		Bio15	
	*χ* ^2^	*p*	*χ* ^2^	*p*	*χ* ^2^	*p*	*χ* ^2^	*p*	*χ* ^2^	*p*	*χ* ^2^	*p*	*χ* ^2^	*p*
Petal length	**10.043**	**.001**	**15.950**	**<.001**	—	—	**13.657**	**<.001**	**15.299**	**<.001**	2.167	0.140	**18.533**	**<.001**

(c) Flowers – Galápagos vs. other islands														
Trait	Galápagos/Other islands		Year		Field/Herbarium		Bio1		Bio4		Bio12		Bio15	
	*χ* ^2^	*p*	*χ* ^2^	*p*	*χ* ^2^	*p*	*χ* ^2^	*p*	*χ* ^2^	*p*	*χ* ^2^	*p*	*χ* ^2^	*p*
Petal length	**84.867**	**<.001**	**5.188**	**.022**	—	—	1.909	.167	0.471	.492	2.622	.105	0.522	.470

*Note*: Nomenclature on bioclimate variables was taken from the WorldClim dataset (https://worldclim.org/). We used variables Bio1 (Annual Mean Temperature), Bio4 (Temperature Seasonality), Bio12 (Annual precipitation), Bio15 (Precipitation Seasonality). (a, b) Model 1 estimates from continental and island populations. (a) Individual mericarp traits and mericarp size (PC1). (b) Petal length. (c) Model 2 estimates the effect of Galápagos and other non‐Galápagos island populations on petal length.

Bold values indicate statistically significant results at *p* < 0.05).

Underlined values are close to statistical significance (i.e. 0.05 < *p* < 0.10).

Our additional analysis showed the same trend. There was a general effect of increased mericarp size that was lost after accounting for environmental factors, which explained the observed variation (Appendices S6 and S7). However, we found that lower spines were not significant when we removed the Galápagos from the analysis (*p* = .246; Appendix S6, Table S9).

**FIGURE 4:**
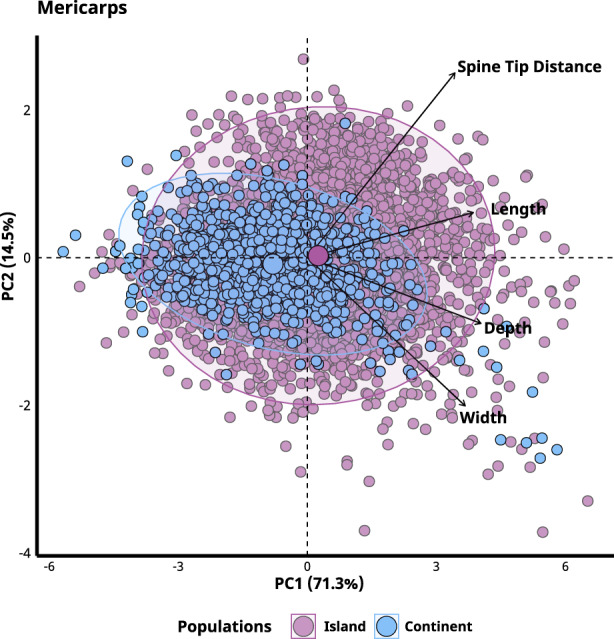
Principal component analysis of mericarp traits. Points represent all individual mericarps sampled. Vectors are proportional to the contribution and direction associated with each trait. Groups are separated into island and continental populations. Larger circles represent the centroid of the ellipses with a 95% confidence interval. Although individual mericarps are shown here, statistical tests between island/continental sites were based on scores along PC1 fit to a GLMM using Model 1, which accounted for nonindependence of mericarps from the same sampling location.

Multivariate analysis explained 86% of the variation in mericarp morphology and further supported the univariate analyses, showing that mericarps differed between continental and island populations but also became nonsignificant when bioclimatic variables were added (Figure 3; Table 2). PC_1_ explained 71% of the variance in mericarp morphology and was mostly associated with mericarp size (length, depth, width), and PC_2_ explained 15% of the variance and was mainly associated with spine size (Figure 4).

#### 3.1.2 Flower size

**FIGURE 5:**
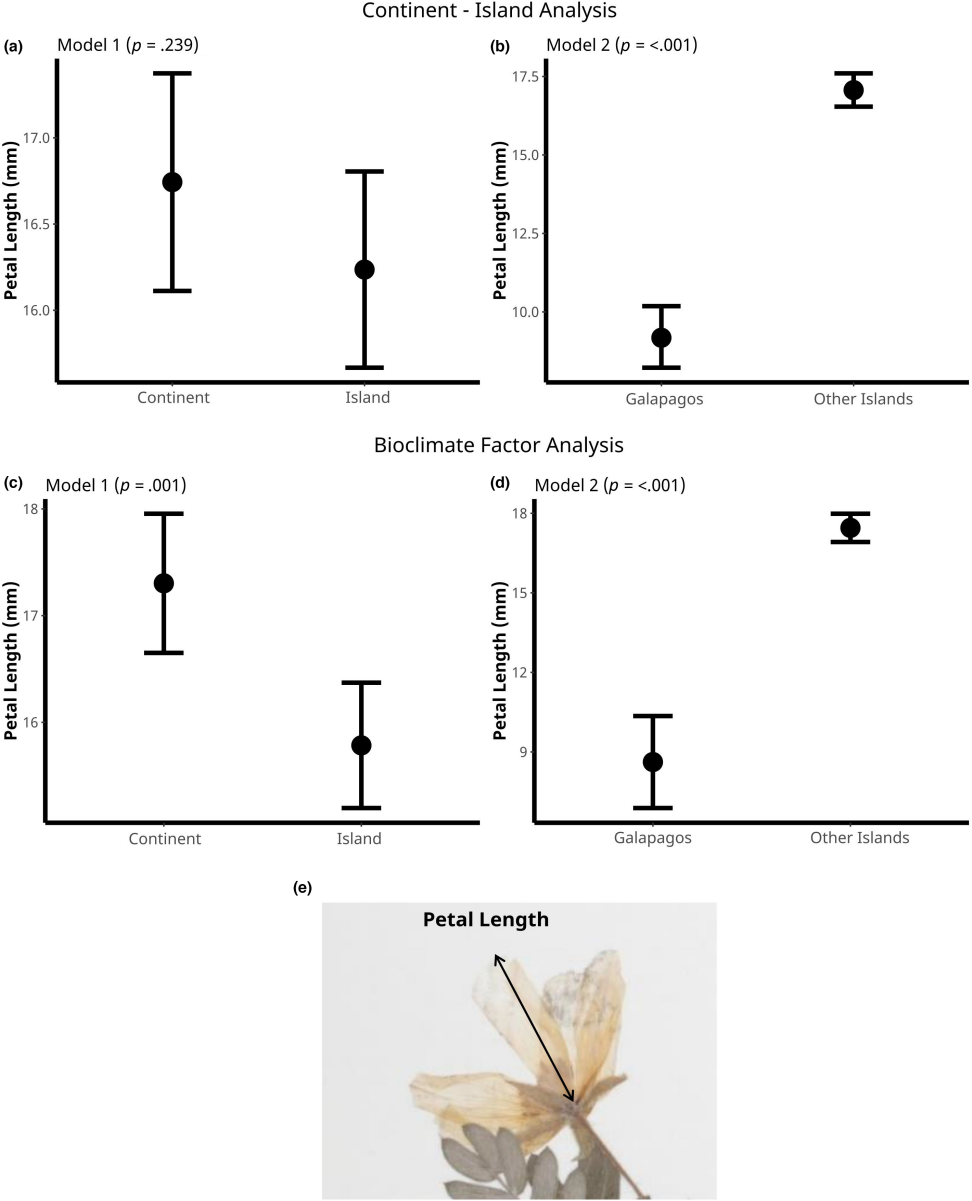
Petal length estimates from island and continental plants. The plots show the least‐squares mean estimates (±1 SE) using petal length. *p*‐values correspond to the difference between island and continental plants (Model 1), and the difference between the Galápagos Islands and Other (non‐Galápagos) islands (Model 2). (a) Estimates of continental and island populations only. (b) Estimates of Galápagos and other (non‐Galápagos) islands only. (c, d) Estimates of the island effect from the models after accounting for bioclimatic variation. (e) Diagram of how petal length was measured: from the base to the tip of the petal.

Flower size differed between island and continental habitats, but these effects were only apparent after accounting for bioclimatic variation among sample sites (Figure 5; Table 2). When we fit Model 1 there was no clear effect of island/continent (*p* = .239, Table 1), but when we included bioclimatic variables, the effect of island/continent became highly significant (*p* = .001, Table 2), with petals on the continent being on average 9% longer than petals on islands. Bio1 (Annual Mean Temperature), Bio4 (Temperature Seasonality), and Bio15 (Precipitation Seasonality) all predicted variations in petal size (Table 2). This result shows that abiotic factors have a large impact on the divergence of flower size among sampling locations, and island/continent divergence in flower size is only apparent after accounting for this effect.

Our additional analysis showed that the insularity effect becomes nonsignificant when we remove the Galápagos samples and only use Other Islands (*p* = .118; Appendix S6, Table S9). But we found the same bioclimatic variables predicted variation in flower size (Appendix S6, Table S10).

### 3.2 Phenotypic divergence between the Galápagos islands and other island groups

#### 3.2.1 Flower size

We found that *T. cistoides* flowers on the Galápagos were smaller than on other islands. Specifically, the petal length of *T. cistoides* was 46% shorter on the Galápagos than on other islands (Figure 5 ). This effect was apparent whether bioclimatic variables were included or not, with no bioclimatic variables significantly predicting variation in flower size when only island sites were included in analyses (Table 2).

## 4 DISCUSSION

We found that fruit and floral traits that mediate antagonistic and mutualistic species interactions with *T. cistoides* frequently diverged between island and continental populations. Mericarps were larger and deeper on islands but more frequently lacked lower spines in comparison to continental populations. After accounting for climatic variation, the divergence in all mericarp traits except depth became nonsignificant, while climatic variables frequently predicted variation in mericarp morphology. By contrast, flower size consistently diverged to be smaller on island than continental populations, particularly after accounting for bioclimatic variation among sampling sites. Plants on the Galápagos islands had substantially smaller flowers than plants from other islands. We discuss the importance of these results for understanding how insularity influences the evolution of traits associated with species interactions.

### 4.1 Divergence of antagonistic traits between islands and continent

The morphological divergence observed between island and continental populations is partially consistent with our expectations of evolution in response to changes in herbivore communities. We expected that mericarps would be larger and better defended on islands if vertebrate seed predation was an important and unique agent of selection on islands (Boag & Grant, 1981; Carvajal‐Endara et al., 2020). We found that mericarp depth was still significantly different between island and continental plants after the inclusion of bioclimate variables. Increased mericarp depth may increase the survival of mericarps when vertebrate predators are present. In the case of ground finches on the Galápagos, the birds crack the mericarps transversely, twisting the lower surface of the mericarp, such that a deeper mericarp may increase handling time for finches (Grant, 1999). Mericarp size is especially important for finches with medium‐sized beaks because it takes them more time to handle large mericarps when extracting seeds. By contrast, the less numerous large beaked finches open larger mericarps more easily to extract seeds (Grant, 1999). Previous field experiments showed that on average the ground finches on the Galápagos imposed phenotypic selection in favor of larger *Tribulus* mericarps, and longer upper spines (Carvajal‐Endara et al., 2020; Rivkin et al., 2021).

We expected that if mericarps were better defended on islands, then lower spines would be more frequent there than in continental plants. For example, the presence of lower spines on the Galápagos increases survival from vertebrate predators (Carvajal‐Endara et al., 2020). In general, we found that lower spines were less common on islands. However, the difference in the presence/absence of spines between island and continental populations disappeared when the Galápagos samples were removed (Appendices S6 and S7), indicating that the loss of spines was mainly a phenonemon restricted to the Galápagos. Moreover, bioclimatic variation was the best explanation for variation in the presence/absence of lower spines. This may have occurred because precipitation and seasonality drive increased seed production of a diversity of plant species on islands. The abundance of alternative seed sources alleviates predation and antagonistic selection on defense traits of *T. cistoides*, which is a nonpreferred food source when other more easily acquired seeds are available (Carvajal‐Endara et al., 2020; Grant, 1999; Grant & Boag, 1980). The effects of seasonal climatic variation in predation could facilitate the maintenance of variation in traits like lower spines.

Another explanation for the decreased frequency of lower spines on islands could be differences in dispersal between islands and continents (Cody, 2006; Cody & Overton, 1996). Upper and lower spines of *T. cistoides* are involved in dispersal and defense, in that the fruits become attached to animals (Johnson et al., 2020; Porter, 1971; Wiggins & Porter, 1971). These spines might be an especially important mechanism for dispersal in continental populations but could be disadvantageous on islands, especially if dispersal disproportionately leads to seeds being deposited in unfavorable habitats. As suggested by Porter (1971), seabirds may carry *T. cistoides* mericarps, potentially depositing them in the ocean. Alternatively, larger seeds may help seedling establishment while islands may lack dispersal agents, leading to higher costs of maintaining lower spines without substantial benefits (Burns, 2019; Kavanagh & Burns, 2014). If the cost: benefit ratio of maintaining spines is high on islands, then larger seeds could explain both why fruits tend to be larger and lower spines are less frequent on islands.

Conflicting selection due to antagonistic and mutualistic interactions on fruits may frequently lead to phenotypic divergence among populations. For example, Siepielski and Benkman (2010) found that seed predation by squirrels led to the selection of pine cones to be more defended and contain fewer seeds. In the absence of squirrels, seed dispersal by nutcrackers selected for pinecones to have lower investment in defenses and larger seeds. When both agents of selection were present, it led to the contrasting selection and greater phenotypic variation within populations. Notably, we also observed greater variation in morphological traits on islands than on continents (Figure 4). This type of opposing selection by antagonistic and mutualistic interactions may similarly explain why fleshy fruits that rely on seed dispersers often have spines (e.g., *Ribes* spp., *Durio* spp.), and why many types of fruits are chemically defended (e.g., *Solanum* spp., *Hippomane mancinella*). These contrasting traits may allow plants to attract beneficial dispersers and deter costly predators. It seems likely that the evolution of many plants' fruit and seed traits reflects a balance of conflicting selection between antagonistic and mutualistic interactions (Blake et al., 2012; Jordano, 1995; O'Farrill et al., 2013; Stiles, 1980).

### 4.2 Divergence of mutualistic traits between islands and continents

We expected that flowers on islands would be consistently smaller because islands commonly have lower diversity and more generalized pollinators. This expectation is founded on the Island Floral Syndrome Hypothesis (Hetherington‐Rauth & Johnson, 2020), which stems from observations made by naturalists during the past two centuries (Bramwell & Caujapé‐Castells, 2011; Carlquist, 1974; Darwin, 1845; Wallace, 2013). These naturalists claimed that islands typically have small inconspicuous flowers. Our results differ from this expectation, in that there was no consistent difference in petal length between island and continent populations (Appendix S6). However, our results are consistent with this expectation after accounting for climatic variation among sampling sites, and on the Galápagos archipelago especially, where flowers were about half the size of flowers than on other islands or continental populations.

Our results for other island systems are supported by a recent large comparative analysis across the Pacific Islands. Hetherington‐Rauth & Johnson (2020) found that, across many taxa, flowers were not on average smaller on islands than on the American continents. Interestingly, Galápagos was a notable exception in their study, where endemic species' flowers were consistently smaller on the archipelago compared to their continental sister taxa. *Tribulus cistoides* was not used in that study, and so it is striking that our results align with their previous macroevolutionary results for other species on the same archipelago. This correspondence raises the question: why is the Galápagos an exception and why do we observe the evolution of smaller flowers both within and between species?

In the case of *T. cistoides* in Galápagos, changes in flower size could be explained by the evolution of increased selfing, divergence in pollinator communities, or climatic differences. *Tribulus cistoides* is self‐compatible (Chamorro et al., 2012), but seed production is thought to rely mostly on outcrossing mediated by pollinators (Reddi, 1981). It is conceivable that island populations of *T. cistoides* are evolving increased selfing rates and consequently smaller flower sizes. This possibility deserves further investigation. Divergent pollinator communities on islands may also contribute to the evolution of smaller flowers. For example, there is only a single native bee species found on the Galápagos, the large endemic carpenter bee *Xylocopa darwinii*. The paucity of bee species in the Galápagos could lead to high competition among plant species for attracting pollinators, which might drive the evolution of smaller flower sizes in *T. cistoides* to attract different pollinators, such as smaller Hymenoptera, day‐flying butterflies, or even nocturnal moths, many of which are introduced. Introduced species comprise ~25% of all Galápagos insect species (Traveset et al., 2013). If introduced pollinators are sustained by nonendemic or generalist plants, they may impose selection for smaller flowers. Finally, it was clear from our results that environmental variation among populations explained differences in flower size. Specifically, there was a significant effect of temperature and precipitation seasonality for island and continental populations. It is possible that environmental factors are the main source of selection for flowers, or that they modulate the strength and direction of biotic selection, as suggested by the differences in flower size between islands and continents that were only apparent after accounting for climate.

An alternative explanation to the possibilities above is that the smaller flowers of *T. cistoides* are not an example of adaptive evolution to a depauperate pollinator community but instead reflect recent hybridization. The Galápagos is just one of two locations in the world where the larger flowered *T. cistoides* and the smaller flowered *T. terrestris* coexist. If these species have hybridized on the Galápagos, then the observation of their smaller flowers could reflect segregating hybrid variation. Porter (1971) reported that diagnostic floral characteristics of these two species were intermediate and variable among *T. cistoides* plants on the Galápagos. Our own data show that mericarps on islands exhibit greater variation in morphology than on the continent (Figure 5), as expected if there is hybrid segregating variation on Galápagos but not elsewhere. These observations are not conclusive evidence of hybridization, and genomic analyses would be required to further test whether hybridization is occurring on the islands and accounting for increased phenotypic variance.

### 4.3 Limitations

Our study has two main limitations that need to be considered when interpreting our results. First, our mericarp dataset was unbalanced, in that we had an abundance of mericarp data from the Galápagos islands, yet relatively few mericarp samples from other island systems. Few herbarium specimens containing mericarps were available from other islands and visiting many additional islands across *T. cistoides*' global distribution was logistically infeasible. As such, most of our mericarp field samples came from the Galápagos and continental Florida, whereas all other continental and island samples distributed around the world came from herbarium specimens. Thus, our results that compare continental and Galápagos populations are robust, but our ability to compare morphological patterns of mericarps on the Galápagos versus other islands is limited. Second, our results reflect phenotypic differences among field samples, yet we cannot partition the effects of genetic versus environmental differences. Given our large replication and the magnitude of the differences we observed, plus previous results showing evidence consistent with heritable variation in some of these traits (Carvajal‐Endara et al., 2020), it is reasonable to conclude that much of this variation is genetically based.

## 5 CONCLUSIONS AND FUTURE DIRECTIONS

Our results show that *T. cistoides* exhibits phenotypic differences in fruit and floral traits between island and continental habitats. Many of these differences are consistent with antagonistic and mutualistic interactions driving divergent evolution between continental and insular populations, while in other cases climatic variation appears to be the main driver, or at least modulates biotic selection. This study shows the potential of using a species that is globally distributed and shows unique interactions in the context of island populations. These characteristics make *Tribulus* a potential species for multiple avenues of research answering questions on island evolution and ecology. The global distribution of *T. cistoides* and the inclusion of herbarium samples is an asset for pursuing large‐scale comparative studies in the tropics. For island systems specifically, there is potential for controlled experiments to address specific dynamics of vertebrate and invertebrate predation on islands. Observed phenotypic divergence on mericarps or flowers could be further explored with common gardens that would allow for the partitioning of genetic and plastic differences in phenotypic traits (i.e., presence of lower spines) observed within and among populations. Molecular analyses would help establish whether hybridization on islands is contributing to the observed phenotypic variation in plant traits of *T. cistoides*. Furthermore, field experiments of pollinators and dispersers could help to establish how selection by mutualists is shaping the evolution of mericarp and floral traits. Overall, our study shows the potential for using *Tribulus* as a study system to understand co‐evolutionary dynamics driven by mutualistic and antagonistic interactions.

## AUTHOR CONTRIBUTIONS

**Winer Daniel Reyes‐Corral:** Data curation (equal); formal analysis (equal); investigation (equal); methodology (equal); writing – original draft (equal); writing – review and editing (equal). **Sofia Carvajal‐Endara:** Data curation (equal); investigation (equal); writing – review and editing (equal). **Molly Hetherington‐Rauth:** Data curation (equal); writing – review and editing (equal). **Jaime A. Chaves:** Funding acquisition (equal); investigation (equal); writing – review and editing (equal). **Peter R. Grant:** Conceptualization (equal); investigation (equal); writing – review and editing (equal). **B. Rosemary Grant:** Conceptualization (equal); investigation (equal); writing – review and editing (equal). **Andrew P. Hendry:** Funding acquisition (equal); supervision (equal); validation (equal); writing – review and editing (equal). **Marc T. J. Johnson:** Conceptualization (equal); data curation (equal); formal analysis (equal); funding acquisition (equal); supervision (equal); validation (equal); writing – original draft (equal); writing – review and editing (equal).

### OPEN RESEARCH BADGES

This article has earned an Open Data badge for making publicly available the digitally‐shareable data necessary to reproduce the reported results. The data is available at [https://doi.org/10.5061/dryad.h70rxwdnz; https://github.com/Winer‐DanielR/Tribulus‐mericarp‐morphology].

## Supporting information


Data S1.

## Data Availability

Data are available at DRYAD: https://doi.org/10.5061/dryad.h70rxwdnz. Scripts are available at GitHub at: https://github.com/Winer‐DanielR/Tribulus‐mericarp‐morphology.

## References

[ece39766-bib-0001] Austin, D. F. (1972). Interactions between *Apis mellifera* (Hymenoptera: Apidae) and *Tribulus cistoides* (Zygophyllaceae). Rhodora, 74, 117–123.

[ece39766-bib-0002] Baier, F. , & Hoekstra, H. E. (2019). The genetics of morphological and behavioural Island traits in deer mice. Proceedings of the Royal Society B: Biological Sciences, 286, 20191697. 10.1098/rspb.2019.1697 PMC6842855 31662081

[ece39766-bib-0003] Barrett, S. C. H. (1996). The reproductive biology and genetics of Island plants. Philosophical Transactions of the Royal Society of London. Series B, Biological Sciences, 351, 725–733.

[ece39766-bib-0004] Bates, D. , Maechler, M. , Bolker, B. , Walker, S. , Christensen, R. H. B. , Singmann, H. , Dai, B. , Scheipl, F. , & Grothendieck, G. (2022). lme4: Linear mixed‐effects models using ‘Eigen’ and S4.

[ece39766-bib-0005] Blake, S. , Wikelski, M. , Cabrera, F. , Guezou, A. , Silva, M. , Sadeghayobi, E. , Yackulic, C. B. , & Jaramillo, P. (2012). Seed dispersal by Galápagos tortoises. Journal of Biogeography, 39, 1961–1972.

[ece39766-bib-0006] Boag, P. T. , & Grant, P. R. (1981). Intense natural selection in a population of Darwin's finches (Geospizinae) in the Galápagos. Science, 214, 82–85. 17802577 10.1126/science.214.4516.82

[ece39766-bib-0007] Bowen, L. , & Vuren, D. V. (1997). Insular endemic plants lack defenses against herbivores. Conservation Biology, 11, 1249–1254.

[ece39766-bib-0008] Bramwell, D. , & Caujapé‐Castells, J. Eds. (2011). The biology of Island floras. Cambridge University Press.

[ece39766-bib-0009] Brooks, M. E. , Kristensen, K. , van Benthem, J. K. , Magnusson, A. , Berg, C. W. , Nielsen, A. , Skaug, H. J. , Mächler, M. , & Bolker, B. M. (2017). glmmTMB balances speed and flexibility among packages for zero‐inflated generalized linear mixed modeling. The R Journal, 9, 378–400.

[ece39766-bib-0010] Burns, K. C. (2019). Evolution in isolation: The search for an Island syndrome in plants. Cambridge University Press.

[ece39766-bib-0011] Carlquist, S. J. (1965). Island life: A natural history of the islands of the world (1st ed.). Published for the American Museum of Natural History [by] The Natural History Press.

[ece39766-bib-0012] Carlquist, S. J. (1974). Island biology. Columbia University Press.

[ece39766-bib-0013] Carvajal‐Endara, S. , Hendry, A. P. , Emery, N. C. , Neu, C. P. , Carmona, D. , Gotanda, K. M. , Davies, T. J. , Chaves, J. A. , & Johnson, M. T. J. (2020). The ecology and evolution of seed predation by Darwin's finches on *Tribulus cistoides* on the Galápagos Islands. Ecological Monographs, 90, e01392.

[ece39766-bib-0014] Chamorro, S. , Heleno, R. , Olesen, J. M. , McMullen, C. K. , & Traveset, A. (2012). Pollination patterns and plant breeding systems in the Galápagos: A review. Annals of Botany, 110, 1489–1501. 22691541 10.1093/aob/mcs132 PMC3489146

[ece39766-bib-0015] Cody, M. L. (2006). Plants on islands: Diversity and dynamics on a continental archipelago. University of California Press.

[ece39766-bib-0016] Cody, M. L. , & Overton, J. M. (1996). Short‐term evolution of reduced dispersal in Island plant populations. The Journal of Ecology, 84, 53.

[ece39766-bib-0017] Conant, S. (1988). Geographic variation in the Laysan finch (*Telespyza cantans*). Evolutionary Ecology, 2, 270–282.

[ece39766-bib-0018] Cummins, G. C. , Theimer, T. C. , & Paxton, E. H. (2020). Responses to terrestrial nest predators by endemic and introduced Hawaiian birds. Ecology and Evolution, 10, 1949–1958. 32128128 10.1002/ece3.6021 PMC7042753

[ece39766-bib-0019] Darwin, C. (1845). Journal of researches into the natural history and geology of the countries visited during the voyage of H.M.S. beagle round the world. John Murray.

[ece39766-bib-0020] Ernst, W. H. O. , & Tolsma, D. J. (1988). Dormancy and germination of semi‐arid annual plant species, *Tragus berteronianus* and *Tribulus terrestris* . Flora, 181, 243–251.

[ece39766-bib-0021] Fick, S. E. , & Hijmans, R. J. (2017). WorldClim 2: New 1‐km spatial resolution climate surfaces for global land areas. International Journal of Climatology, 37, 4302–4315.

[ece39766-bib-0022] Fox, J. , Weisberg, S. , Adler, D. , Bates, D. , Baud‐Bovy, G. , Ellison, S. , Firth, D. , Friendly, M. , Gorjanc, G. , Graves, S. , & Heiberger, R. (2012). Package ‘car’ (p. 16). R Foundation for Statistical Computing.

[ece39766-bib-0024] Goeden, R. D. , & Ricker, D. W. (1973). A soil profile analysis for puncturevine fruit and seed. Weed Science, 21, 504–507.

[ece39766-bib-0025] Grant, P. R. (1981). The feeding of Darwin's finches on *Tribulus cistoides* (L.) seeds. Animal Behaviour, 29, 785–793.

[ece39766-bib-0026] Grant, P. R. (1998). Evolution on islands: Originating from contributions to a discussion meeting of the Royal Society of London. Oxford University Press.

[ece39766-bib-0027] Grant, P. R. (1999). Ecology and evolution of Darwin's finches. Princeton University Press.

[ece39766-bib-0028] Grant, P. R. , & Boag, P. T. (1980). Rainfall on the Galápagos and the demography of Darwin's finches. The Auk, 97, 227–244.

[ece39766-bib-0029] Hartig, F. , & Lohse, L. (2022). DHARMa: Residual diagnostics for hierarchical (multi‐level/mixed) regression models. University of Regensburg.

[ece39766-bib-0030] Hetherington‐Rauth, M. C. , & Johnson, M. T. J. (2020). Floral trait evolution of angiosperms on Pacific Islands. The American Naturalist, 196, 87–100. 10.1086/709018 32552102

[ece39766-bib-0031] Hoan, R. P. , Ormond, R. A. , & Barton, K. E. (2014). Prickly poppies can get pricklier: Ontogenetic patterns in the induction of physical defense traits. PLoS One, 9, e96796. 24802133 10.1371/journal.pone.0096796 PMC4011880

[ece39766-bib-0032] Hooker, J. D. (1847). IX. An enumeration of the plants of the Galapagos archipelago; with descriptions of those which are new. Transactions of the Linnean Society of London, 20, 163–233.

[ece39766-bib-0033] Huffaker, C. B. , Hamai, J. , & Nowierski, R. M. (1983). Biological control of puncturevine, *Tribulus terrestris* in California after twenty years of activity of introduced weevils. Entomophaga, 28, 387–400.

[ece39766-bib-0034] Inoue, K. , & Amano, M. (1986). Evolution of *Campanula punctata* Lam. in the Izu Islands: Changes of pollinators and evolution of breeding systems. Plant Species Biology, 1, 89–97.

[ece39766-bib-0035] Janzen, D. H. (1973). Dissolution of mutualism between *Cecropia* and its *Azteca* ants. Biotropica, 5, 15–28.

[ece39766-bib-0036] Johnson, E. (1932). The puncture vine in California. Agricultural Experiment Station.

[ece39766-bib-0037] Johnson, M. K. A. , Johnson, O. P. J. , Johnson, R. A. , & Johnson, M. T. J. (2020). The role of spines in anthropogenic seed dispersal on the Galápagos Islands. Ecology and Evolution, 10, 1639–1647. 32076540 10.1002/ece3.6020 PMC7029089

[ece39766-bib-0038] Jordano, P. (1995). Frugivore‐mediated selection on fruit and seed size – Birds and St Lucies cherry, *Prunus mahaleb* . Ecology, 76, 2627–2639.

[ece39766-bib-0039] Kavanagh, P. H. , & Burns, K. C. (2014). The repeated evolution of large seeds on islands. Proceedings of the Royal Society B: Biological Sciences, 281, 20140675. 10.1098/rspb.2014.0675 PMC4046418 24850930

[ece39766-bib-0040] Kearney, T. H. , Peebles, R. H. , Howell, H. T. , & McClintock, E. (2020). Arizona Flora. 2nd ed. identifies 3,438 species of flowering plants, ferns, and Fern‐allies growing uncultivated in Arizona, reprint 2020. University of California Press.

[ece39766-bib-0041] Lenth, R. V. , Buerkner, P. , Herve, M. , Love, J. , Miguez, F. , Riebl, H. , & Singmann, H. (2022). Emmeans: Estimated marginal means, aka least‐squares means.

[ece39766-bib-0043] Losos, J. B. , & Ricklefs, R. E. (2009). Adaptation and diversification on islands. Nature, 457, 830–836. 19212401 10.1038/nature07893

[ece39766-bib-0044] Maddox, D. M. (1976). History of weevils on puncturevine in and near the United States. Weed Science, 24, 414–419.

[ece39766-bib-0045] Martén‐Rodríguez, S. , Quesada, M. , Castro, A.‐A. , Lopezaraiza‐Mikel, M. , & Fenster, C. B. (2015). A comparison of reproductive strategies between Island and mainland Caribbean Gesneriaceae. Journal of Ecology, 103, 1190–1204.

[ece39766-bib-0046] Meredith, F. L. , Tindall, M. L. , Hemmings, F. A. , & Moles, A. T. (2019). Prickly pairs: The proportion of spinescent species does not differ between islands and mainlands. Journal of Plant Ecology, 12, 941–948.

[ece39766-bib-0047] Monroy, P. , & García‐Verdugo, C. (2019). Testing the hypothesis of loss of defenses on islands across a wide latitudinal gradient of *Periploca laevigata* populations. American Journal of Botany, 106, 303–312. 30742704 10.1002/ajb2.1232

[ece39766-bib-0048] Moreira, X. , Castagneyrol, B. , García‐Verdugo, C. , & Abdala‐Roberts, L. (2021). A meta‐analysis of insularity effects on herbivory and plant defences. Journal of Biogeography, 48, 386–393.

[ece39766-bib-0049] Morrison, S. , & Scott, J. (1996). Variation in populations of *Tribulus terrestris* (Zygophyllaceae). 2: Chromosome numbers. Australian Journal of Botany, 44, 191.

[ece39766-bib-0050] O'Farrill, G. , Galetti, M. , & Campos‐Arceiz, A. (2013). Frugivory and seed dispersal by tapirs: An insight on their ecological role. Integrative Zoology, 8, 4–17. 23586556 10.1111/j.1749-4877.2012.00316.x

[ece39766-bib-0052] Porter, D. M. (1971). Notes on the floral glands in *Tribulus* (Zygophyllaceae). Annals of the Missouri Botanical Garden, 58, 1.

[ece39766-bib-0053] QGIS Development Team . (2022). QGIS geographic information system. https://www.qgis.org/en/site/

[ece39766-bib-0054] R Core Team . (2020). R: A language and environment for statistical computing. R Core Team.

[ece39766-bib-0055] Reddi, C. S. (1981). Breeding structure and pollination ecology of *Tribulus terrestris* . Proceedings of the Indian National Science Academy, B47(2), 1854930981.

[ece39766-bib-0056] Rivkin, L. R. , Johnson, R. A. , Chaves, J. A. , & Johnson, M. T. J. (2021). Urbanization alters interactions between Darwin's finches and *Tribulus cistoides* on the Galápagos Islands. Ecology and Evolution, 11, 15754–15765. 34824787 10.1002/ece3.8236 PMC8601916

[ece39766-bib-0057] Schneider, C. A. , Rasband, W. S. , & Eliceiri, K. W. (2012). NIH image to ImageJ: 25 years of image analysis. Nature Methods, 9, 671–675. 22930834 10.1038/nmeth.2089 PMC5554542

[ece39766-bib-0058] Scott, J. , & Morrison, S. (1996). Variation in populations of *Tribulus terrestris* (Zygophyllaceae).1: Burr morphology. Australian Journal of Botany, 44, 175.

[ece39766-bib-0059] Siepielski, A. M. , & Benkman, C. W. (2010). Conflicting selection from an antagonist and a mutualist enhances phenotypic variation in a plant. Evolution, 64, 1120–1128. 19817846 10.1111/j.1558-5646.2009.00867.x

[ece39766-bib-0060] Squires, V. R. (1979). The biology of Australian weeds. 1. *Tribulus terrestris* L. Journal of the Australian Institute of Agricultural Science, 45, 75–82.

[ece39766-bib-0061] Stegmaier, C. E. (1973). Colonization of the puncturevine stem weevil, *Microlarinus lypriformis* (Coleoptera: Curculionidae) with notes on parasitism in South Florida. The Florida Entomologist, 56, 235–241.

[ece39766-bib-0062] Stiles, E. W. (1980). Patterns of fruit presentation and seed dispersal in bird‐disseminated woody plants in the eastern deciduous Forest. The American Naturalist, 116, 670–688.

[ece39766-bib-0063] Traveset, A. , Heleno, R. , Chamorro, S. , Vargas, P. , McMullen, C. K. , Castro‐Urgal, R. , Nogales, M. , Herrera, H. W. , & Olesen, J. M. (2013). Invaders of pollination networks in the Galápagos Islands: Emergence of novel communities. Proceedings of the Royal Society B: Biological Sciences, 280, 20123040. 10.1098/rspb.2012.3040 PMC3619457 23486435

[ece39766-bib-0064] Traveset, A. , & Navarro, L. (2018). Plant reproductive ecology and evolution in the Mediterranean islands: State of the art. Plant Biology, 20, 63–77. 28945322 10.1111/plb.12636

[ece39766-bib-0065] Wallace, A. R. (2013). Tropical nature and other essays. Cambridge University Press.

[ece39766-bib-0066] Weigelt, P. , Jetz, W. , & Kreft, H. (2013). Bioclimatic and physical characterization of the world's islands. Proceedings of the National Academy of Sciences of the United States of America, 110, 15307–15312. 24003123 10.1073/pnas.1306309110 PMC3780862

[ece39766-bib-0067] Whittaker, R. J. , & Fernandez‐Palacios, J. M. (2007). Island biogeography: Ecology, evolution, and conservation (p. 75). Oxford University Press.

[ece39766-bib-0068] Wiggins, I. L. , & Porter, D. M. (1971). Flora of the Galápagos Islands. Stanford University Press.

[ece39766-bib-0069] Yamada, T. , Kashiwagi, T. , Sawamura, M. , & Maki, M. (2010). Floral differentiation among insular and mainland populations of weigela coraeensis (Caprifoliaceae). Plant Systematics and Evolution, 288, 113–125.

