# Supplementary material for: Phenotypic divergence of traits that mediate antagonistic and mutualistic interactions between island and continental populations of the tropical plant, Tribulus cistoides (Zygophyllaceae)
Source: Ecol Evol. 2023 Mar 22;13(3):e9766. doi: 10.1002/ece3.9766 (PMC10031297; doi:10.1002/ece3.9766)
Supplement: Supplementary file 1 — Data S1. [file ECE3-13-e9766-s001.docx]

**appendix s1**

**TABLE S1** Sample distribution of mericarp and flower datasets. The table shows the distribution of samples from island and continents and their source, from herbarium or field samples.

| Sets | Continents | Population | Source | n | Total |
| --- | --- | --- | --- | --- | --- |
| Mericarps | Africa | Island | Herbarium | 8 | 47 |
|  |  |  | Field samples | - |  |
|  |  | Continent | Herbarium | 39 |  |
|  |  |  | Field samples | - |  |
|  | North and Central America | Island | Herbarium | 86 | 1291 |
|  |  |  | Field samples | 200 |  |
|  |  | Continent | Herbarium | 81 |  |
|  |  |  | Field samples | 924 |  |
|  | South America | Island | Herbarium | 505 | 3829 |
|  |  |  | Field samples | 3245 |  |
|  |  | Continent | Herbarium | 79 |  |
|  |  |  | Field samples | - |  |
| Flowers | Africa | Island | Herbarium | 6 | 26 |
|  |  |  | Field samples | - |  |
|  |  | Continent | Herbarium | 20 |  |
|  |  |  | Field samples | - |  |
|  | Asia | Island | Herbarium | 22 | 22 |
|  |  |  | Field samples | - |  |
|  |  | Continent | Herbarium | - |  |
|  |  |  | Field samples | - |  |
|  | North America and Caribbean | Island | Herbarium | 260 | 512 |
|  |  |  | Field samples | - |  |
|  |  | Continent | Herbarium | 252 |  |
|  |  |  | Field samples | - |  |
|  | Oceania | Island | Herbarium | 52 | 58 |
|  |  |  | Field samples | - |  |
|  |  | Continent | Herbarium | 6 |  |
|  |  |  | Field samples | - |  |
|  | South America | Island | Herbarium | 69 | 154 |
|  |  |  | Field samples | - |  |
|  |  | Continent | Herbarium | 85 |  |
|  |  |  | Field samples | - |  |

**TABLE S2** Distribution of mericarp and flower samples from Galápagos and Other Islands.

| Sets | Islands | Continent | Island Location | n | Total |
| --- | --- | --- | --- | --- | --- |
| Mericarps | **Galápagos** | South America | Baltra | 193 | 3745 |
|  |  |  | Champion | 51 |  |
|  |  |  | Daphne Major | 88 |  |
|  |  |  | Daphne Minor | 42 |  |
|  |  |  | Darwin | 2 |  |
|  |  |  | Enderby | 6 |  |
|  |  |  | Espanola | 264 |  |
|  |  |  | Fernandina | 73 |  |
|  |  |  | Floreana | 605 |  |
|  |  |  | Genovesa | 29 |  |
|  |  |  | Guy Fawkes West | 5 |  |
|  |  |  | Isabela | 671 |  |
|  |  |  | Pinta | 12 |  |
|  |  |  | Plaza Norte | 2 |  |
|  |  |  | Rabida | 124 |  |
|  |  |  | San Cristobal | 405 |  |
|  |  |  | Santa Cruz | 1005 |  |
|  |  |  | Santiago | 55 |  |
|  |  |  | Seymour Norte | 113 |  |
|  | **Oher Islands** | Africa | Cape Verde Islands | 3 | 8 |
|  |  |  | Shungu-Mbili Island | 5 |  |
|  |  | North and Central America | Boca Grande | 100 | 286 |
|  |  |  | Clarion Island | 7 |  |
|  |  |  | India Key | 15 |  |
|  |  |  | Key Biscayne, FL | 4 |  |
|  |  |  | Key West, FL | 20 |  |
|  |  |  | Marathon | 100 |  |
|  |  |  | Big Coppett Key | 5 |  |
|  |  |  | Socorro Island | 35 |  |
|  |  | South America | Isla de Salamanca | 5 | 5 |
| Flowers | **Galápagos** | South America | Baltra | 5 | 48 |
|  |  |  | Champion | 2 |  |
|  |  |  | Daphne Major | 4 |  |
|  |  |  | Darwin | 2 |  |
|  |  |  | Eden | 1 |  |
|  |  |  | Fernandina | 1 |  |
|  |  |  | Floreana | 1 |  |
|  |  |  | Gardner | 2 |  |
|  |  |  | Isabela | 15 |  |
|  |  |  | Plaza Norte | 1 |  |
|  |  |  | Plaza Sur | 3 |  |
|  |  |  | Santa Cruz | 7 |  |
|  |  |  | Santiago | 4 |  |
|  | **Other Islands** | Africa | Seychelles Islands | 3 | 6 |
|  |  |  | Cape Verde Islands | 1 |  |
|  |  |  | Zanzibar | 2 |  |
|  |  | Asia | Philippines | 4 | 22 |
|  |  |  | Sri Lanka | 18 |  |
|  |  | North America and Caribbean | Antigua and Barbuda | 5 | 252 |
|  |  |  | Bahamas | 18 |  |
|  |  |  | British Virgin Islands | 2 |  |
|  |  |  | Clarion Island | 6 |  |
|  |  |  | Cuba | 17 |  |
|  |  |  | Dominican Republic | 22 |  |
|  |  |  | Guadeloupe | 6 |  |
|  |  |  | Haiti | 30 |  |
|  |  |  | Jamaica | 49 |  |
|  |  |  | Martinique | 1 |  |
|  |  |  | The Revillagigedo Islands | 8 |  |
|  |  |  | Socorro Island | 9 |  |
|  |  |  | Puerto Rico | 6 |  |
|  |  |  | Turks and Caicos Islands (Lucayan Archipelago) | 13 |  |
|  |  |  | U.S. Virgin Islands | 8 |  |
|  |  |  | Florida Keys | 23 |  |
|  |  |  | Hawaiian Islands | 29 |  |

**appendix s2**


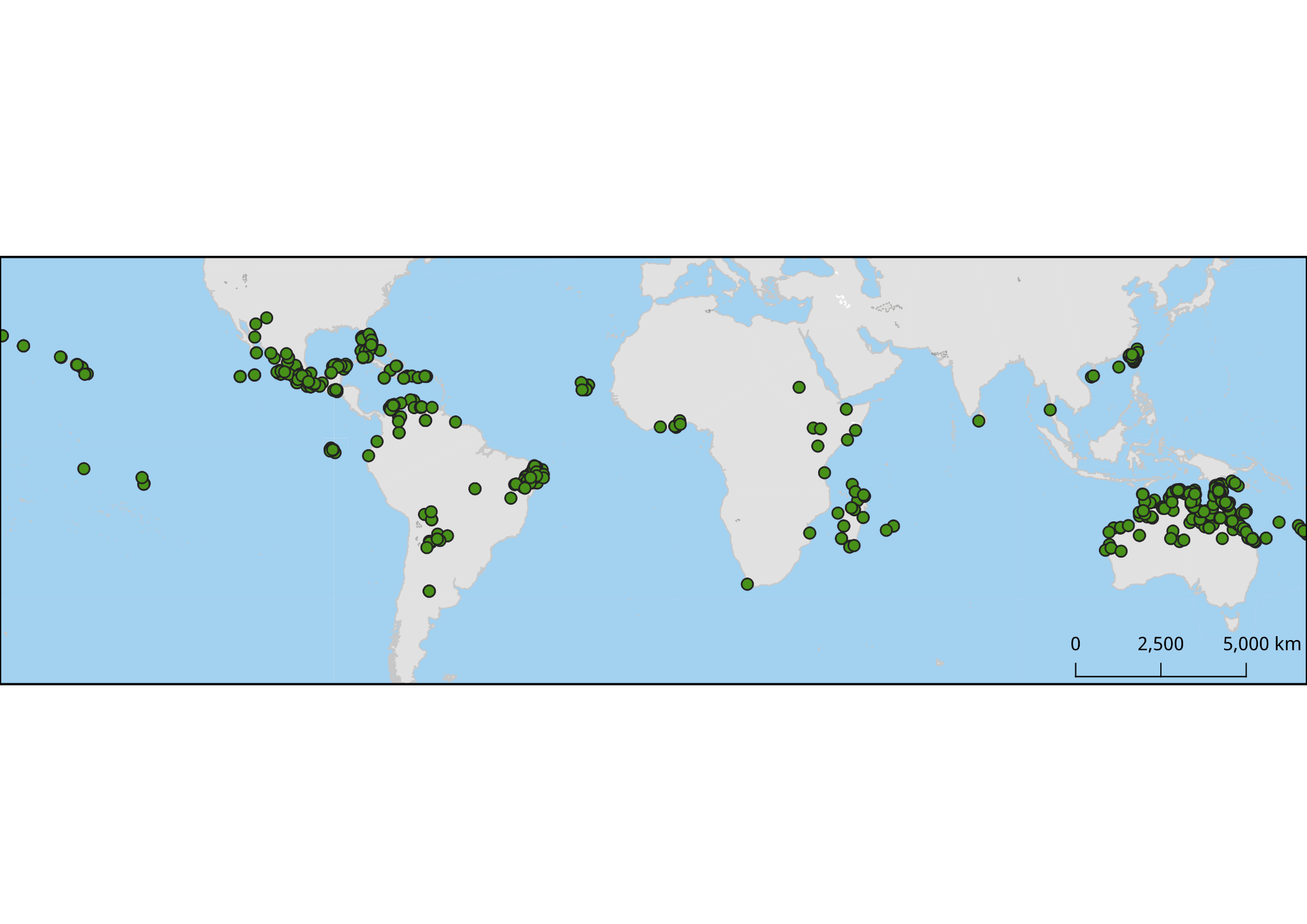


**Figure S1**

Global distribution of *Tribulus cistoides* based on specimens from the Global Biodiversity Information Facility (GBIF) (n = 1213). The collection ranges from 1770 - 2021.

**appendix s3**


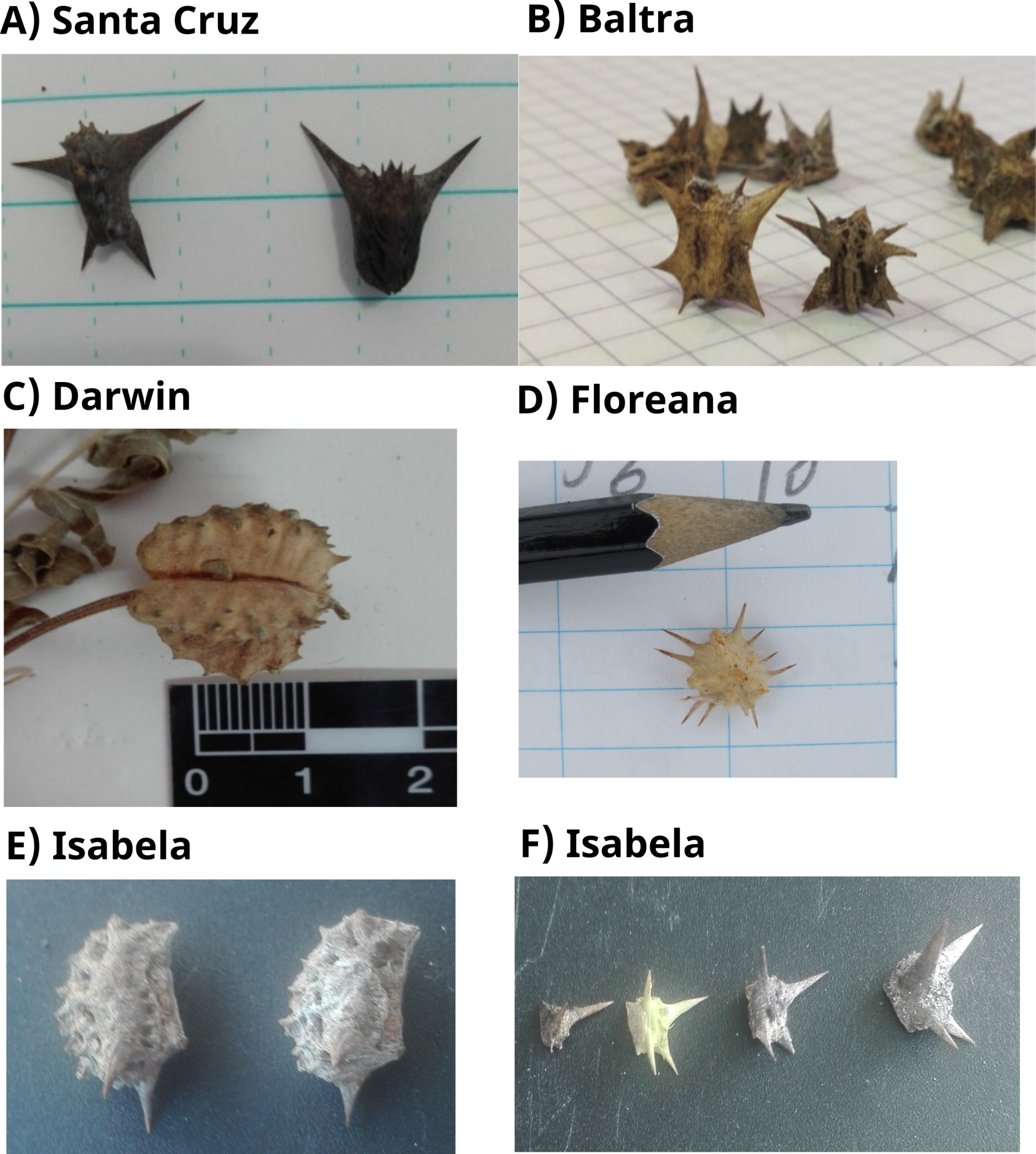


**Figure S2**

Mericarp phenotypic variation found on the Galápagos Islands. Mericarps shown are taken from A) Santa Cruz, B) Baltra, C) Darwin, D) Floreana, and E-F) Isabela islands. Mericarps on the Galápagos differ in size, shape, and spine number. Some phenotypes lack lower or upper spines. These phenotypes coexist and individual plants produce the same phenotype. Although the frequency of mericarps with no spines is less common, they can be found in populations that are close to towns and roads. Photos by WDRC.

**appendix s4**

**Table S3**

Model estimates of the effect of population and year of collection on mean mericarp traits. The table shows the model estimates per trait and the mean PC1 estimates. The means were from Galápagos and Florida populations to help account for unbalanced sampling. Other locations were kept as individual observations. Estimating the means of these two locations gave a total of 561 observations. Means were calculated using ID, that identifies the populations or locations of the samples.

| **Mericarp Means – *continental vs island*** | | | | | | | |
| --- | --- | --- | --- | --- | --- | --- | --- |
|  | **Trait** | **Continental/Island** | | **Year** | | **Field/Herbarium** | |
|  |  | **χ^2^** | **P** | **χ^2^** | **P** | **χ^2^** | **P** |
|  | Length | **15.2892** | **<0.001** | **13.6705** | **<0.001** | 1.6815 | 0.19472 |
|  | Width | **9.0171** | **0.00268** | 2.2999 | 0.12938 | 0.0061 | 0.938 |
|  | Depth | **33.5116** | **<0.001** | **9.9451** | **0.00161** | 0.1914 | 0.66178 |
|  | Spine size | 0.578 | 0.44724 | 3.6738 | 0.05528 | 1.3526 | 0.24483 |
|  | Mericarp Size (PC1) | **12.453** | **<0.001** | 10.6016 | 0.001 | 0.6904 | 0.4060 |

**TABLE S4**

Model estimates of the effect of population and year of collection on mean mericarp traits including bioclimate variables. The table shows the model estimates per trait and the mean PC1 estimates. The means were from Galápagos and Florida populations to account for unbalanced sampling. Nomenclature on bioclimate variables was taken from the WorldClim dataset (<https://worldclim.org/>). We used variables Bio1 (Annual Mean Temperature), Bio4 (Temperature Seasonality), Bio12 (Annual precipitation), Bio15 (Precipitation Seasonality).

| **Mericarp Means – *continental vs island*** | | | | | | | | | | | | | | | |
| --- | --- | --- | --- | --- | --- | --- | --- | --- | --- | --- | --- | --- | --- | --- | --- |
|  | **Trait** | **Continental/Island** | | **Year** | | **Field/Herbarium** | | **Bio1** | | **Bio4** | | **Bio12** | | **Bio15** | |
|  |  | **χ^2^** | **P** | **χ^2^** | **P** | **χ^2^** | **P** | **χ^2^** | **P** | **χ^2^** | **P** | **χ^2^** | **P** | **χ^2^** | **P** |
|  | Length | 3.533 | 0.0602 | **10.601** | **0.0011** | 0.028 | 0.8668 | 0.023 | 0.8793 | **6.485** | **0.0108** | 1.016 | 0.3134 | 2.245 | 0.1340 |
|  | Width | 2.766 | 0.0963 | 1.242 | 0.2651 | 0.528 | 0.4673 | 0.287 | 0.5916 | 2.684 | 0.1013 | 0.017 | 0.894 | **5.078** | **0.024** |
|  | Depth | **5.962** | **0.0146** | **8.796** | **<0.001** | 0.031 | 0.8601 | 0.374 | 0.5406 | 1.411 | 0.2347 | 0.395 | 0.5296 | **6.592** | **0.0102** |
|  | Spine size | 0.167 | 0.6824 | 2.731 | 0.0984 | 0.200 | 0.6544 | 0.807 | 0.3689 | 1.536 | 0.2151 | 0.023 | 0.8781 | 1.064 | 0.3023 |
|  | Mericarp Size (PC1) | 1.432 | 0.2314 | **8.574** | **0.0034** | 0 | 0.9972 | 0.077 | 0.7812 | **4.735** | **0.0295** | 1.162 | 0.2809 | **3.892** | **0.0485** |


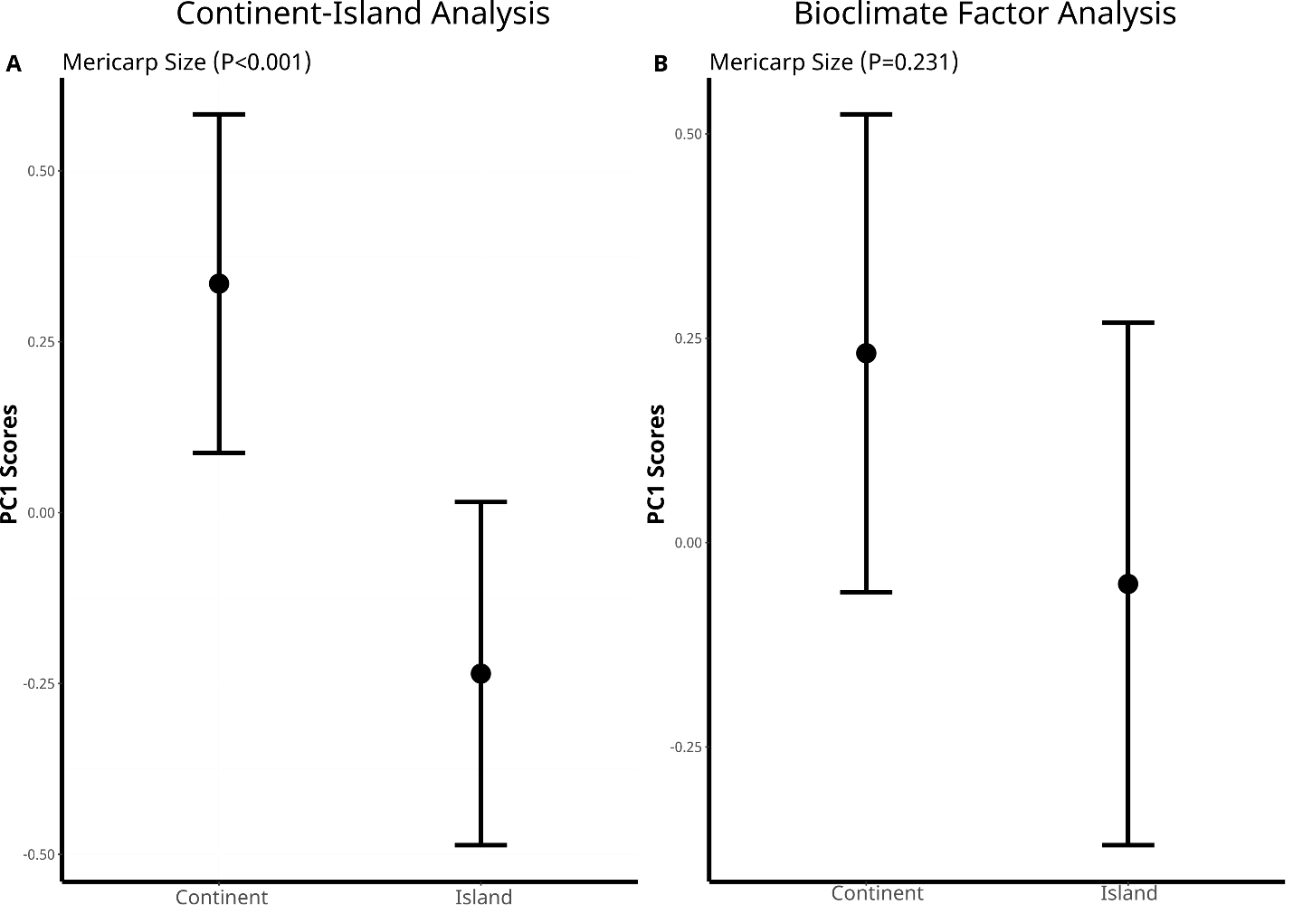


**Figure S3**

Mean mericarp traits compared between island and continental locations. Means were estimated to account for unbalanced sampling from Galápagos and Florida (n = 3829, n = 1291). Locations were grouped by ID. Plots are the least-squares means ± one standard error. On top of each plot, it shows the p-values from the ANOVA. A) Mean mericarp trait plots of continent and island populations only. B) Mean mericarp trait plots including bioclimate variables.


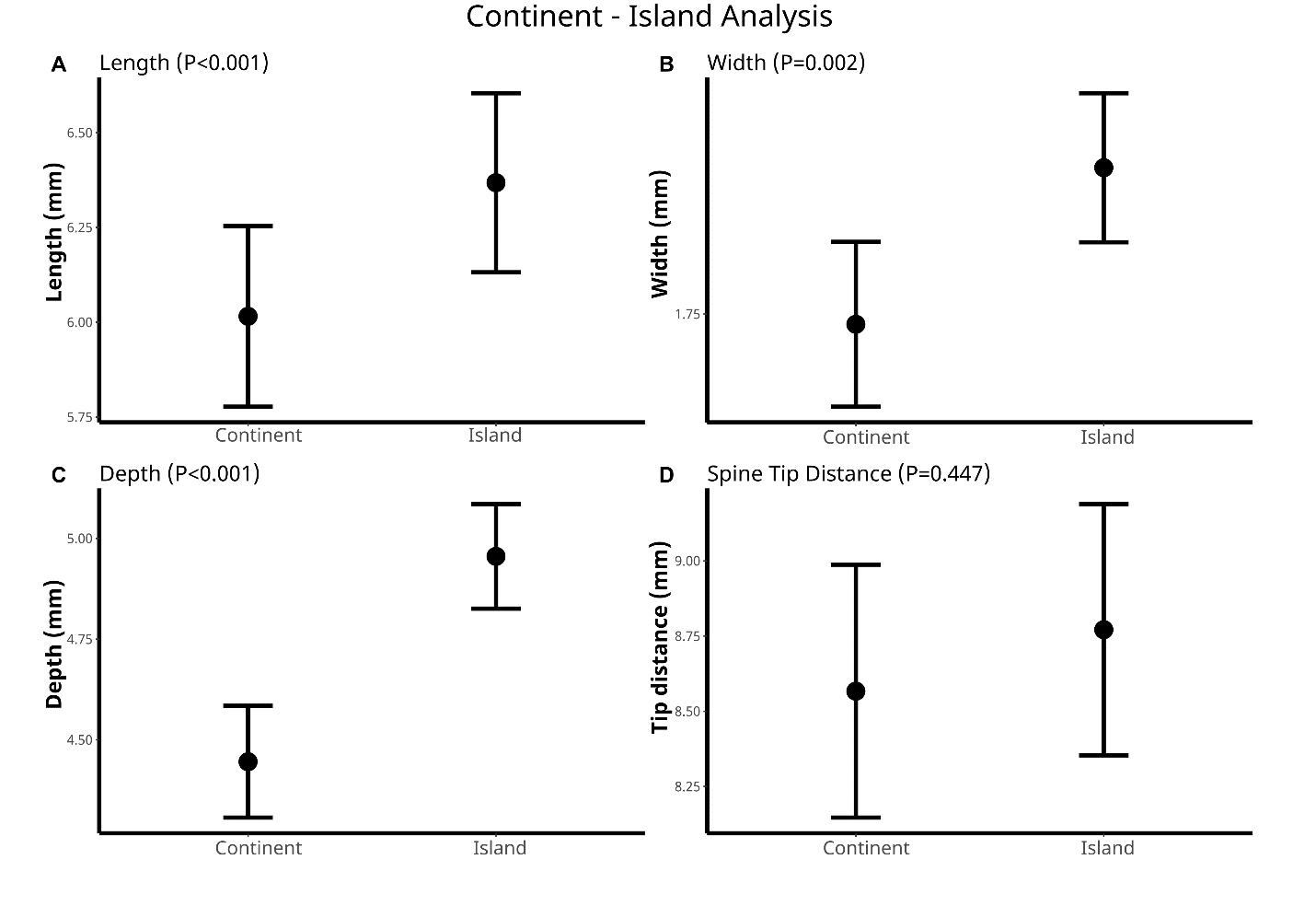


**FIGURE S4**

Mean individual mericarp traits compared between island and continental locations. Means were estimated to account for unbalanced sampling from Galápagos and Florida (n = 3829, n = 1291). Locations were grouped by ID. Plots are the least-squares means ± one standard error. On top of each plot, it shows the p-values from the ANOVA. A-D) Mericarp trait plots without bioclimatic variables included.


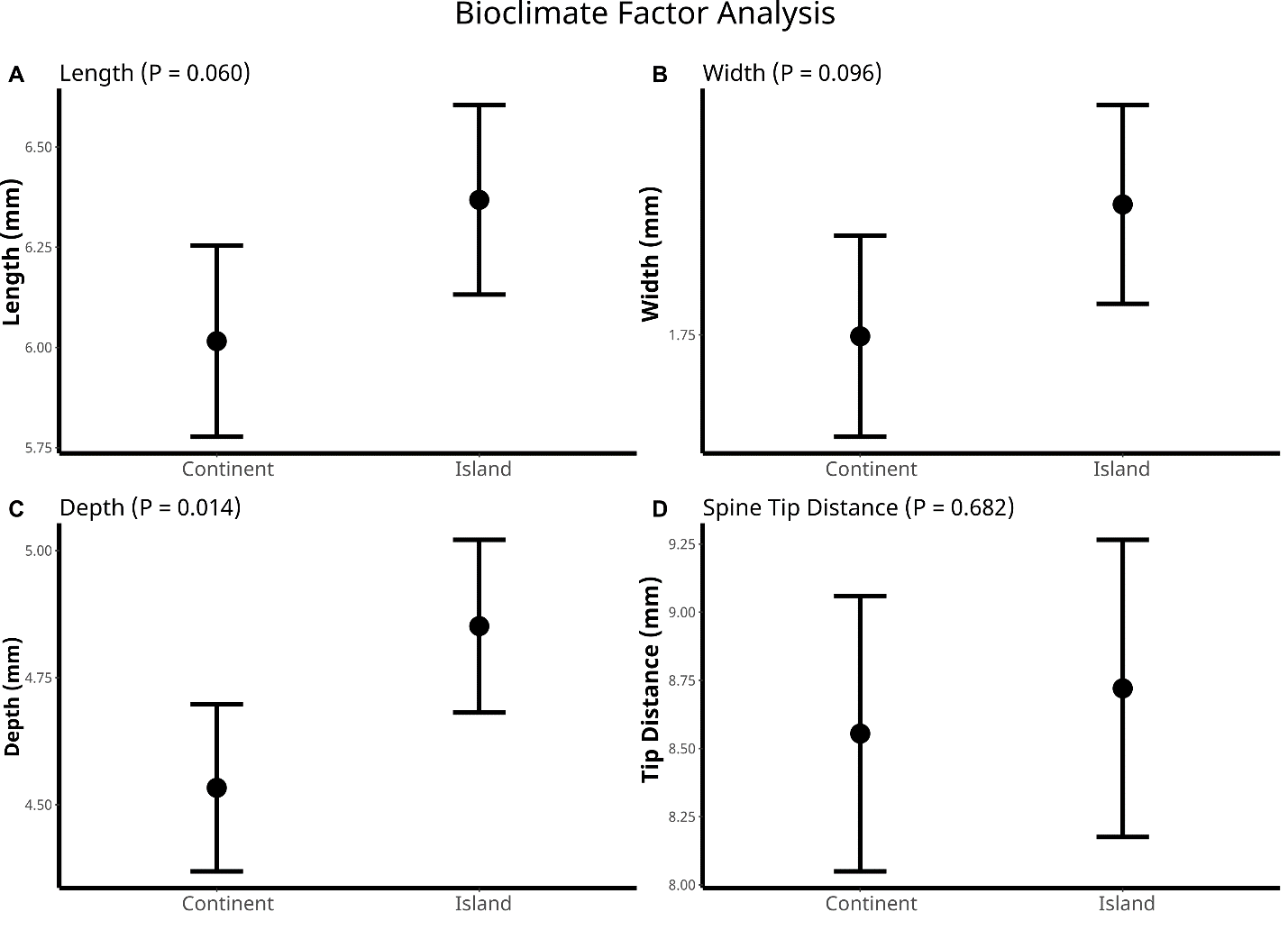


**FIGURE S5**

Mean individual mericarp traits compared between island and continental locations. Means were estimated to account for unbalanced sampling from Galápagos and Florida (n = 3829, n = 1291). Locations were grouped by ID. Plots are the least-squares means ± one standard error. On top of each plot, it shows the p-values from the ANOVA. A-D) Mericarp trait plots include bioclimatic variables.


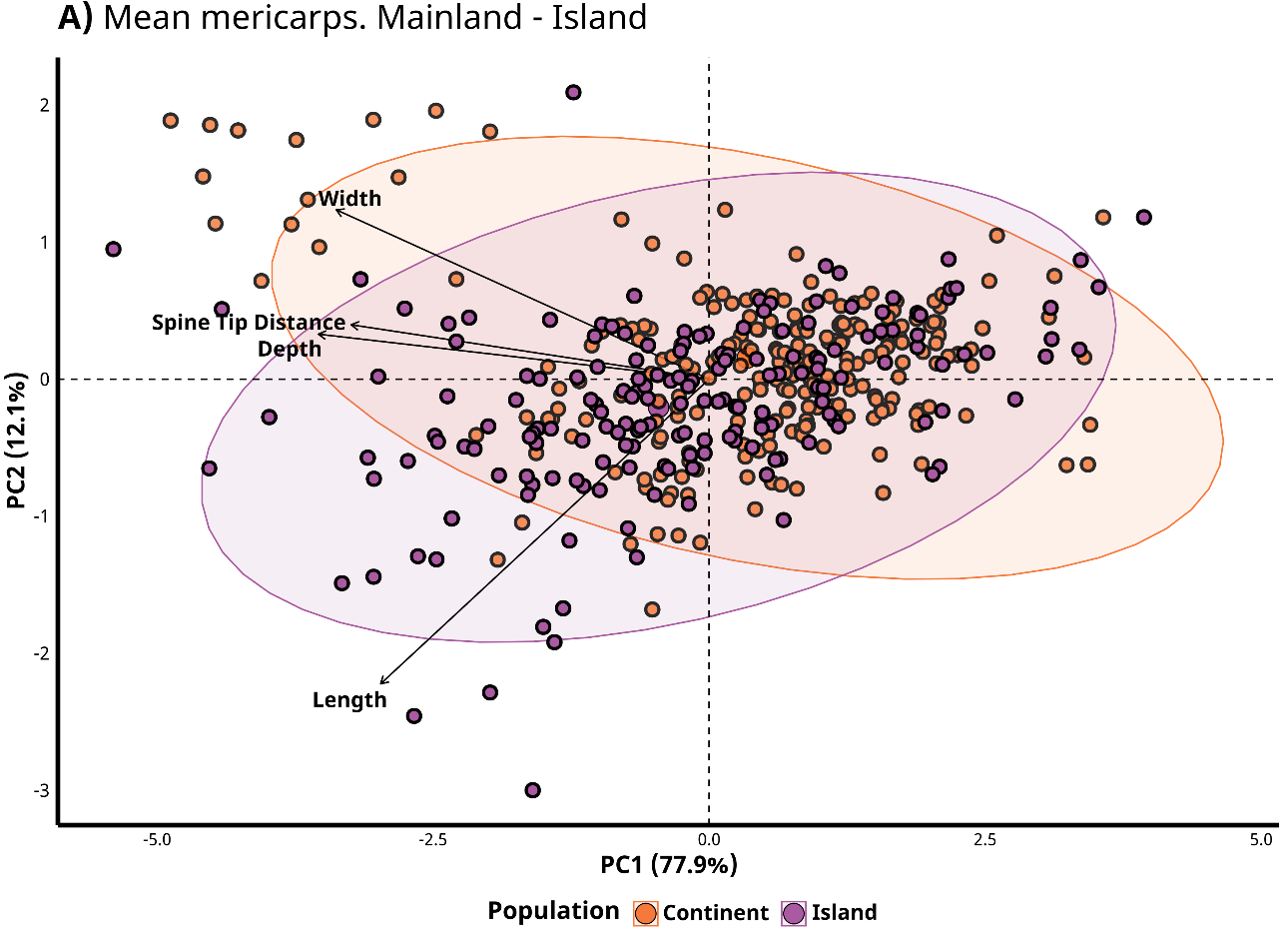


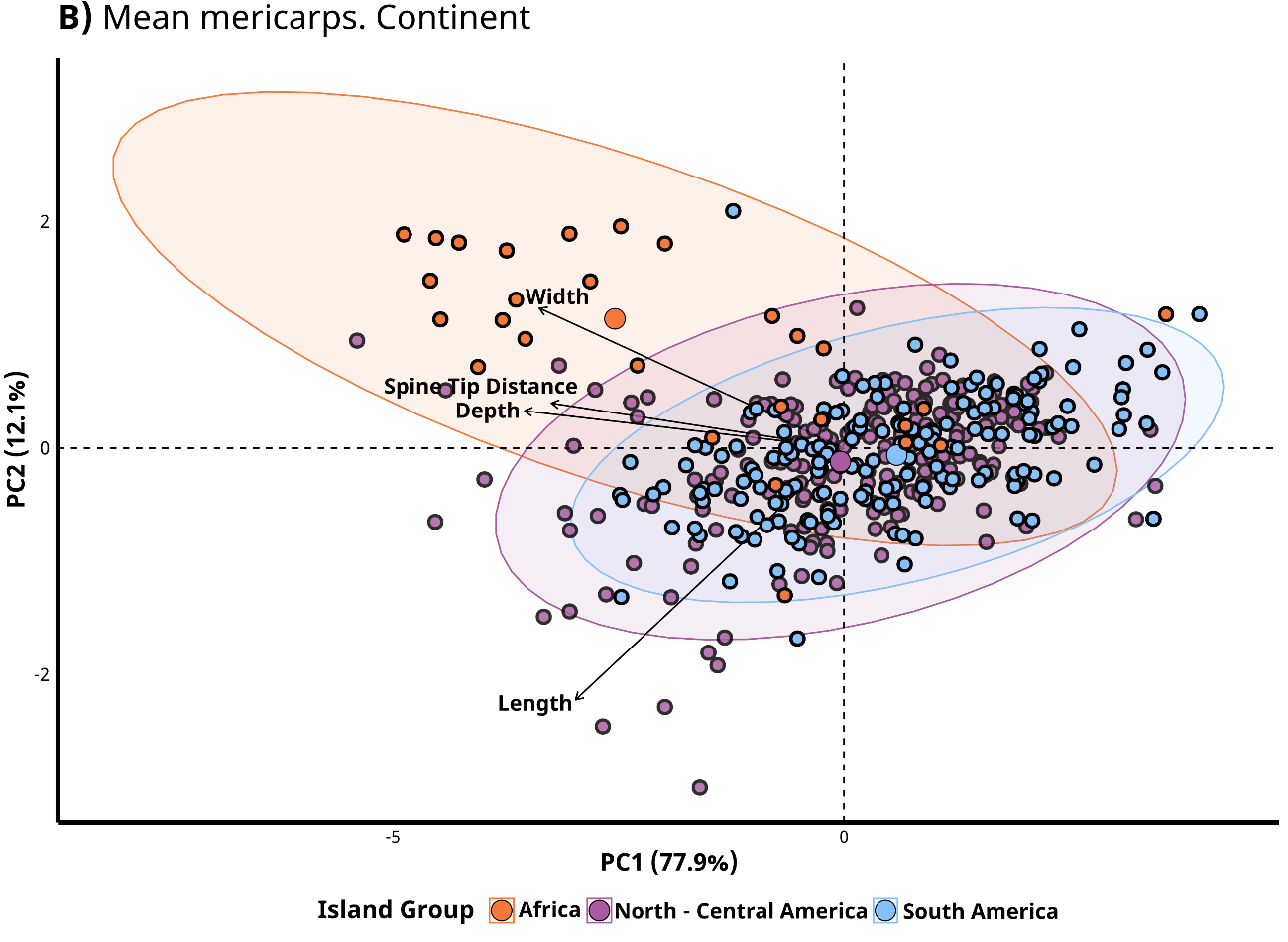


**FIGURE S6**

Principal component analysis of mean mericarp traits, length, depth, width, and spine size. Points represent individual mericarps and estimated means of mericarps for Galápagos and Florida locations. Locations were grouped by ID. Other locations kept their individual values. Means were estimated to account for unbalanced sampling from these locations (n = 3829, n = 1291). Trait vectors are proportional to the contribution and direction associated with each trait. Larger circles represent the centroid of the ellipses with a 95% confidence interval. Top represents PCA with ellipses representing continental and mainland populations. Bottom, PCA representing main continental groups used in the analysis.


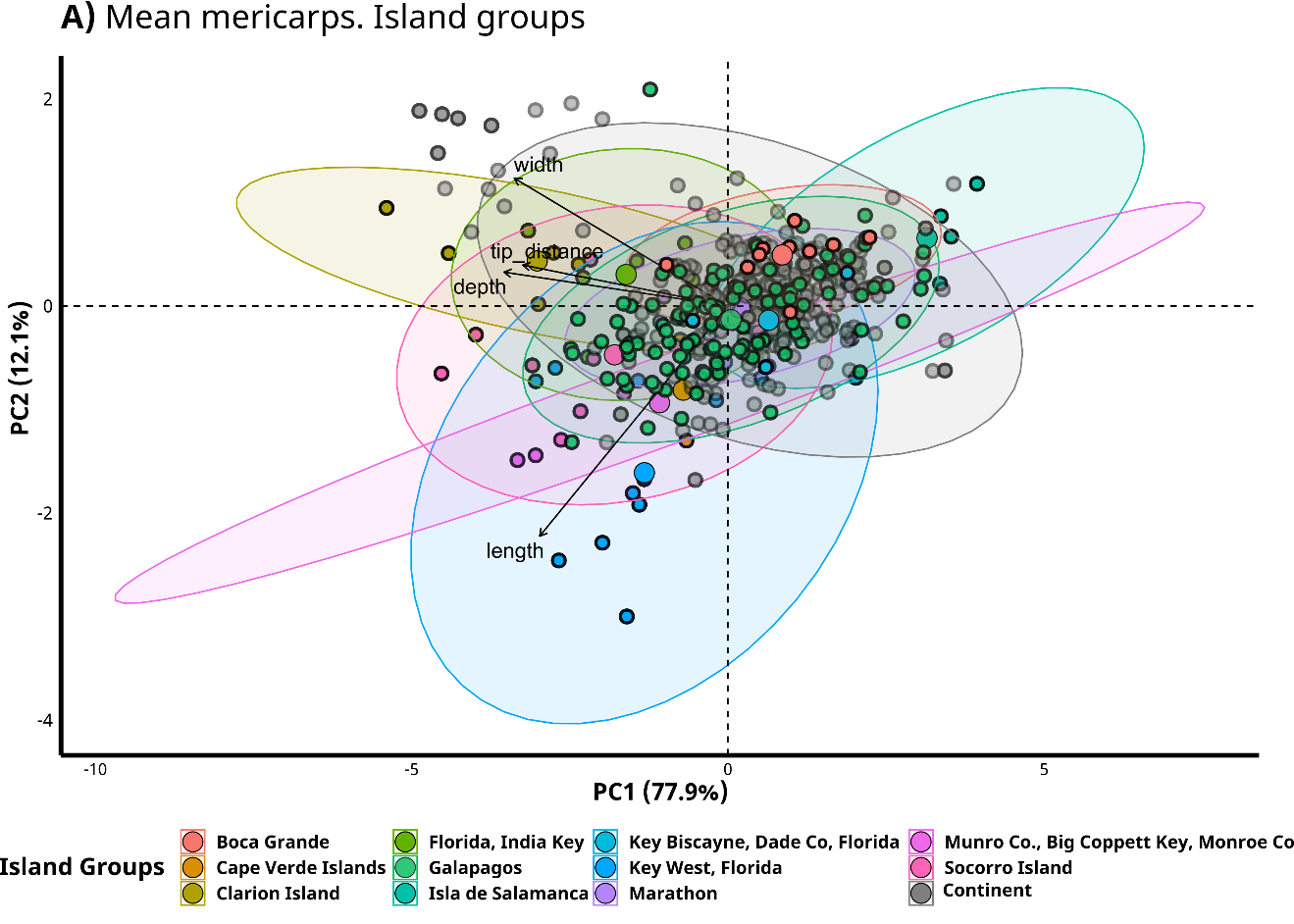


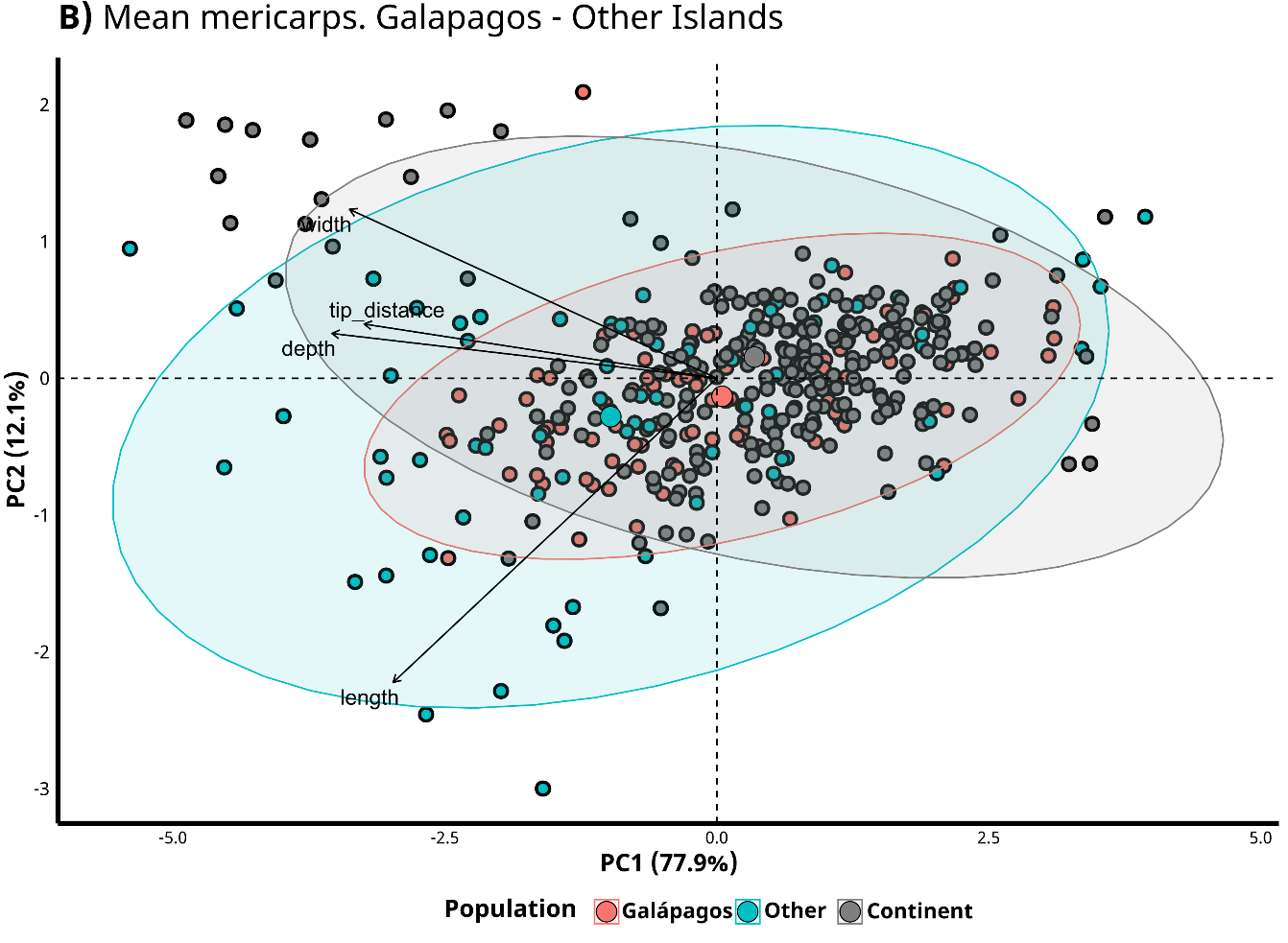


**FIGURE S7**

Principal component analysis of mean mericarp traits, length, depth, width, and spine size. Points represent individual mericarps and estimated means of mericarps for Galápagos and Florida locations. Locations were grouped by ID. Other locations kept their individual values. Means were estimated to account for unbalanced sampling from these locations (n = 3829, n = 1291). Trait vectors are proportional to the contribution and direction associated to each trait. Larger circles represent the centroid of the ellipses with a 95% confidence interval. Top represents PCA with ellipsis representing island groups. Bottom, PCA representing Galápagos and Other Island groups used in the analysis.


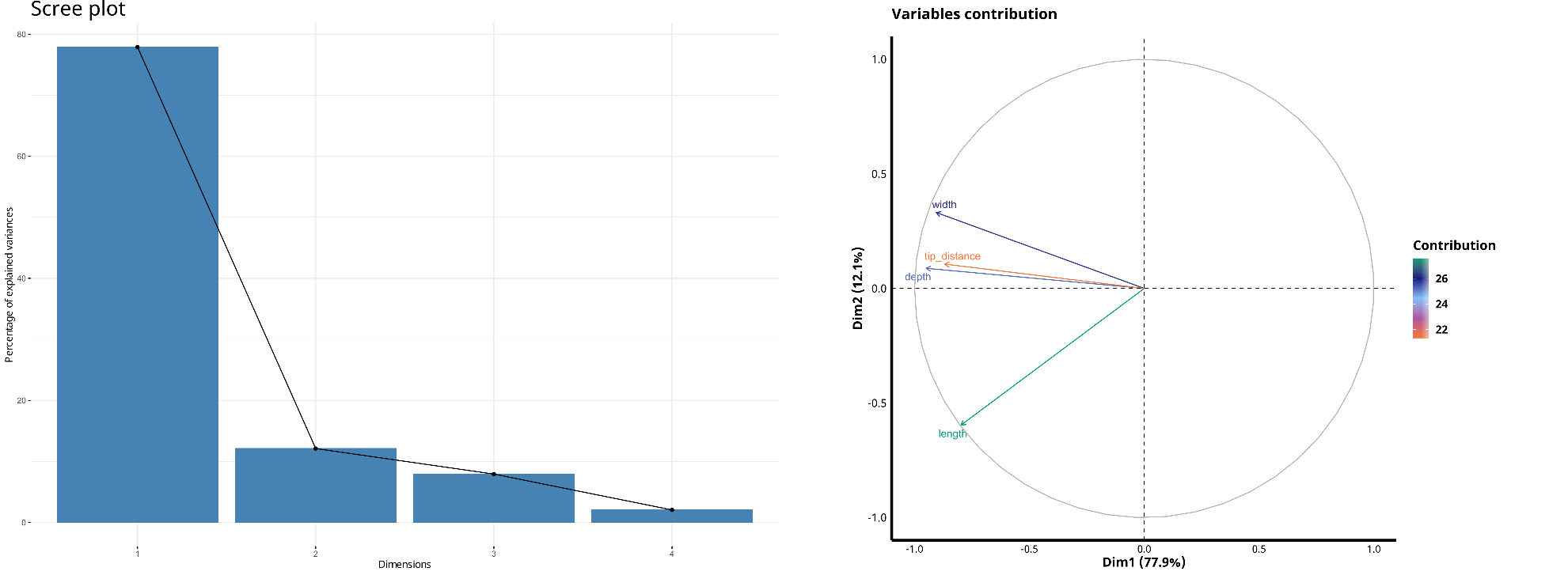


**FIGURE S8**

Eigenvector contribution plot and percentage of explained variation for each PC axis associated with mericarp morphology for mean mericarps of Galápagos and Florida. Locations were grouped by ID. Other locations kept their individual values. Means were estimated to account for unbalanced sampling from these locations (n = 3829, n = 1291)

**APPENDIX S5**

**Table S5**

Model estimates of the effect of population and year of collection on individual mericarp traits including bioclimate variables with African Islands removed. African Islands were samples from a single herbarium voucher and represents only one individual per location. The table shows the model estimates per trait and the PC1 estimates.

| **A) Mericarp – *continental vs island* - African Islands removed** | | | | | | | |
| --- | --- | --- | --- | --- | --- | --- | --- |
|  | **Trait** | **Continental/Island** | | **Year** | | **Herbarium** | |
|  |  | **χ^2^** | **P** | **χ^2^** | **P** | **χ^2^** | **P** |
|  | Length | **18.275** | **<0.001** | **10.179** | **0.001** | 0.7783 | 0.377 |
|  | Width | **12.930** | **<0.001** | 1.019 | 0.312 | 0.164 | 0.684 |
|  | Depth | **55.408** | **<0.001** | **7.989** | **0.004** | 0.203 | 0.651 |
|  | Spine tip distance | **7.086** | **0.007** | 0.163 | 0.686 | 0.519 | 0.470 |
|  | Lower spines | **73.388** | **<0.001** | - | - | 3.077 | 0.0793 |
|  | Mericarp Size (PC1) | **26.374** | **<0.001** | **4.298** | **0.038** | 0.063 | 0.800 |

**TABLE S6**

Model estimates of the effect of population and year of collection on individual mericarp traits including bioclimate variables with African Islands removed. African Islands were samples from a single herbarium voucher and represents only one individual per location. The table shows the model estimates per trait and the PC1 estimates. Nomenclature on bioclimate variables was taken from the WorldClim dataset (<https://worldclim.org/>). We used variables Bio1 (Annual Mean Temperature), Bio4 (Temperature Seasonality), Bio12 (Annual precipitation), Bio15 (Precipitation Seasonality).

| **A) Mericarp – *continental vs island* - African Islands removed** | | | | | | | | | | | | | | | |
| --- | --- | --- | --- | --- | --- | --- | --- | --- | --- | --- | --- | --- | --- | --- | --- |
|  | **Trait** | **Continental/Island** | | **Year** | | **Herbarium** | | **Bio1** | | **Bio4** | | **Bio12** | | **Bio15** | |
|  |  | **χ^2^** | **P** | **χ^2^** | **P** | **χ^2^** | **P** | **χ^2^** | **P** | **χ^2^** | **P** | **χ^2^** | **P** | **χ^2^** | **P** |
|  | Length | 0.463 | 0.495 | **9.824** | **0.001** | 0.014 | 0.905 | 0.068 | 0.068 | **5.699** | **0.016** | **4.096** | **0.042** | 2.150 | 0.142 |
|  | Width | 0.647 | 0.420 | 0.755 | 0.384 | 0.652 | 0.419 | 0.028 | 0.866 | 3.726 | 0.053 | 2.903 | 0.088 | 0.459 | 0.498 |
|  | Depth | 2.645 | 0.103 | **8.212** | **0.004** | 0.068 | 0.794 | 0.031 | 0.860 | 2.494 | 0.114 | **4.445** | **0.034** | **4.276** | **0.038** |
|  | Spine tip distance | 0.010 | 0.918 | 0.272 | 0.601 | 0.012 | 0.910 | 0.456 | 0.499 | **4.089** | **0.043** | **6.971** | **0.008** | 0.039 | 0.843 |
|  | Lower spines | 1.147 | 0.284 | 1.477 | 0.224 | 0.384 | 0.535 | **5.835** | **0.015** | **7.912** | **0.004** | **20.423** | **<0.001** | **15.961** | **<0.001** |
|  | Mericarp Size (PC1) | 0.173 | 0.676 | **4.242** | **0.039** | 0.184 | 0.667 | 0.0004 | 0.984 | **7.119** | **0.007** | **8.852** | **0.002** | 1.345 | 0.246 |


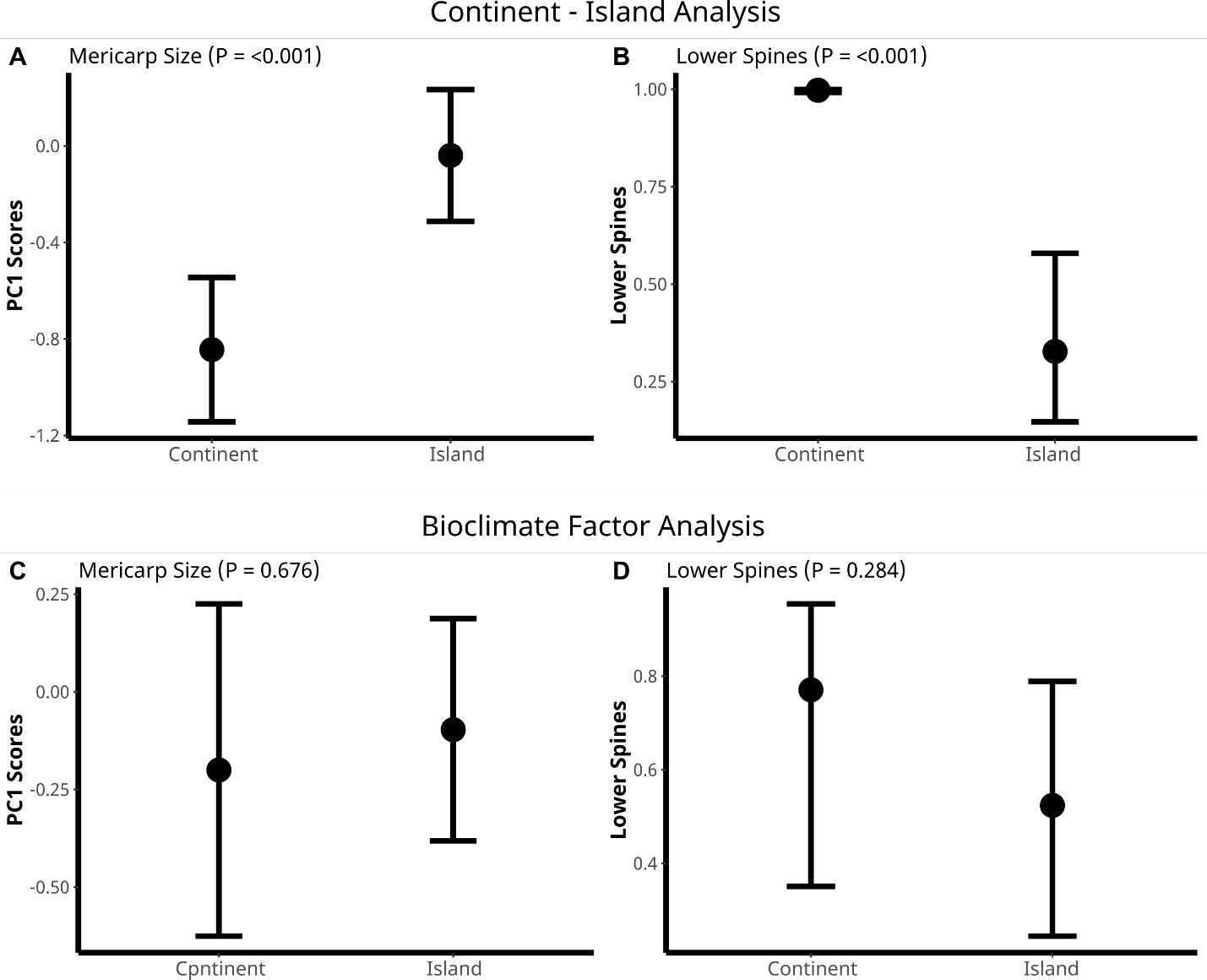


**FIGURE S6**

Mericarp traits compared between island and continental locations with African Island (n=8) samples removed. Plots show the least-squares mean estimates (± 1 SE) using PC1 as a summary of mericarp size (length, width, depth, and spine size) and the presence or absence of lower spines. P-values correspond to the difference between island and continental plants. (A-B) Estimates of continental and island populations only. (C-D) Estimates of the island effect from the model after accounting for bioclimatic variation.


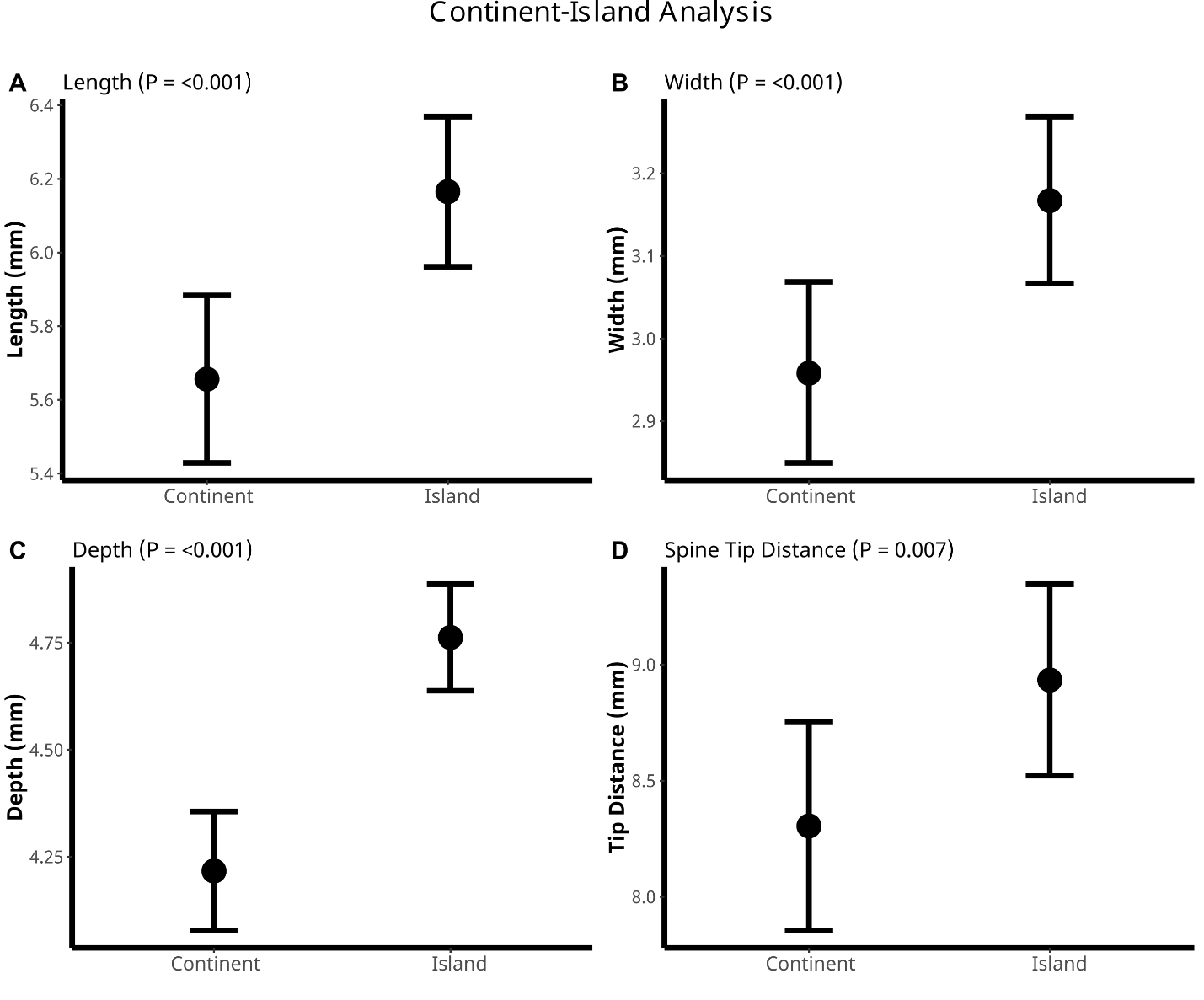


**FIGURE S7**

Individual mericarp traits compared between island and continental locations with African Islands (n =8) samples removed. Plots are the least-squares means ± one standard error. On top of each plot, it shows the p-values from the ANOVA. A-D) Mericarp trait plots without bioclimatic variables included.


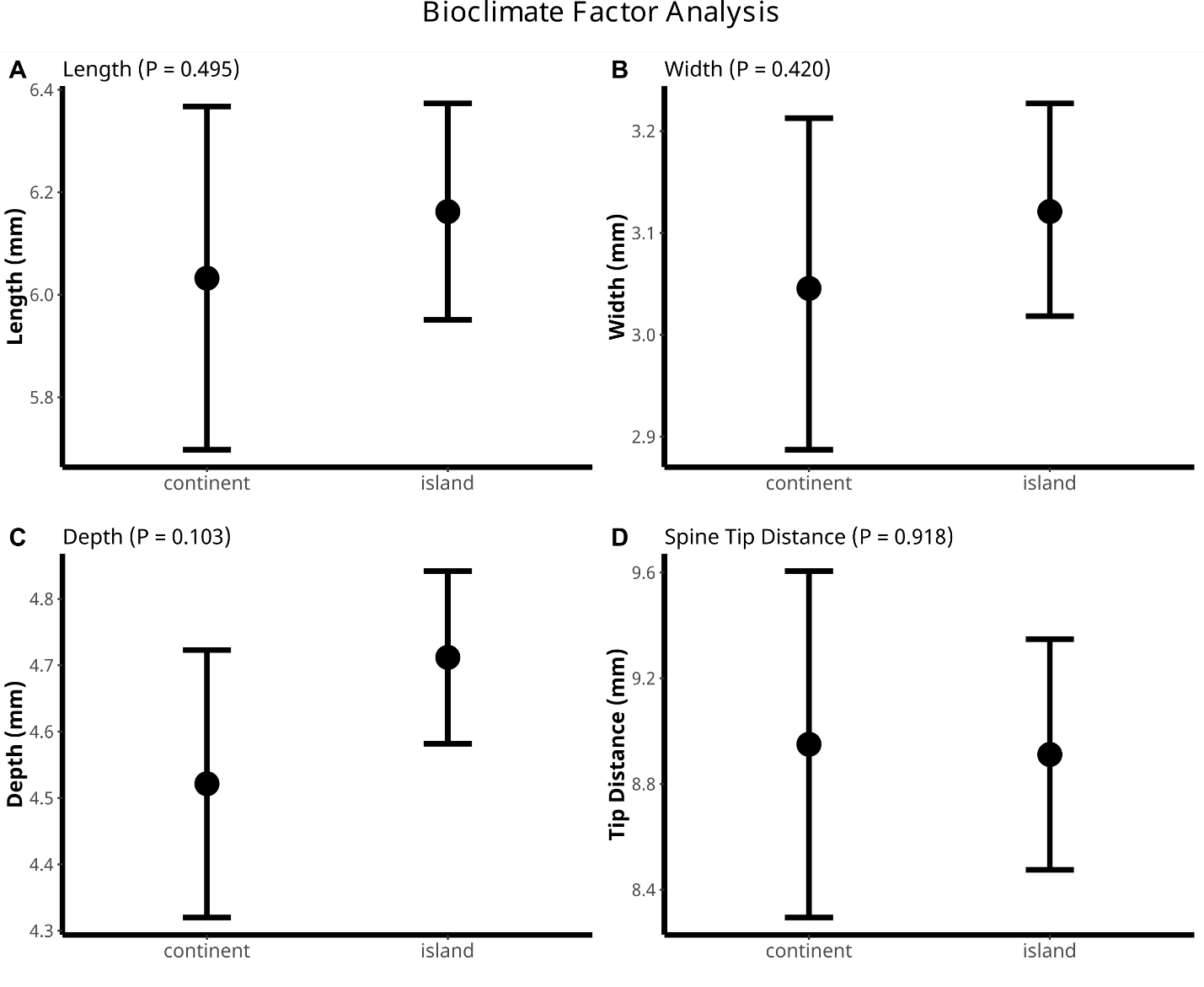


**FIGURE S8**

Individual mericarp traits compared between island and continental locations with African Islands (n =8) samples removed. Plots are the least-squares means ± one standard error. On top of each plot, it shows the p-values from the ANOVA. A-D) Mericarp trait plots include bioclimate variables.

**APPENDIX S6**

**Table S9**

Model estimates of the effect of population and year of collection on individual mericarp and flower traits from Other Islands (removing Galápagos samples) and Continental populations. A) Model estimates per mericarp traits and the PC1 estimates. B) Model estimates for petal length.

| **A) Mericarp – *continental vs island* - *Galápagos removed*** | | | | | | | |
| --- | --- | --- | --- | --- | --- | --- | --- |
|  | **Trait** | **Continental/Island** | | **Year** | | **Herbarium** | |
|  |  | **χ^2^** | **P** | **χ^2^** | **P** | **χ^2^** | **P** |
|  | Length | 0.072 | 0.787 | **31.511** | **<0.001** | **10.341** | **0.001** |
|  | Width | 2.795 | 0.094 | 0.991 | 0.319 | 0.018 | 0.893 |
|  | Depth | **5.843** | **0.015** | **5.662** | **0.017** | 0.033 | 0.855 |
|  | Spine tip distance | **4.591** | **0.032** | 0.039 | 0.842 | 0.002 | 0.957 |
|  | Lower spines | 1.342 | 0.246 | - | - | **5.364** | **0.020** |
|  | Mericarp Size (PC1) | 3.252 | 0.071 | **6.054** | **0.013** | 0.526 | 0.467 |
| **B) Flowers – *continental vs island* - *Galápagos removed*** | | | | | | | |
|  | Petal length | 2.440 | 0.118 | **9.040** | **0.002** | - | - |

**TABLE S10**

Model estimates of the effect of population and year of collection on individual mericarp and flower traits including bioclimate variables from Other Islands (removing Galápagos samples) and Continental populations. A) Model estimates per mericarp traits and the PC1 estimates. B) Model estimates for petal length. Nomenclature on bioclimate variables was taken from the WorldClim dataset (<https://worldclim.org/>). We used variables Bio1 (Annual Mean Temperature), Bio4 (Temperature Seasonality), Bio12 (Annual precipitation), Bio15 (Precipitation Seasonality).

| **A) Mericarp – *continental vs island - Galápagos removed*** | | | | | | | | | | | | | | | |
| --- | --- | --- | --- | --- | --- | --- | --- | --- | --- | --- | --- | --- | --- | --- | --- |
|  | **Trait** | **Continental/Island** | | **Year** | | **Herbarium** | | **Bio1** | | **Bio4** | | **Bio12** | | **Bio15** | |
|  |  | **χ^2^** | **P** | **χ^2^** | **P** | **χ^2^** | **P** | **χ^2^** | **P** | **χ^2^** | **P** | **χ^2^** | **P** | **χ^2^** | **P** |
|  | Length | 0.016 | 0.898 | **17.491** | **<0.001** | 0.871 | 0.350 | 0.059 | 0.807 | 3.453 | 0.063 | 0.337 | 0.561 | 0.100 | 0.751 |
|  | Width | 1.063 | 0.302 | 0.010 | 0.918 | 1.462 | 1.462 | 0.046 | 0.829 | **4.199** | **0.040** | **4.316** | **0.037** | 0.072 | 0.787 |
|  | Depth | 1.713 | 0.190 | 1.446 | 0.229 | 0.313 | 0.575 | 0.083 | 0.772 | **3.891** | **0.048** | 3.027 | 0.081 | **6.717** | **0.009** |
|  | Spine tip distance | 2.993 | 0.083 | 1.429 | 0.231 | 2.501 | 0.113 | 0.008 | 0.925 | **4.767** | **0.029** | **18.585** | **<0.001** | **8.598** | **0.003** |
|  | Lower spines | 0.608 | 0.435 | 0 | 0.999 | 1.033 | 0.309 | 1.115 | 0.290 | 0.027 | 0.868 | 1.991 | 0.158 | **6.906** | **0.008** |
|  | Mericarp Size (PC1) | 1.022 | 0.311 | 0.932 | 0.334 | 0.758 | 0.383 | 0.232 | 0.629 | **6.009** | **0.014** | **9.032** | **0.002** | 0.128 | 0.72 |
| **B) Flowers – *continental vs island - Galápagos removed*** | | | | | | | | | | | | | | | |
|  | **Trait** | **Continental/Island** | | **Year** | | **Herbarium** | | **Bio1** | | **Bio4** | | **Bio12** | | **Bio15** | |
|  |  | **χ^2^** | **P** | **χ^2^** | **P** | **χ^2^** | **P** | **χ^2^** | **P** | **χ^2^** | **P** | **χ^2^** | **P** | **χ^2^** | **P** |
|  | Petal length | 0.004 | 0.947 | **7.880** | **0.004** | - | - | **4.781** | **0.028** | **8.149** | **0.004** | 1.353 | 0.244 | **7.630** | **0.005** |


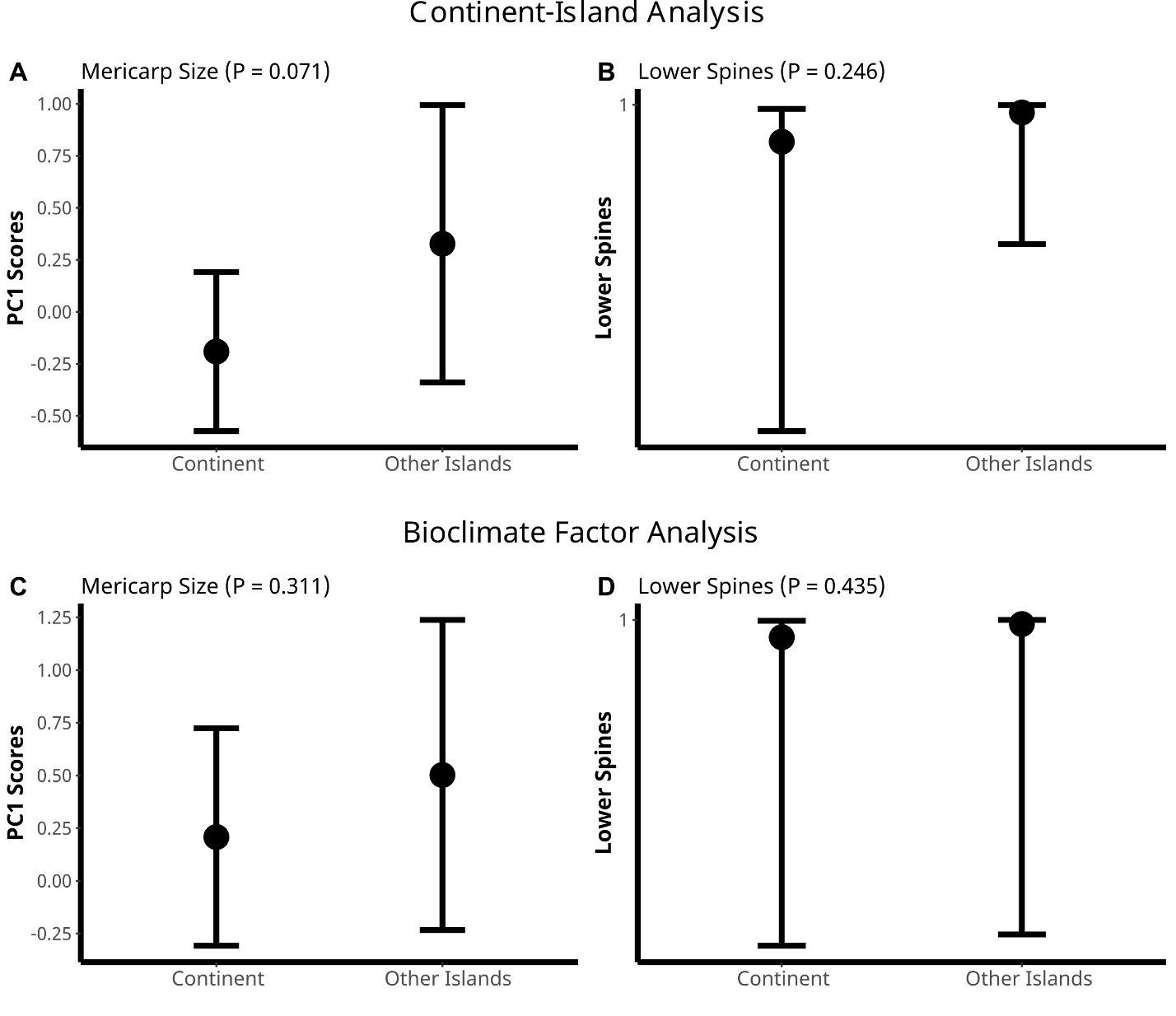


**FIGURE S9**

Mericarp traits compared between the Other Islands and continental populations. Plots show the least-squares mean estimates (± 1 SE) using PC1 as a summary of mericarp size (length, width, depth, and spine size) and the presence or absence of lower spines. P-values correspond to the difference between island and continental plants. (A-B) Estimates of continental and island populations only. (C-D) Estimates of the island effect from the model after accounting for bioclimatic variation.


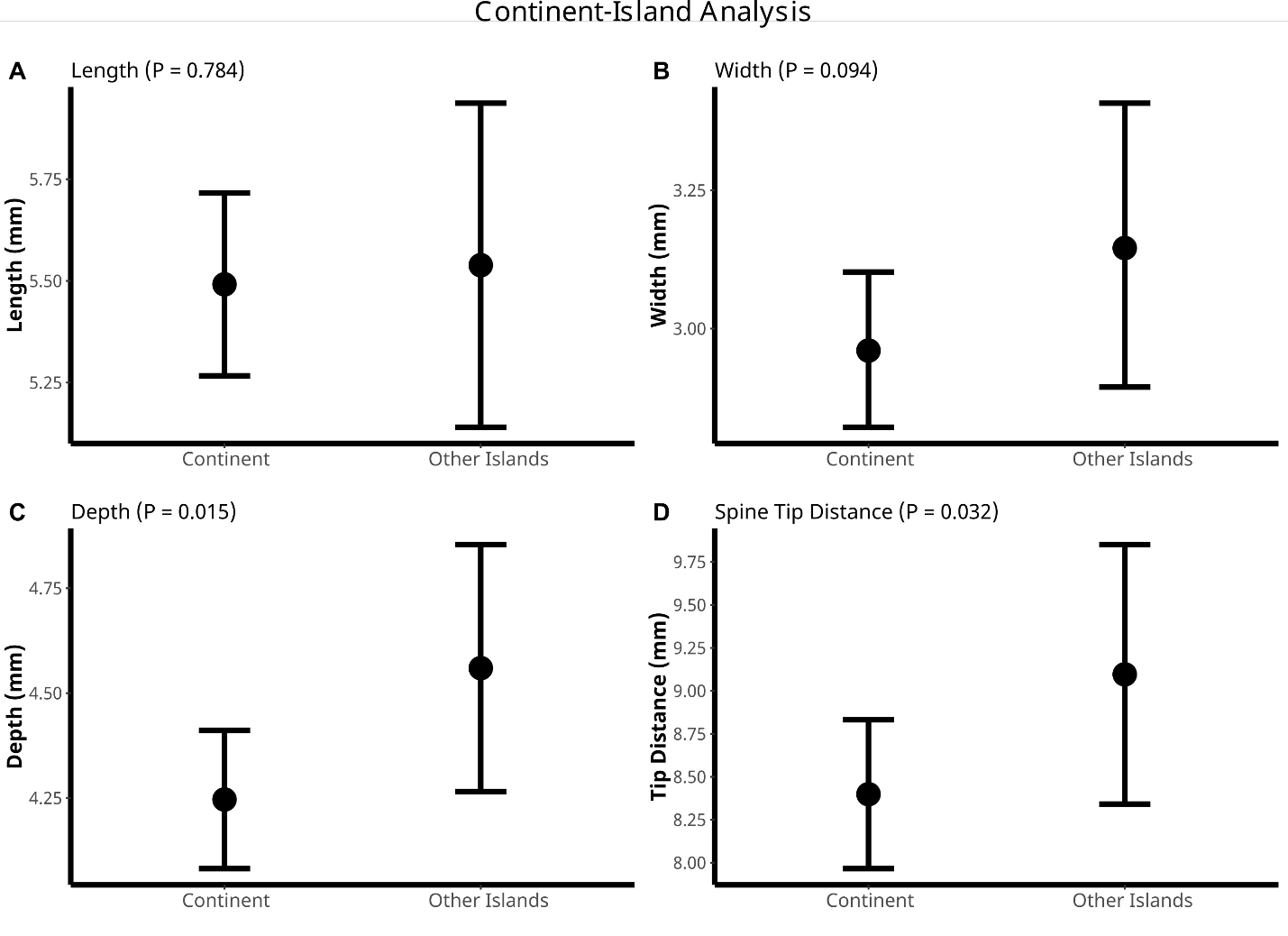


**FIGURE S10**

Individual mericarp traits compared between the Other Islands and continental populations. Plots are the least-squares means ± one standard error. On top of each plot, it shows the p-values from the ANOVA. A-D) Mericarp trait plots without bioclimate variables.


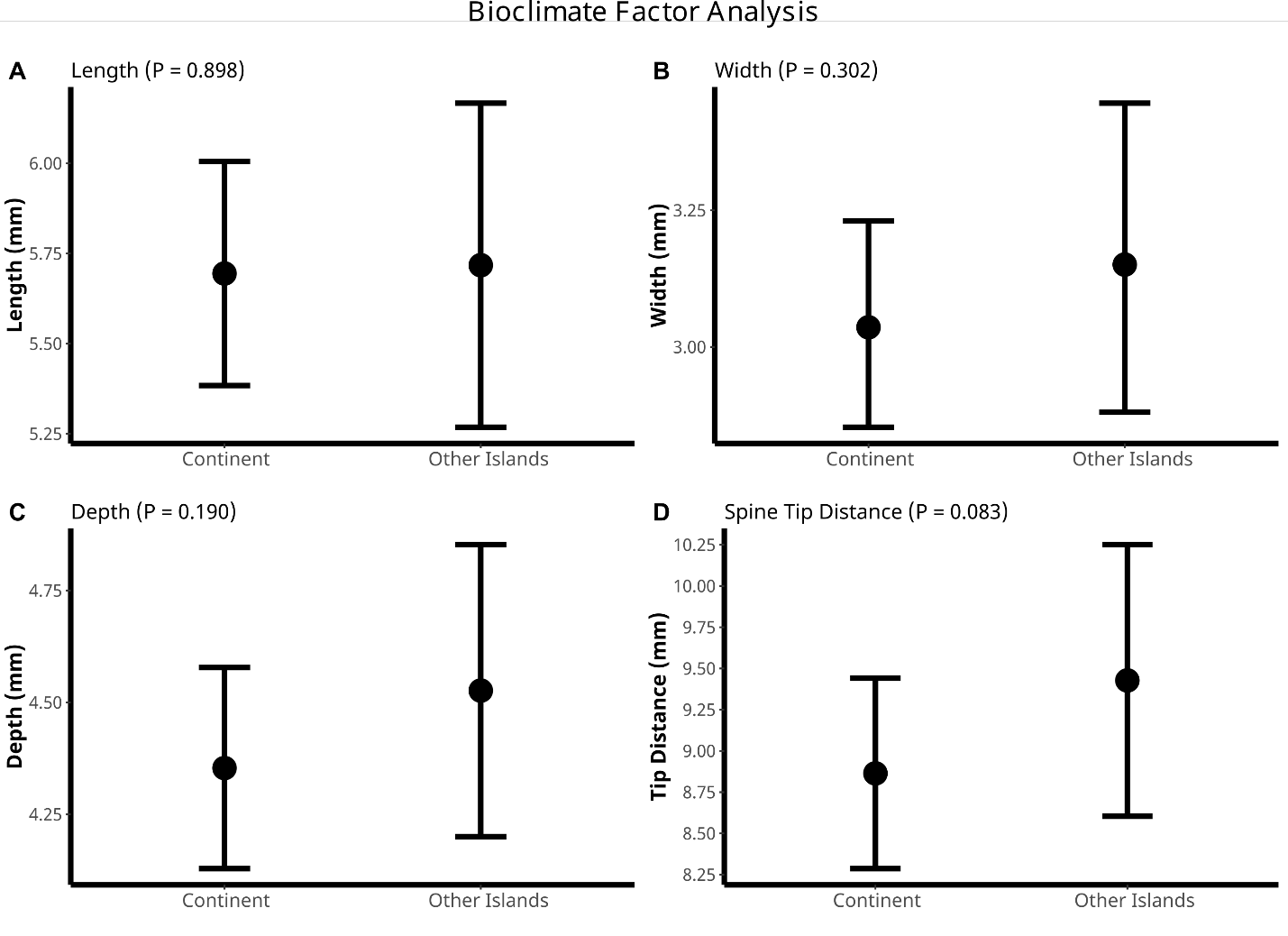


**FIGURE S11**

Individual mericarp traits compared between the Other Islands and continental populations. Plots are the least-squares means ± one standard error. On top of each plot, it shows the p-values from the ANOVA. A-D) Mericarp trait plots include bioclimate variables.


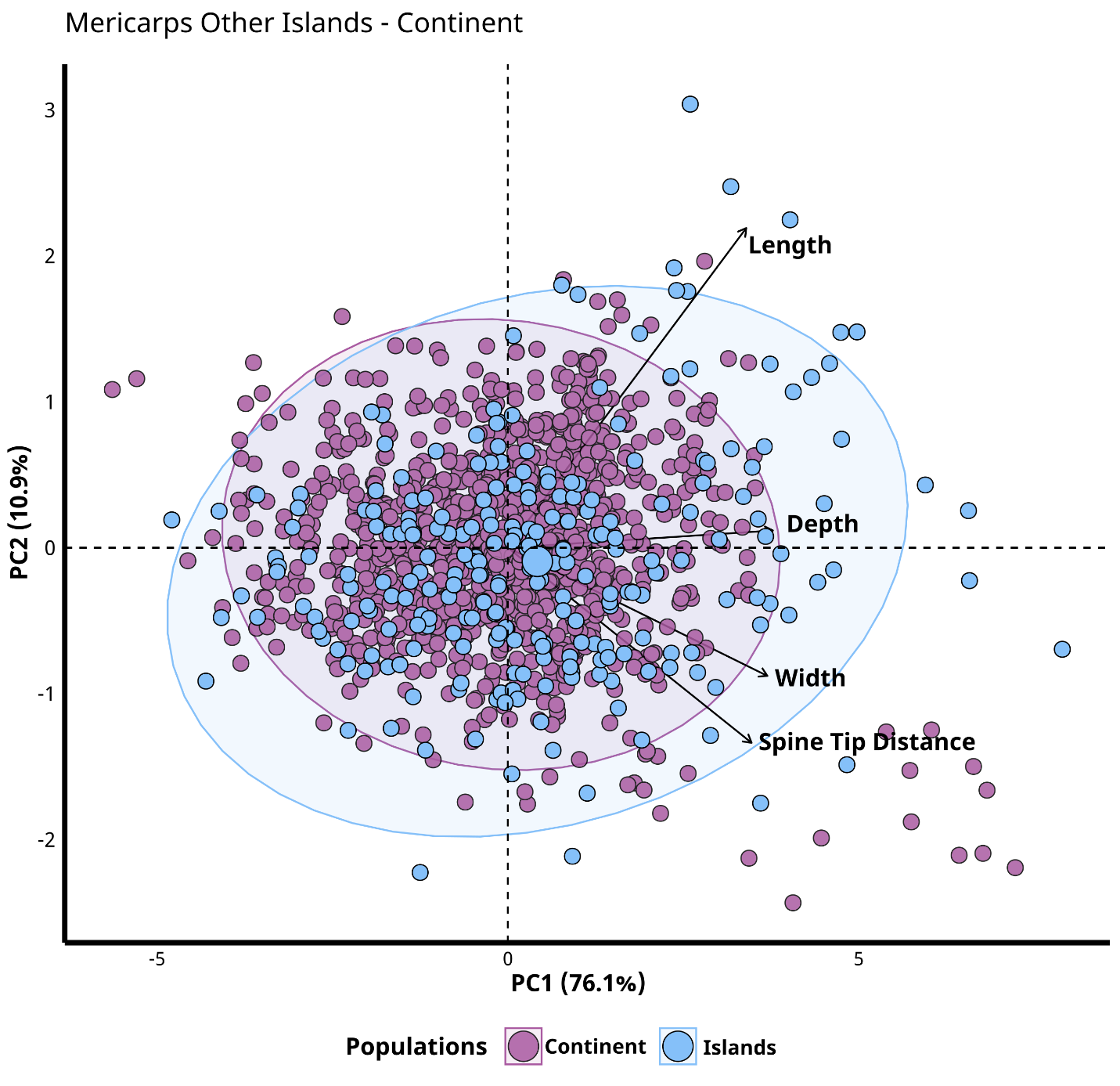


**FIGURE S12**

Principal component analysis of mean mericarp traits, length, depth, width, and spine size. Points represent individual mericarps from other islands and continental populations. Trait vectors are proportional to the contribution and direction associated to each trait. Larger circles represent the centroid of the ellipses with a 95% confidence interval.


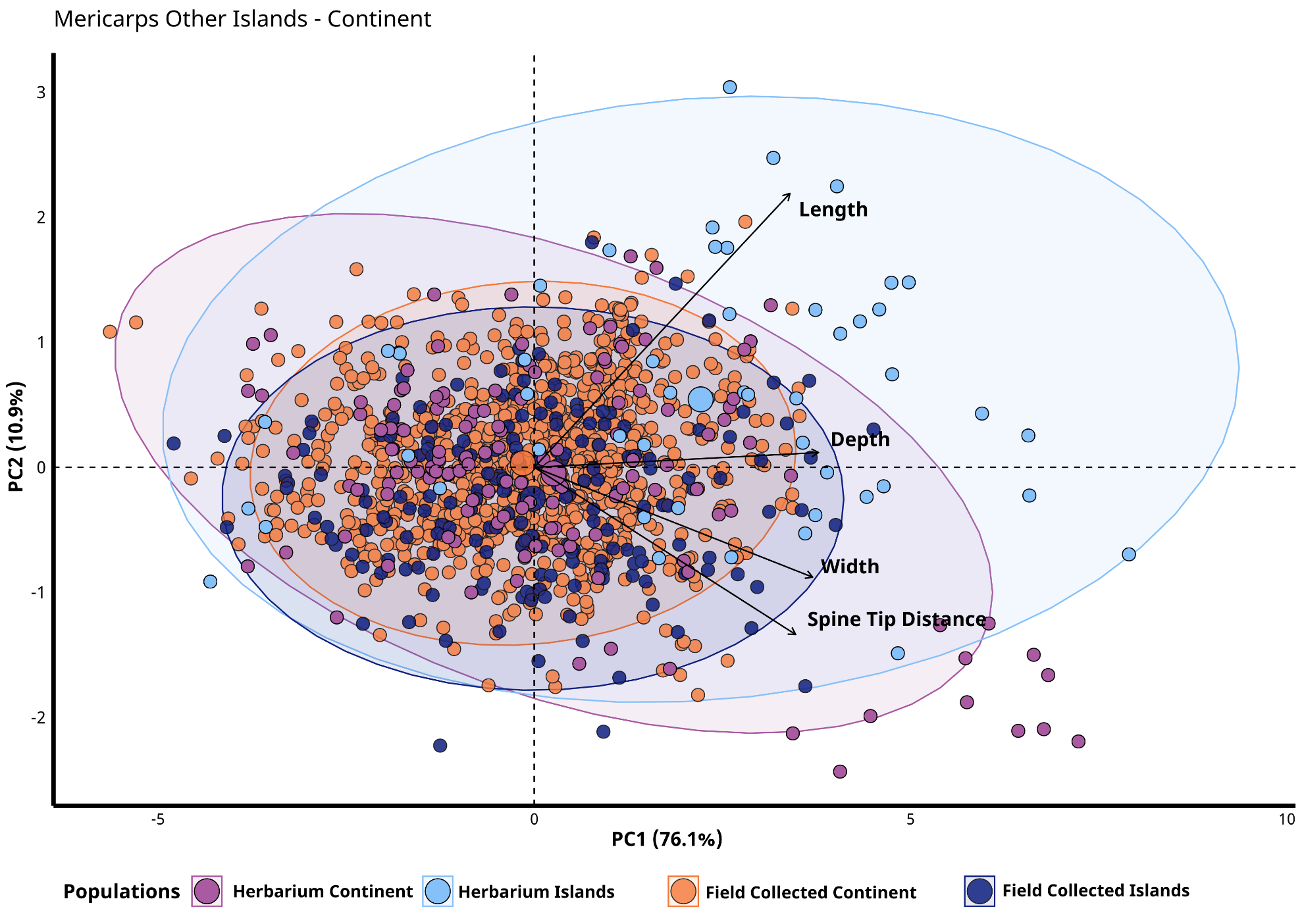


**FIGURE S13**

Principal component analysis of mean mericarp traits, length, depth, width, and spine size. Points represent individual mericarps divided into groups from herbarium and field collected samples from other islands and continent populations. Trait vectors are proportional to the contribution and direction associated to each trait. Larger circles represent the centroid of the ellipses with a 95% confidence interval.


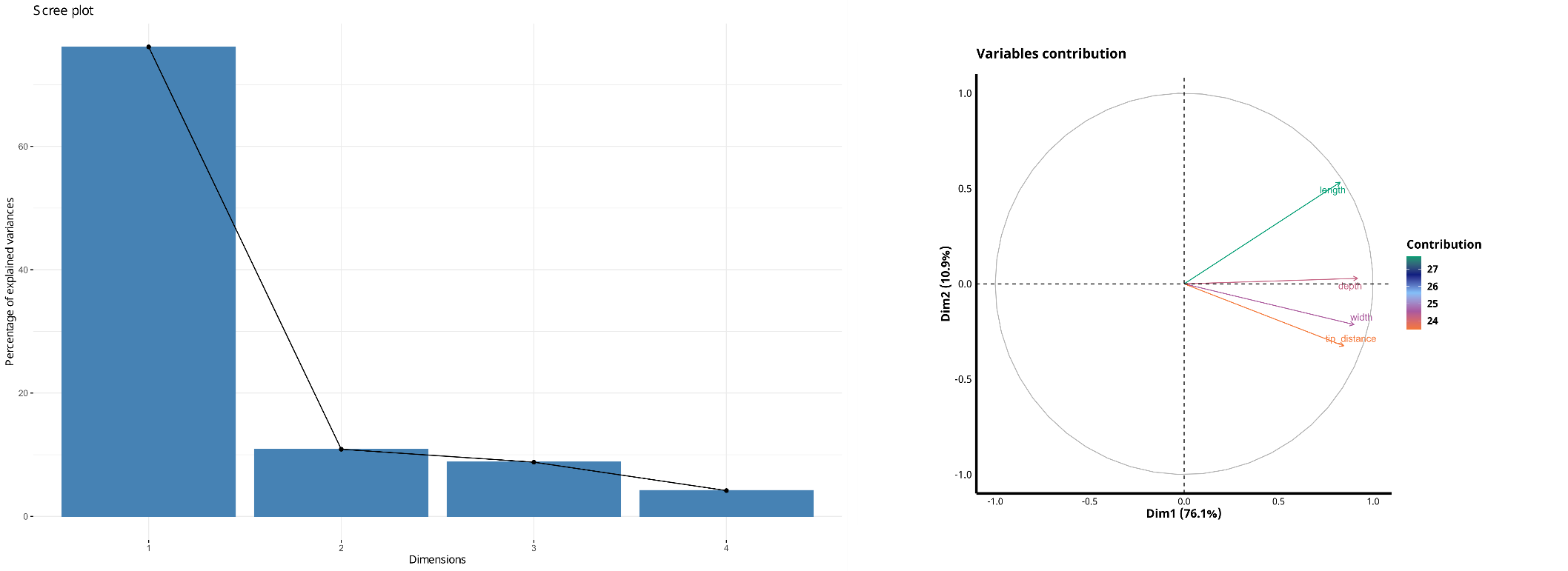


**FIGURE S14**

Eigenvector contribution plot and percentage of explained variation for each PC axis associated with mericarp morphology for other islands and continental populations.


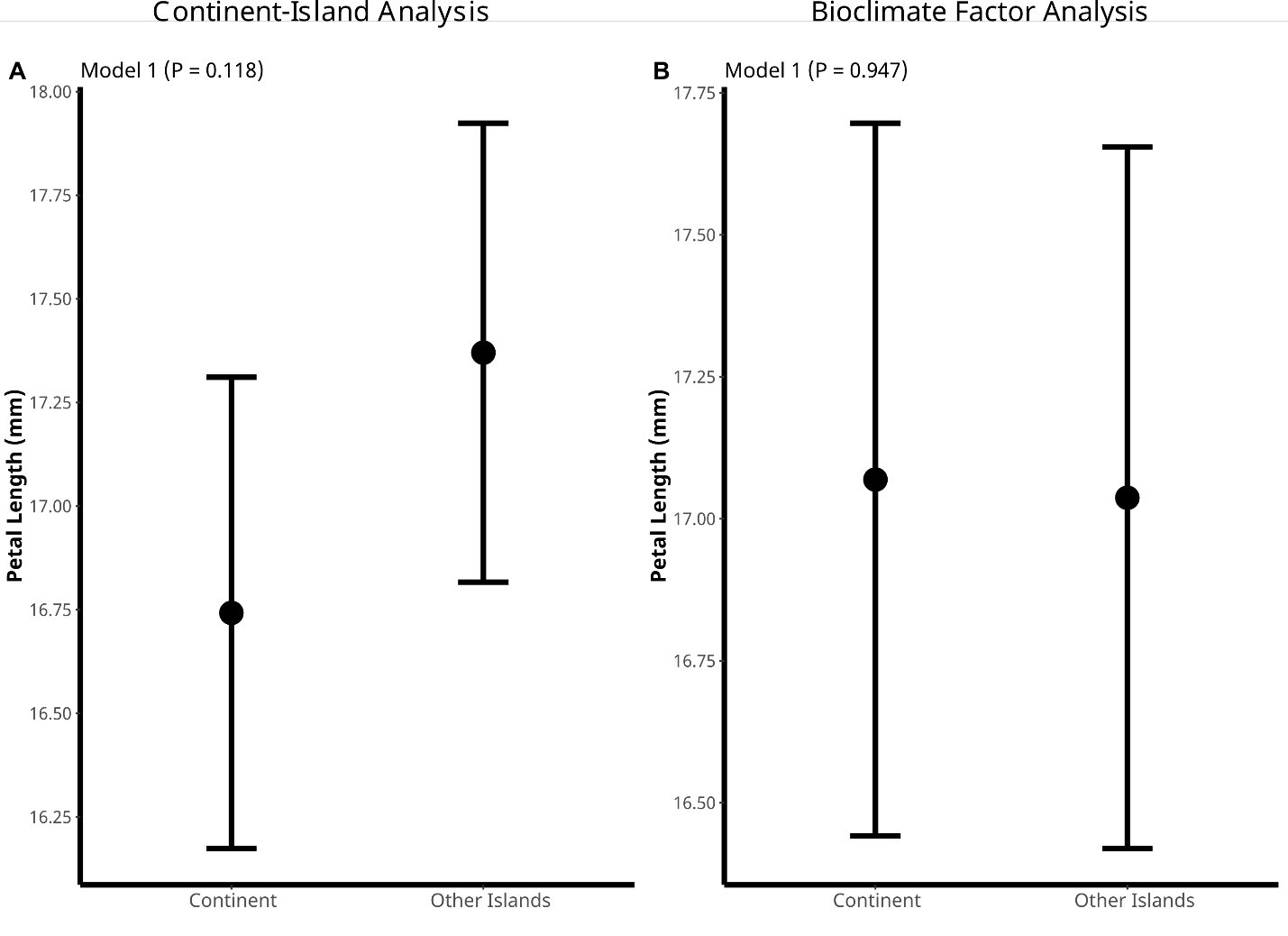


**FIGURE S15**

Petal length estimates from other islands and continental plants. The plots show the least-squares mean estimates (± 1 SE) using petal length. P-values are shown on top of each plot. (A) Estimates of continental and island populations only. (B) Estimates including bioclimate variables.

**APPENDIX S7**

**Table S11**

Model estimates of the effect of population and year of collection on individual mericarp and flower traits from Galápagos and Continental populations only. A) Model estimates per mericarp traits and the PC1 estimates. B) Model estimates for petal length.

| **A) Mericarp – *Galápagos - Continent*** | | | | | | | |
| --- | --- | --- | --- | --- | --- | --- | --- |
|  | **Trait** | **Continental/Island** | | **Year** | | **Herbarium** | |
|  |  | **χ^2^** | **P** | **χ^2^** | **P** | **χ^2^** | **P** |
|  | Length | **15.495** | **<0.001** | 0.021 | 0.883 | 0.002 | 0.957 |
|  | Width | **12.327** | **<0.001** | 0.463 | 0.496 | 0.039 | 0.843 |
|  | Depth | **59.523** | **<0.001** | 3.361 | 0.066 | 0.163 | 0.685 |
|  | Spine tip distance | **5.083** | **0.024** | 0.251 | 0.615 | 0.948 | 0.33 |
|  | Lower spines | **78.411** | **<0.001** | - | - | 3.603 | 0.057 |
|  | Mericarp Size (PC1) | **26.227** | **<0.001** | 1.490 | 0.222 | 0.059 | 0.807 |
| **B) Flowers – *Galápagos vs Continent*** | | | | | | | |
|  |  | **Continental/Island** | | **Year** | | **Herbarium** | |
|  |  | **χ^2^** | **P** | **χ^2^** | **P** | **χ^2^** | **P** |
|  | Petal length | **97.100** | **<0.001** | **5.842** | **0.015** | - | - |

**TABLE S12**

Model estimates of the effect of population and year of collection on individual mericarp and flower traits including bioclimate variables from Galápagos and Continental only. A) Model estimates per mericarp traits and the PC1 estimates. B) Model estimates for petal length. Nomenclature on bioclimate variables was taken from the WorldClim dataset (<https://worldclim.org/>). We used variables Bio1 (Annual Mean Temperature), Bio4 (Temperature Seasonality), Bio12 (Annual precipitation), Bio15 (Precipitation Seasonality).

| **A) Mericarp – *Galápagos - Continent*** | | | | | | | | | | | | | | | |
| --- | --- | --- | --- | --- | --- | --- | --- | --- | --- | --- | --- | --- | --- | --- | --- |
|  | **Trait** | **Continental/Island** | | **Year** | | **Herbarium** | | **Bio1** | | **Bio4** | | **Bio12** | | **Bio15** | |
|  |  | **χ^2^** | **P** | **χ^2^** | **P** | **χ^2^** | **P** | **χ^2^** | **P** | **χ^2^** | **P** | **χ^2^** | **P** | **χ^2^** | **P** |
|  | Length | 2.203 | 0.137 | 0.046 | 0.829 | 0.249 | 0.617 | 0 | 0.995 | 2.854 | 0.091 | 0.004 | 0.949 | **5.276** | **0.021** |
|  | Width | 3.090 | 0.078 | 0.467 | 0.494 | 0.004 | 0.945 | 0.001 | 0.967 | 0.07 | 0.790 | 0.640 | 0.423 | 0.423 | 0.504 |
|  | Depth | **10.159** | **0.001** | 3.293 | 0.069 | 0.716 | 0.397 | 0.191 | 0.661 | 0.788 | 0.374 | 2.575 | 0.108 | **5.974** | **0.014** |
|  | Spine tip distance | 0.066 | 0.796 | 0.249 | 0.617 | 1.064 | 0.302 | 0.904 | 0.341 | 0.067 | 0.795 | 1.212 | 0.270 | 0.010 | 0.919 |
|  | Lower spines | **17.470** | **<0.001** | 0.158 | 0.691 | 0.225 | 0.635 | 2.039 | 0.153 | **0.153** | **0.014** | 0.573 | 0.449 | **16.087** | **<0.001** |
|  | Mericarp Size (PC1) | 1.807 | 0.178 | 1.507 | 0.219 | 0.091 | 0.762 | 0.199 | 0.655 | 0.025 | 0.872 | 0.005 | 0.941 | 2.775 | 0.095 |
| **B) Flowers – *Galápagos vs Continent*** | | | | | | | | | | | | | | | |
|  | **Trait** | **Continental/Island** | | **Year** | | **Herbarium** | | **Bio1** | | **Bio4** | | **Bio12** | | **Bio15** | |
|  |  | **χ^2^** | **P** | **χ^2^** | **P** | **χ^2^** | **P** | **χ^2^** | **P** | **χ^2^** | **P** | **χ^2^** | **P** | **χ^2^** | **P** |
|  | Petal length | **64.747** | **<0.001** | **5.489** | **0.019** | - | - | **11.319** | **<0.001** | **6.614** | **0.010** | 0.434 | 0.509 | **11.044** | **<0.001** |


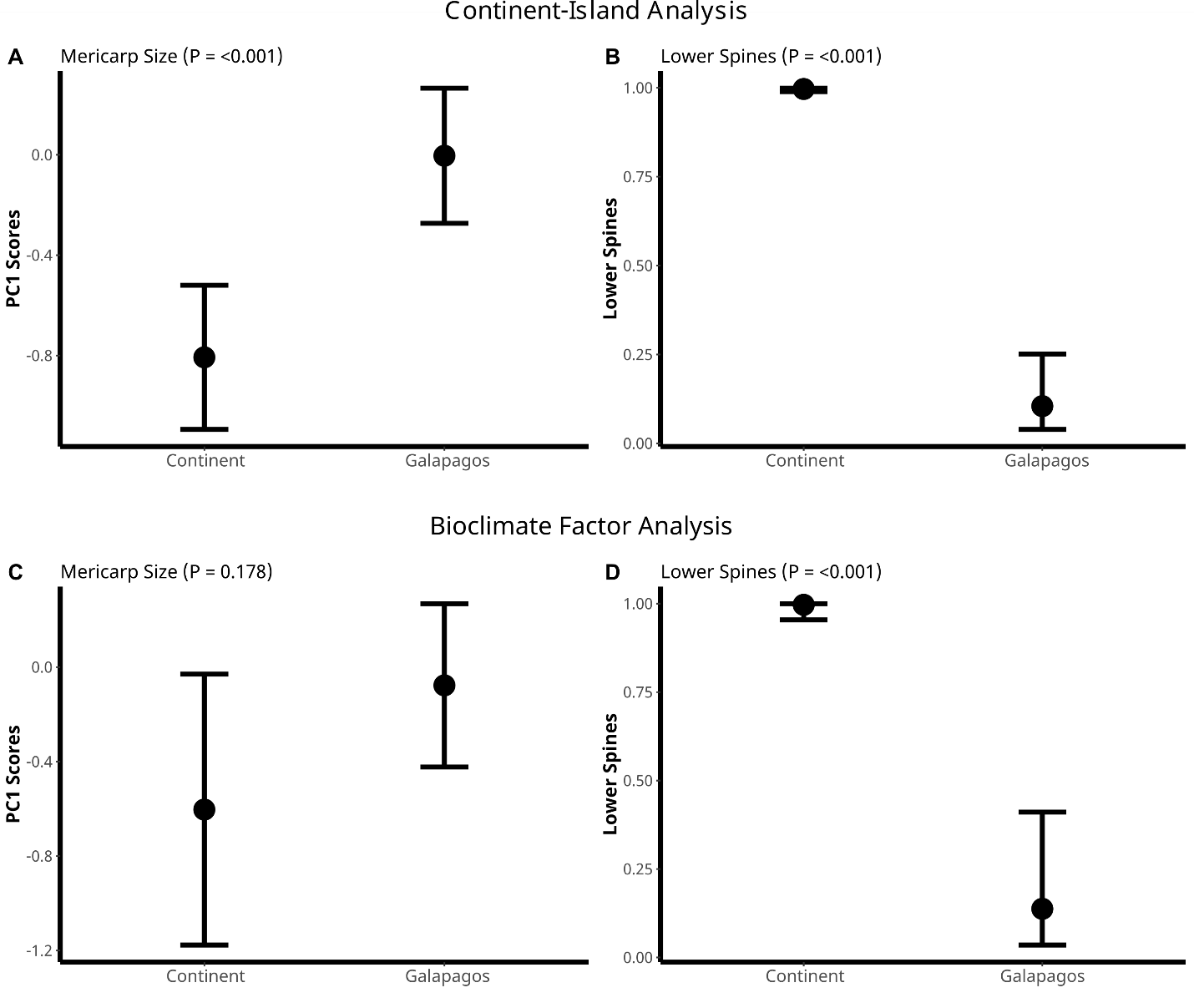


**FIGURE S16**

Mericarp traits compared between the Galápagos Islands and continental populations. Plots show the least-squares mean estimates (± 1 SE) using PC1 as a summary of mericarp size (length, width, depth, and spine size) and the presence or absence of lower spines. P-values correspond to the difference between island and continental plants. (A-B) Estimates of continental and island populations only. (C-D) Estimates of the island effect from the model after accounting for bioclimatic variation.


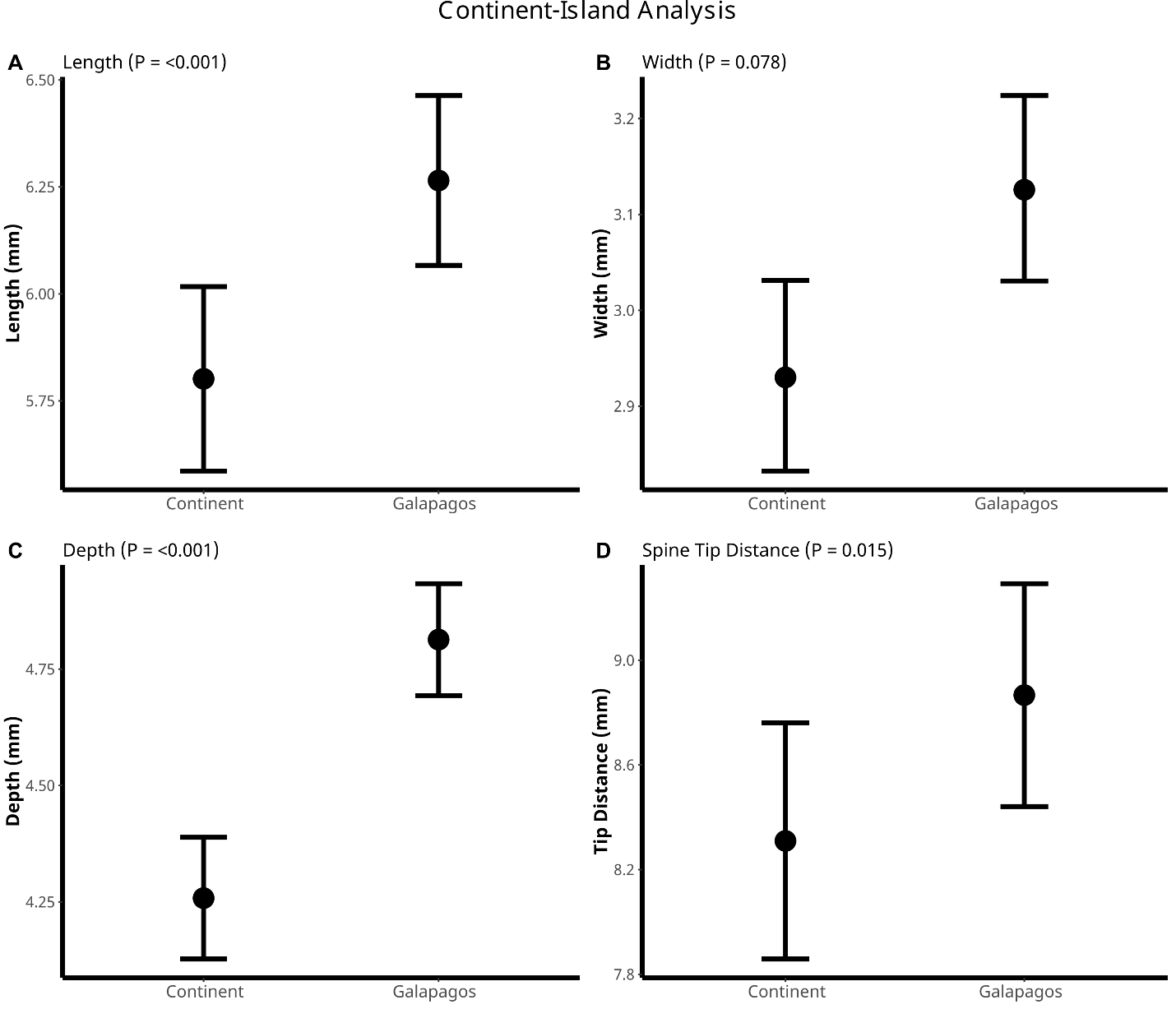


**FIGURE S17**

Individual mericarp traits compared between the Galápagos Islands and continental populations. Plots are the least-squares means ± one standard error. On top of each plot, it shows the p-values from the ANOVA. A-D) Mericarp trait plots without bioclimate variables.


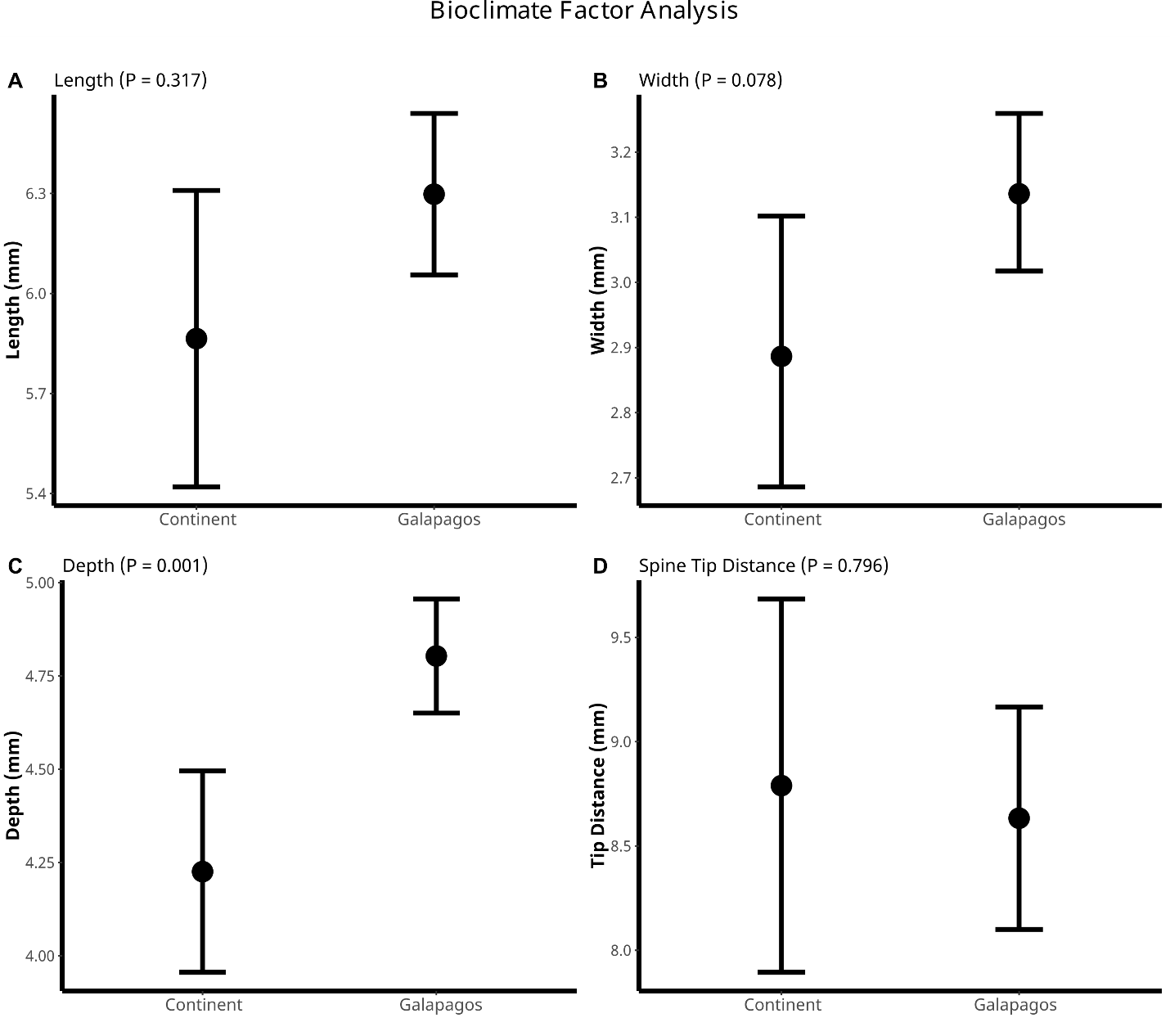


**FIGURE S18**

Individual mericarp traits compared between the Galápagos Islands and continental populations. Plots are the least-squares means ± one standard error. On top of each plot, it shows the p-values from the ANOVA. A-D) Mericarp trait plots include bioclimate variables.


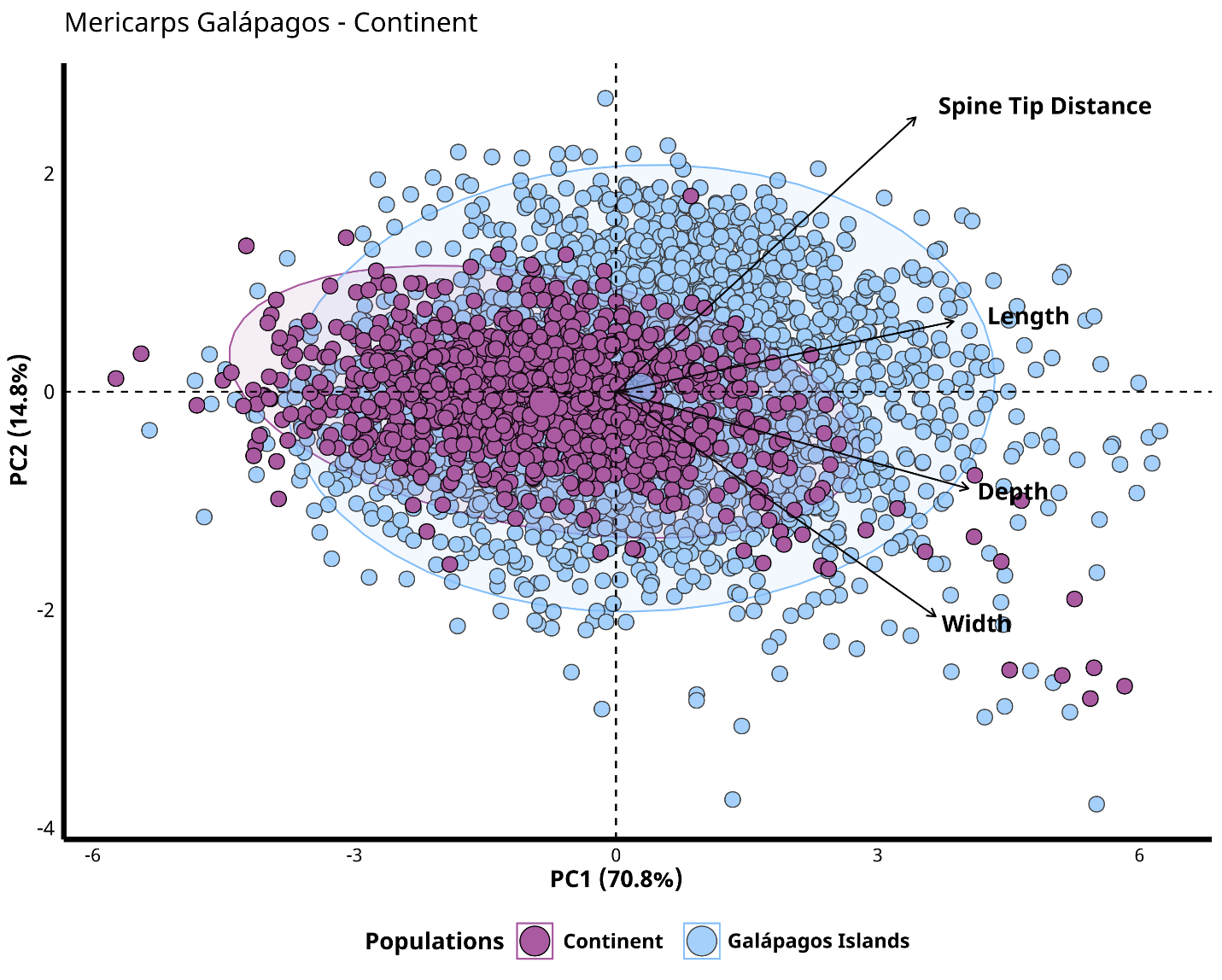


**FIGURE S19**

Principal component analysis of mean mericarp traits, length, depth, width, and spine size. Points represent individual mericarps from Galápagos and continental populations. Trait vectors are proportional to the contribution and direction associated to each trait. Larger circles represent the centroid of the ellipses with a 95% confidence interval.


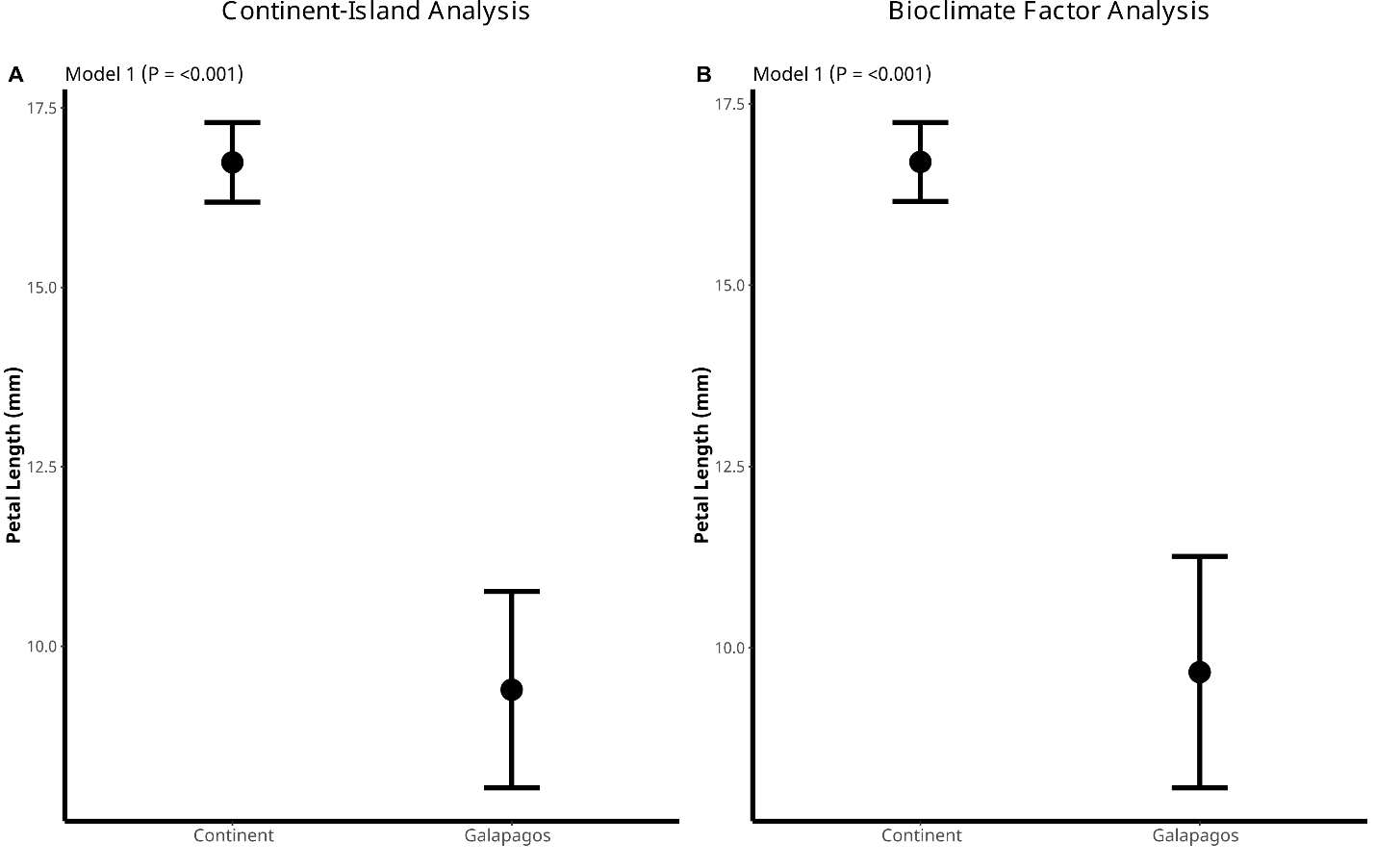


**FIGURE S20**

Petal length estimates from the Galápagos Islands and continental plants. The plots show the least-squares mean estimates (± 1 SE) using petal length. P-values are shown on top of each plot. (A) Estimates of continental and island populations only. (B) Estimates including bioclimate variables.


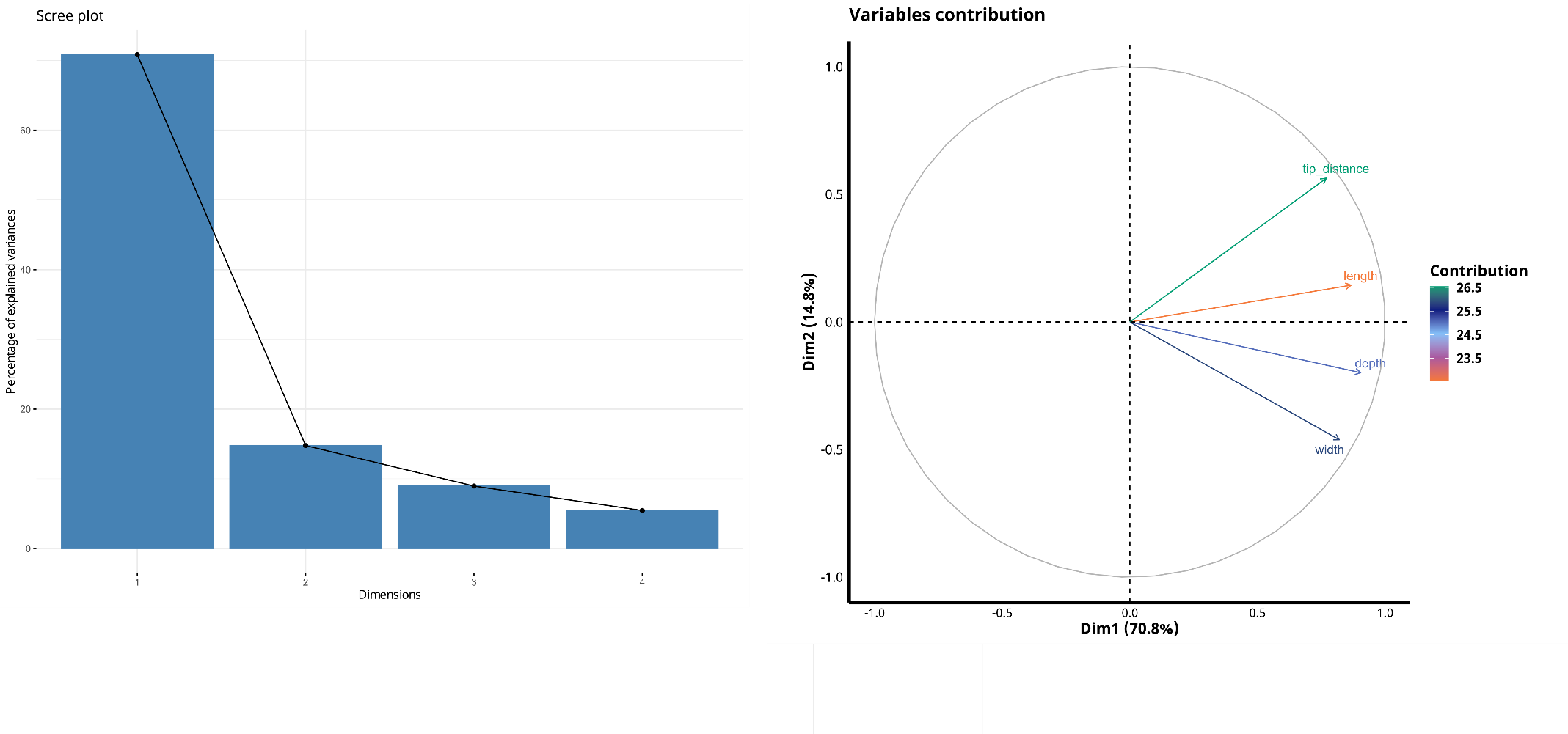


**FIGURE S21**

Eigenvector contribution plot and percentage of explained variation for each PC axis associated with mericarp morphology for Galápagos and continental populations.

**APPENDIX S8**


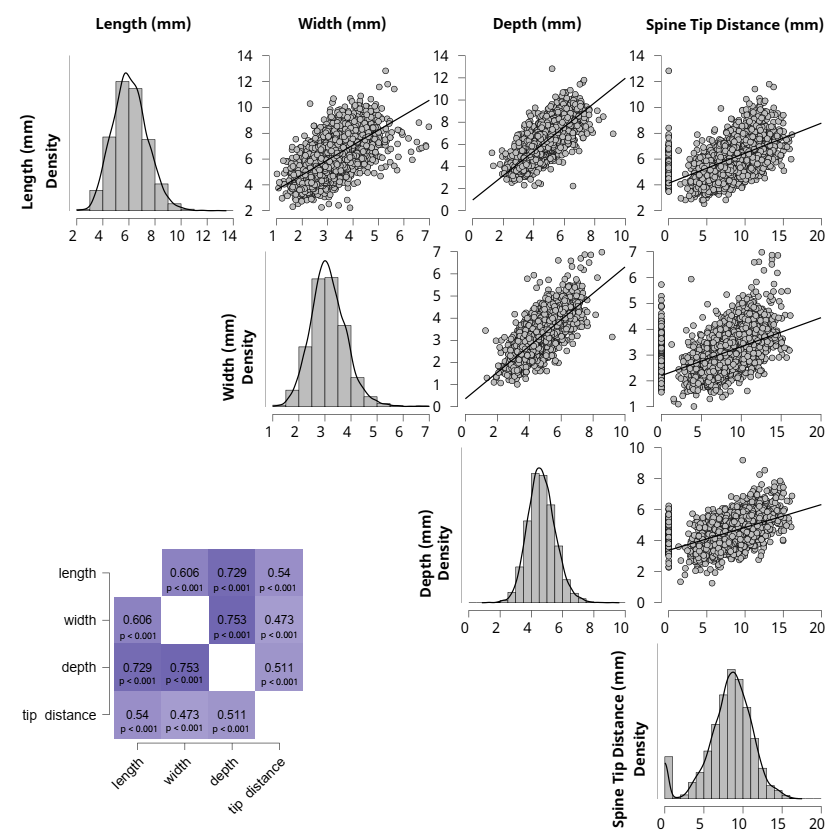


**Figure S21**

Distributions of mericarp traits and the correlations between traits. Upper right: The frequency distribution of each trait and the bivariate scatterplot between traits with the line of best fit show. Lower left: Symmetrical matrix of Pearson correlation coefficients between traits with heat darkness of colour corresponding to the strength of the correlation. All r-values are significant at P<0.001.

**appendix s9**


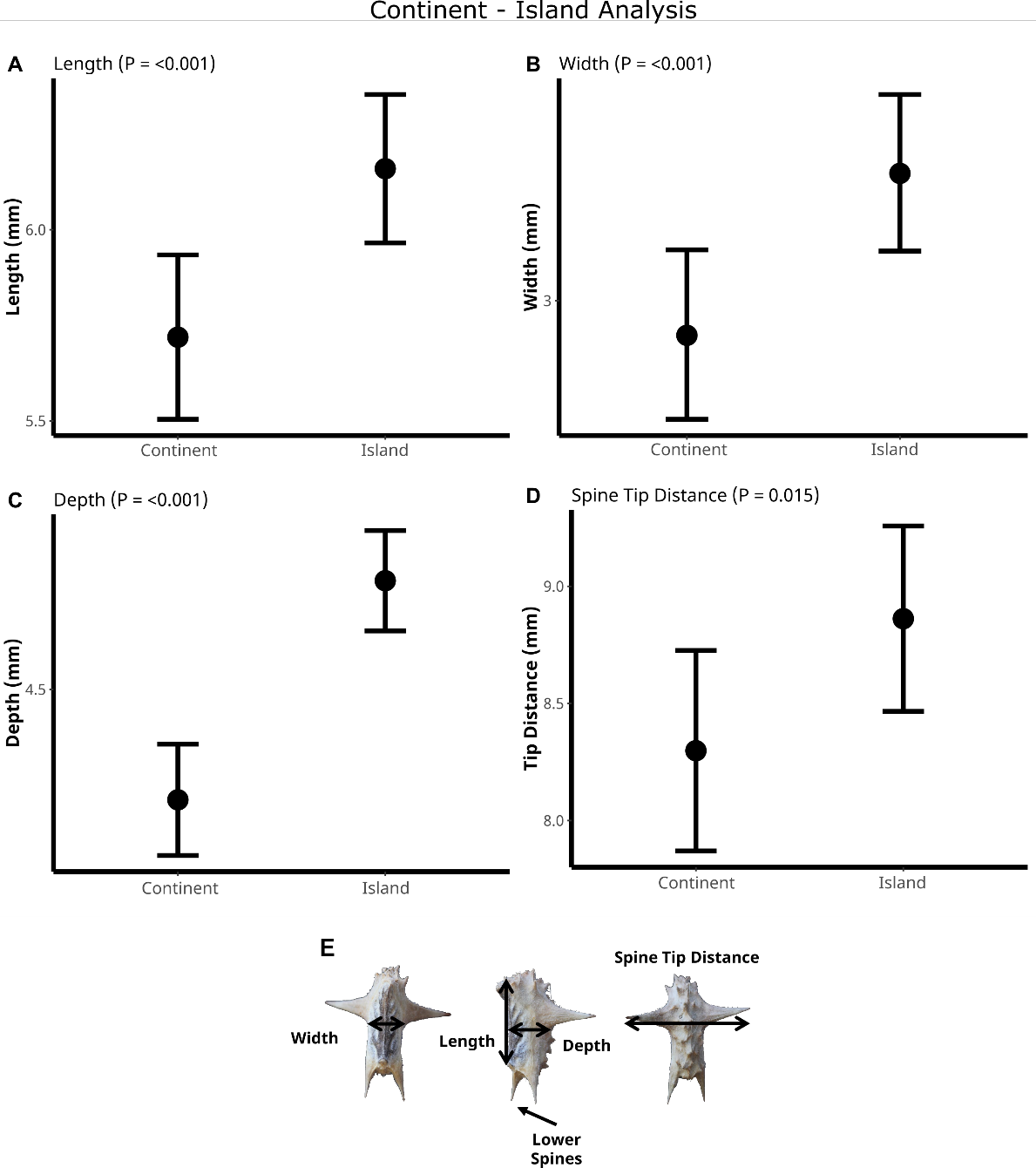


**Figure S22**

Mericarp traits compared between island and continental locations. Plots are the least-squares means ± one standard error. On top of each plot, it shows the p-values from the ANOVA. A-D) Individual mericarp trait plots. E) Diagram of how these traits were measured as described in Fig. 3.


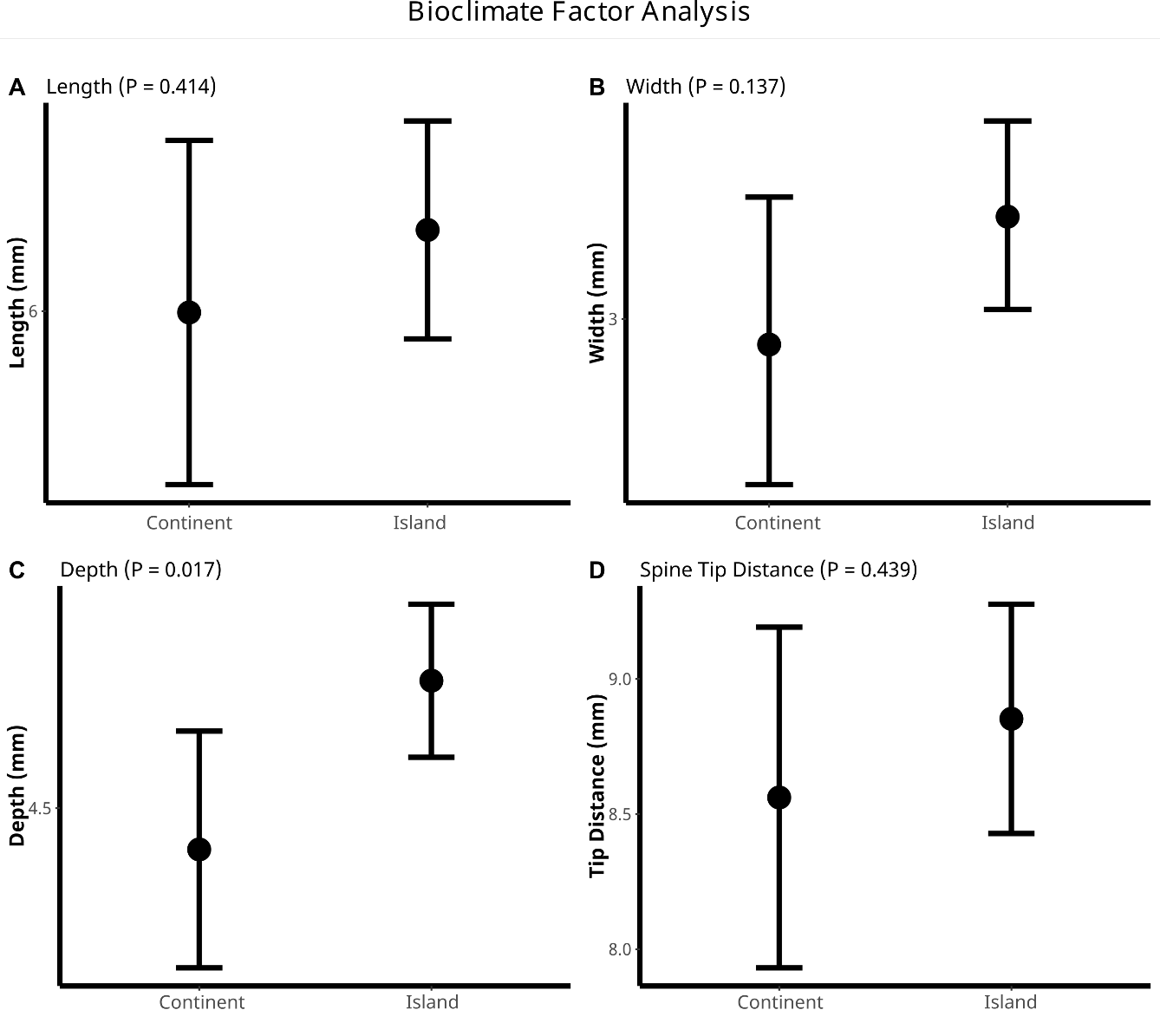


**Figure S23**

Mericarp traits compared between island and continental locations. Plots are the least-squares means ± one standard error. On top of each plot, it shows the p-values from the ANOVA. A-D) Individual mericarp trait plots including bioclimate variables.


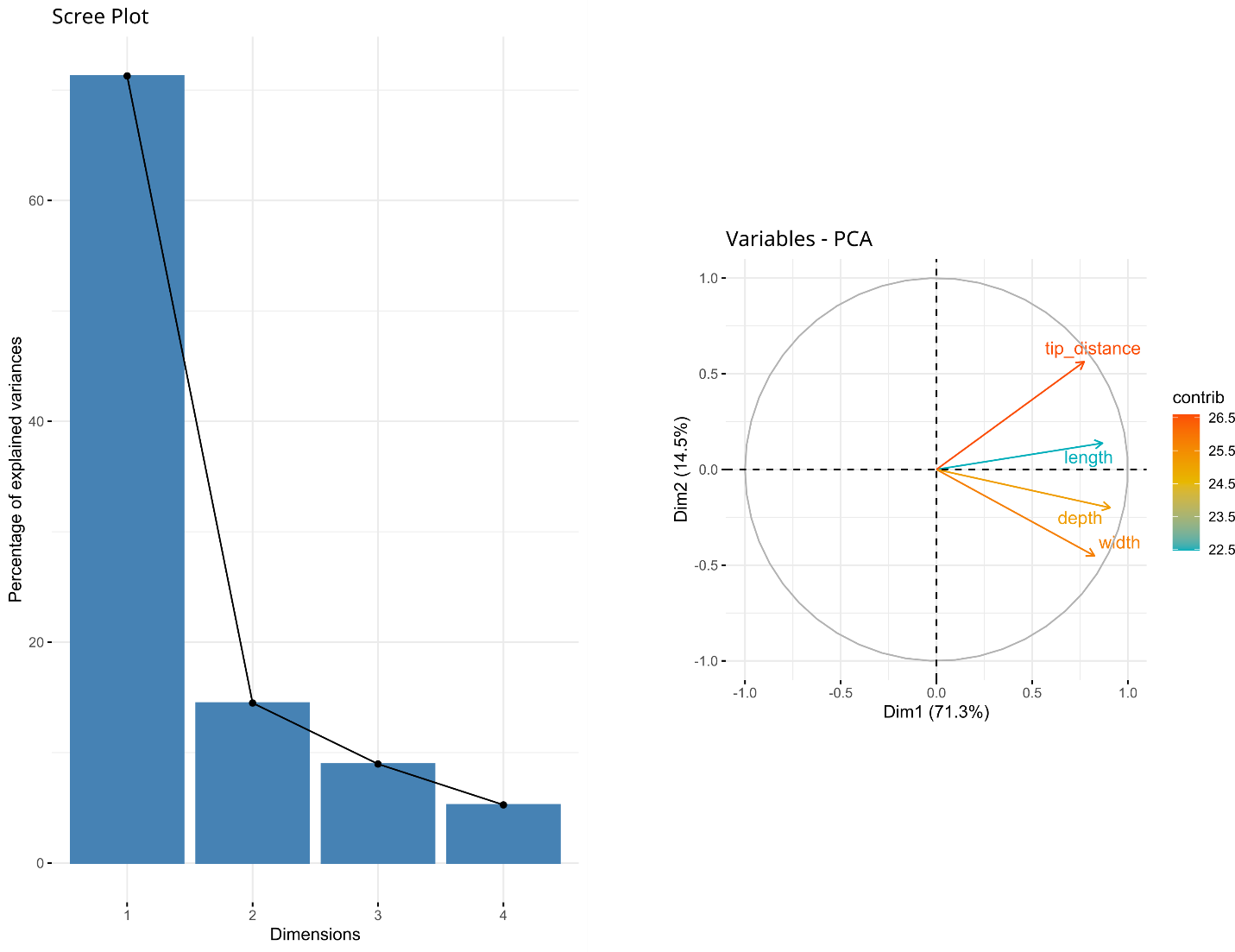


**FIGURE S9**

Eigenvector contribution plot and percentage of explained variation for each PC axis associated with mericarp morphology.

**APPEnDIX S10**


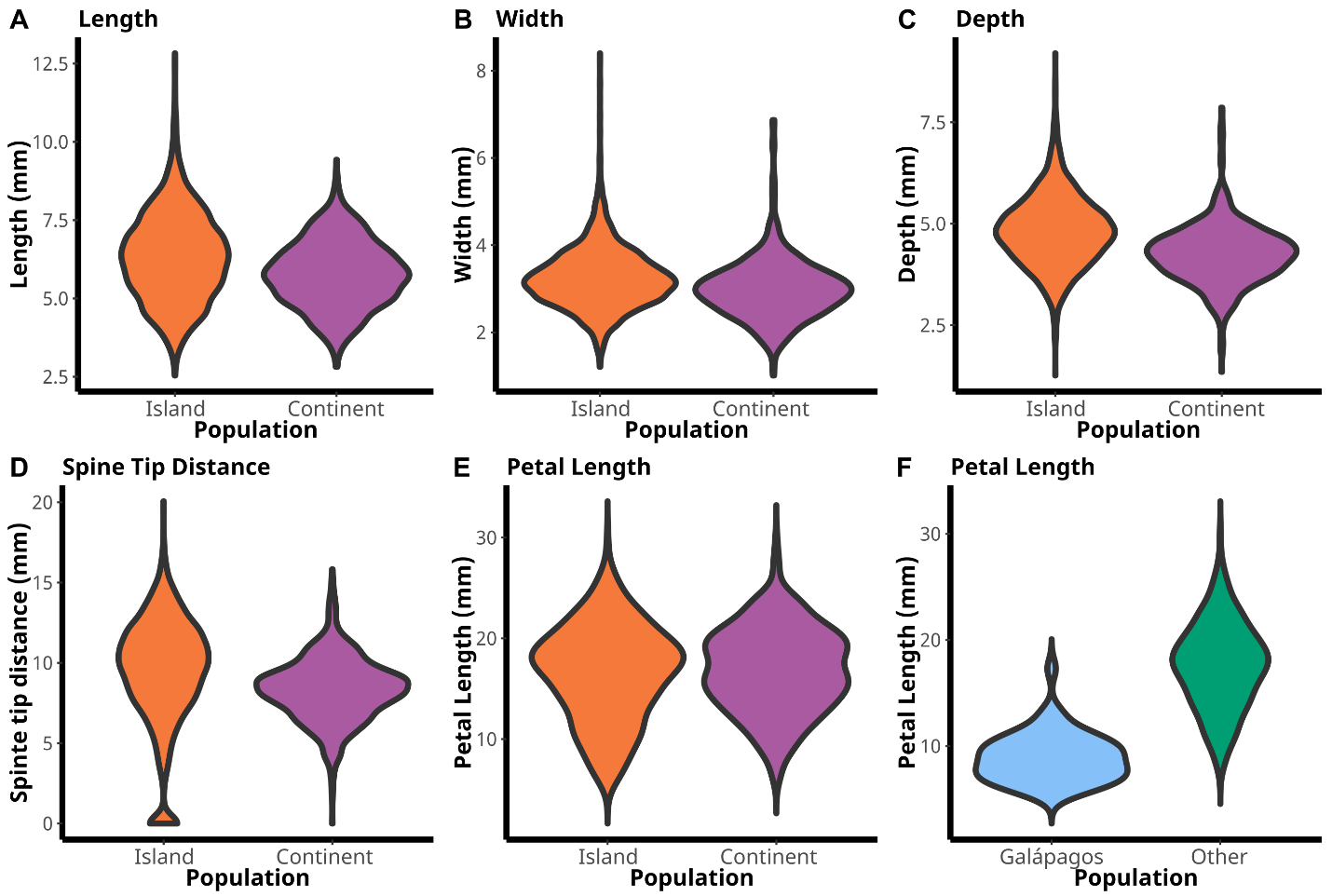


**Figure S24**

Trait distributions within measured traits groups shown as violin plots. Continental and island populations for mericarps and flowers (A-E) and for flower size between Galápagos and other islands (F).
